# Two Separate Interfaces between the Voltage Sensor and Pore Are Required for the Function of Voltage-Dependent K^+^ Channels

**DOI:** 10.1371/journal.pbio.1000047

**Published:** 2009-03-03

**Authors:** Seok-Yong Lee, Anirban Banerjee, Roderick MacKinnon

**Affiliations:** 1 Howard Hughes Medical Institute, Rockefeller University, New York, New York, United States of America; 2 Laboratory of Molecular Neurobiology and Biophysics, Rockefeller University, New York, New York, United States of America; Harvard Medical School, United States of America

## Abstract

Voltage-dependent K^+^ (Kv) channels gate open in response to the membrane voltage. To further our understanding of how cell membrane voltage regulates the opening of a Kv channel, we have studied the protein interfaces that attach the voltage-sensor domains to the pore. In the crystal structure, three physical interfaces exist. Only two of these consist of amino acids that are co-evolved across the interface between voltage sensor and pore according to statistical coupling analysis of 360 Kv channel sequences. A first co-evolved interface is formed by the S4-S5 linkers (one from each of four voltage sensors), which form a cuff surrounding the S6-lined pore opening at the intracellular surface. The crystal structure and published mutational studies support the hypothesis that the S4-S5 linkers convert voltage-sensor motions directly into gate opening and closing. A second co-evolved interface forms a small contact surface between S1 of the voltage sensor and the pore helix near the extracellular surface. We demonstrate through mutagenesis that this interface is necessary for the function and/or structure of two different Kv channels. This second interface is well positioned to act as a second anchor point between the voltage sensor and the pore, thus allowing efficient transmission of conformational changes to the pore's gate.

## Author Summary

Voltage-dependent ion channels open with a voltage dependence that is remarkably steep. This steep voltage dependence, which is essential to the propagation of nerve impulses, originates in the interaction between voltage-sensor domains of the ion channel and its pore. The voltage-sensor domains transmit voltage-driven conformational changes to the pore. To understand how this “electromechanical coupling” mechanism works, we have studied the protein–protein interfaces that connect the voltage sensors to the pore using bioinformatics, electrophysiological recordings, site-directed mutagenesis, and chemical cross-linking. We identify two functionally important interfaces: one links the mobile “voltage-sensor paddle” to the pore's gate near the intracellular membrane surface, while the other links an immobile region of the voltage sensor to the pore near the extracellular membrane surface. The two interfaces encompass only a small fraction of the voltage-sensor surface area, but appear to operate in unison to enable voltage-driven conformational changes within the voltage sensor so as to efficiently regulate the pore's gate.

## Abstract 3

Voltage-sensor domains contact the pore in potassium channels through two surfaces: one attaches the voltage sensor to the pore, while the other imparts force on the gate, in a process that is central to the generation of action potentials in nerve cells.

## Introduction

Voltage-dependent ion channels mediate electrical impulses and thus enable the rapid transfer of information along the cell surface. These impulses underlie information processing by the nervous system, muscle contraction, and many other important biological processes [1]. Members of the large family of voltage-dependent cation channels—including K^+^, Na^+^, and Ca^2+^ selective channels—all share a common architecture consisting of a central ion-conduction pore surrounded by four voltage sensors located on the perimeter. The atomic structures of voltage-dependent K^+^ channels (Kv channels), determined by x-ray crystallography, have provided the first detailed pictures of voltage-dependent ion channels [2–5]. Through the combination of atomic structural, biochemical, and electrophysiological data, we are beginning to decipher the principles by which voltage-dependent ion channels function as molecular-scale electromechanical coupling devices.

The pore entryway near the intracellular membrane surface is able to constrict (close) and dilate (open) through motions of S6 “inner helices” that define the pore entryway [6–8]. S4-S5 “linker helices” form a cuff surrounding the inner helices and connect the voltage sensors to the pore [4,7]. In the atomic structures of Kv1.2 and a mutant known as paddle chimera, the S4-S5 linker helices are positioned in such a manner that conformational changes within the voltage sensors can easily be transmitted to the inner helices in order to facilitate constriction or dilation of the pore [4,7].

The voltage sensors consist of four membrane-spanning helical segments named S1 through S4. S3 is actually two helices referred to as S3a and S3b. In all of the crystal structures determined, S3b forms with S4 a helix-turn-helix called the voltage-sensor paddle [2–5]. The S4 component of this paddle contains arginine residues that are distributed within the membrane electric field: this positioning of charged amino acids enables the transmembrane voltage to exert an electrostatic force on the voltage sensor, which can bring about conformational changes within the sensor. Accessibility studies in lipid membranes indicate that the S4 helix is displaced by approximately 15 Å across the membrane in association with the voltage-dependent conformational changes [9–11].

In this study, we address the issue of how conformational changes within the voltage sensor are transmitted to the pore. When the electric field within the membrane exerts force on the charged components of the voltage sensor, how is this force transmitted efficiently to the gate? The S4-S5 linker is essential, because it appears to act structurally as a mechanical lever on the pore's gate. But such an action would seem to require a second interface that would serve to fix the voltage sensor's position relative to the pore. We identified the second interface through statistical coupling analysis (SCA) of 360 Kv channel sequences [12–14], and we showed by experiment the importance of this interface to Kv channel function.

## Results

### Statistical Coupling Analysis of Kv Channels

**Figure 1:**
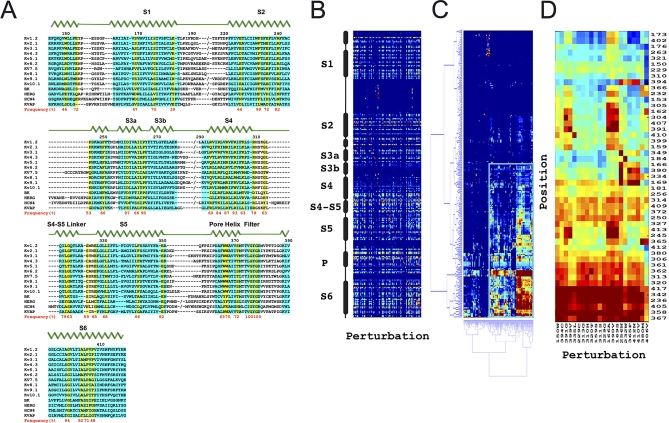
Statistical Coupling Analysis of the Kv Family (A) A representative alignment of Kv subfamilies out of the full sequence alignment of the Kv family that was used for the analysis. The sequences include Kv 1.2 (NCBI Entrez Protein Database [http://www.ncbi.nlm.nih.gov/sites/entrez?db=protein] GI: 52000923), Kv 2.1 (GI: 47523520), Kv 3.1 (GI: 5817540), Kv 4.3 (GI: 2935434), Kv 5.1 (GI: 20070166), Kv 6.2 (GI: 82997534), Kv 7.5 (GI: 8132997), Kv 8.1 (GI: 20381121), Kv 9.1 (GI: 112821679), Kv 10.1 (GI: 22164088), BK (GI: 157776), HERG (GI: 4557729), HCN (GI: 5734516), KvAP (GI:14601099). Conserved residues that were used to guide the alignment are highlighted in yellow, and their degrees of conservation are shown as frequency (%) in red (the frequency of the most common amino acid at the indicated position in the full sequence alignment) at the bottom of the alignment. The residue number corresponds to that of Kv 1.2. (B) A matrix representation of all pair-wise coupling values (termed ΔΔG^stat^ [14]) for 95 perturbations. The column and row show positions and perturbations from N to C terminus, respectively. The coupling values range from 0.5 kT* (blue) to 2 kT* (red) in units of “statistical energy” [12]. (C) Two-dimensional clustering of the matrix shows a group of positions and perturbations that are clustered together by the similarity of coupling patterns. (D) Focused clustering of an initially clustered group (box in (C)) reveals the final cluster of 49 residues and 22 perturbations.

**Figure 2:**
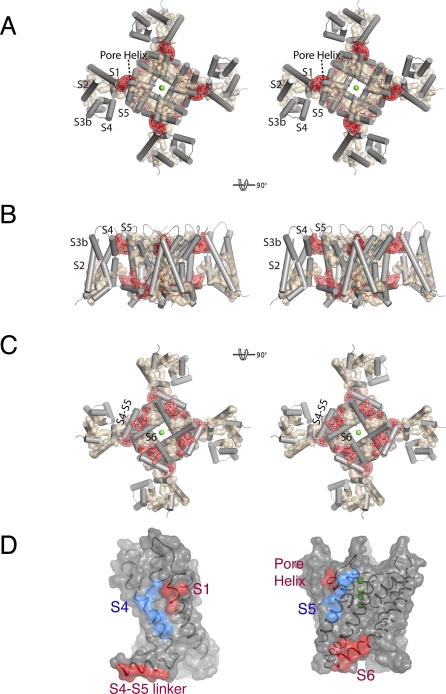
Mapping SCA-Derived, Co-Evolved Residues onto the Structure of the Paddle Chimera (PDB ID: 2R9R) The co-evolved residues identified in Figure 1D are shown in van der Waals surface (light brown). K^+^ ions are shown as green spheres. Stereo-views of the paddle chimera are shown in (A): looking from the extra-cellular side; in (B): from the side;, and in (C): from the intra-cellular side. The co-evolved residues form a structurally connected network where the inner membrane leaflet part of the voltage sensor is coupled to the pore via the S4-S5 linker. The co-evolved residues that are involved in the interfaces between the voltage-sensor and the pore are shown in red in (A), (B), and (C). (D) Surface representations of the interfaces between the voltage sensor and the pore in the structure of the paddle chimera. Shown separately are the voltage sensor from a single subunit and the tetrameric pore domain. The contact surfaces corresponding to the co-evolved interfaces between the S1 and the pore helix and the S4-S5 linker and S6 are colored red. The contact surfaces between the S4 and S5 are colored blue. The contact surfaces comprise residues that are within 4.0 Å distance.

Amino acid sequences of 360 Kv channels representing all Kv subfamilies were chosen by PSI-BLAST [15] (e-score < 0.001) and aligned by CLUSTALW [16] and structure-guided manual adjustment (Figure 1A). The sequences include Kv1 to Kv10, ERG, HCN, BK, bacteria, archea, and plant Kv channel families. A representative sequence alignment is shown (Figure 1A). SCA was performed using this alignment of 360 family members (see Materials and Methods) [14]. Figure 1B displays the degree to which the amino acid at a position in the sequence (vertical axis) is sensitive to constraint on the type of amino acid at another position (referred to as a perturbation, horizontal axis) using a color scale ranging from blue (insensitive) to red (sensitive) [14]. Regions of relative sensitivity (existence of co-evolved residues) and insensitivity (absence of co-evolved residues) are apparent. A cluster analysis was used to identify a self-consistent set of co-evolved residues (Figure 1B–1D) [14]; these are mapped onto the atomic structure of the paddle chimera channel (Figure 2A–2C) (Protein Data Bank [PDB; http://www.rcsb.org/pdb/home/home.do] ID 2R9R) [4]. As observed in other protein families, the co-evolved residues form a physically connected network (shown in van der Waals sphere) [12,14]. Overall, there are two interconnected layers within the co-evolved residue network. A top layer surrounds the pore's selectivity filter and extends into the S1 helix of the voltage sensor where S1 contacts the pore at the extracellular membrane surface (Figure 2A and 2B). A bottom layer includes the pore's S5 and S6 helices and extends into the intracellular half of the voltage sensor via the S4-S5 linker helix (Figure 2B and 2C). The S6 helices, also known as the inner helices, form the “activation” gate by opening and closing the pore at its cytoplasmic entryway. The bottom layer of the connected network defines a solid cuff surrounding this gate and extends to the voltage sensors (Figure 2B and 2C).

### The Voltage-Sensor Paddle Is Excluded from the Co-Evolved Set of Amino Acids

**Figure 3:**
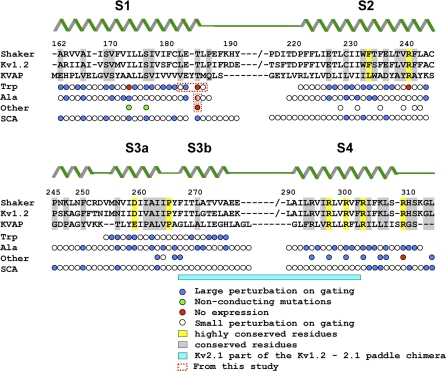
Comparison of Mutational Data with SCA Results Highly conserved residues (over 80 % identity in the multiple sequence alignment) are highlighted in yellow and are likely SCA-insensitive (see text). Moderately conserved residues (40–80% identity) are highlighted in gray. The impacts of mutations to Ala [17], Trp [22,23], or other residues within the voltage sensor are shown under the alignment. The mutations in the “Other” row are as follows: Arg or Leu or Pro on I173 and S176 on S1 [40], Val on T184 on S1; Asn on S2 and S3 [22,23]; Gln on S4 [41,42]; Val on L314 (S4) [43]. Blue filled circles represent mutations that cause a large perturbation in gating energy (>1 kcal/mol), whereas open circles indicate small perturbation in gating energy (<1 kcal/mol). Red filled circles represent no expression and green filled circles indicate expressed but non-conducting mutations. Half-red circle represents no expression in Shaker but small perturbation in Kv2.1. The cyan bar represents the Kv2.1 part of the paddle chimera. Mutations that are enclosed with dotted lines were produced and analyzed in this study. The numbering is based on rat Kv1.2.

A comparison of published mutagenesis data on Kv channels with SCA shows a good correlation over S1, S2, S3a, and the C-terminal extent of S4: when mutated, many residues identified by SCA have a large impact on channel gating (Figure 3). SCA is insensitive to near-absolutely conserved residues (highlighted yellow in Figure 3), because insufficient variation precludes detection of co-variation.

The voltage-sensor paddle (S3b and S4 through the fourth arginine) [2] is interesting in that mutations are known to have large effects on channel gating, but paddle residues are not part of the co-evolved set of amino acids (Figure 3). Involvement of the arginine positions may be undetectable due to a high degree of conservation. However the same is not true for other amino acid positions in the paddle that exhibit variation, are demonstrably important by mutation [17], and yet do not appear in the SCA defined co-evolved set of residues (Figure 3). Based on this analysis, we conclude that although the voltage-sensor paddle is important for the function of Kv channels, its residues (arginine excluded) are not co-evolved with amino acids elsewhere on the channel, either in the S1-S2 half of the voltage sensor or in the pore. The lack of co-evolution is consistent with the experiments of Swartz and colleagues demonstrating transferability of the voltage-sensor paddle among voltage sensors of different origins [18]. These properties of the voltage-sensor paddle—absence of co-evolved interfaces and transferability—seem compatible with the idea that the voltage-sensor paddle undergoes motion during channel gating.

### Two Interfaces between the Voltage Sensor and the Pore Identified by SCA

**Figure 4:**
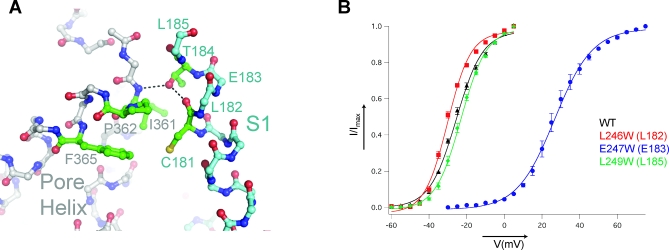
Voltage-Dependent Gating Properties of S1–Pore Interface Tryptophan Mutants (A) Close-up view of the S1–pore interface in the structure of paddle chimera (PDB ID: 2R9R). Pore helices are shown in gray and S1 helices are shown in cyan. Co-evolved residues (green) are shown in ball-and-stick representation. The residues corresponding to the ones mutated in Shaker have been labeled in blue. (B) Shown are mean ionic currents at 5-mV increments, normalized and fitted to a two-state Boltzmann (see Materials and Methods). The corresponding residues in Kv1.2 are given in parentheses.

The majority of co-evolved residues within the voltage sensor extend their side chains in toward its core rather than out toward its surface, consistent with the idea that the voltage sensor is largely an independent and self-contained domain [3,5] (Figure 2A–2C). There are however two exceptions: the S4-S5 linker, which appears “attached” to primarily the S6 helices near the cytoplasmic membrane surface (Figure 2C), and the S1 helix, which appears “attached” to the pore helix near the extracellular membrane surface (S1–pore interface) (Figure 2A and 2B). Mutational studies have shown that the S4-S5 linker is important for coupling voltage sensor motions to pore gating [19]. Presumably, this is why the S4-S5 linker interface contains co-evolved amino acids, so that it can form a “correct” interface with the S6 helices. The S1–pore interface in contrast to the S4-S5 linker interface has not been studied to a great extent: it includes three residues from the pore helix (I361, P362, and F365; the numbering is based on rat Kv1.2 unless otherwise stated) and two from the S1 helix (C181 and T184), all belonging to the co-evolved set (Figures 3 and 4A).

We note that the crystal structure shows physical contacts between the voltage sensor and pore that are more extensive than those defined by SCA (Figure 2D). For example, physical contacts exist between the S4 helix of the voltage sensor and the S5 helix of the pore (blue surface, Figure 2D), however, this “interface” does not contain co-evolved amino acids reaching across it.

### Disruption of the Interface between S1 and the Pore Helix in Shaker

The presence of co-evolved residues across the S4-S5 linker to S6 interface seems to corroborate previous studies demonstrating the importance of this region to channel gating. The S1–pore interface by contrast has not been studied in a highly systematic manner. The presence of co-evolved residues leads us to suspect that this second interface is important as well. To test this hypothesis, we ask two questions: How is the function of the channel affected when (1) the S1–pore interface is disrupted and (2) is constrained by a covalent cross-link?

A direct albeit crude method of testing the importance of a protein–protein interface is to disrupt the interaction by Trp or Ala mutation on one side of the interaction surface. Previous scanning mutagenesis studies have shown that mutations of the three residues (I361, P362, and F365) on the pore side of the S1–pore interface yield either nonfunctional channels or pronounced effects on gating (a shift in the equilibrium between closed to open states) [20,21]. In a Trp mutagenesis study of Shaker, Miller and co-workers showed that C245 in Shaker had a large impact on channel gating, consistent with the SCA analysis [22] (Figure 3). C245 (C181 in Kv1.2) is one of the two residues on S1 that form the S1–pore interface, the other residue being T248 (T184 in Kv1.2). However, the final C-terminal residue on S1 tested in their Trp scanning mutagenesis was L246 (L182 in Kv1.2). Hence we extended the Trp scanning experiments to include additional amino acids. Shaker RNAs containing L246W, E247W, T248W, and L249W in Shaker (L182, E183, T184, and L185, respectively, in Kv1.2) (Figure 4A) as well as wild-type were made and injected into oocytes, and ionic currents were measured using two-electrode voltage clamp (Figure S1). We used L246W as a control to reproduce the work from Hong and Miller. As shown in Figure 4B, the midpoint of activation (*V*_50_) of L246W is similar to that of wild-type as was reported [22] (Table 1). E247W mutation has a large impact on channel gating, as the *V*_50_ is shifted by more than 50 mV (Figure 4B and Table 1). This amino acid does not point into the interface but it does form a salt bridge to a gating charge Arg on S4 [4]. The large impact of the E247W mutation likely stems from destabilization of the open state by precluding formation of a salt bridge observed in the open conformation crystal structure. Mutation at the next position along the S1 helix, T248W, which points directly at the interface, leads to no detectable current (Table 1). This is an unusual outcome, because voltage sensors are in general rather tolerant to mutation: in combined experiments from different studies [22,23], only two positions—I237 (I173 in Kv1.2) and R297 (R240 in Kv1.2)—fail to tolerate mutation to Trp (Figure 3), and both of these are positioned in the core of the voltage sensor. The outcome of the T248W mutation could mean either that no channels are targeted to the cell membrane or that channels are present but not functional. Either result supports the importance of the S1–pore interface to channel structure and function. The next position along S1, L249W (L185 in Kv1.2), yielded functional channels in oocytes (Figure S1), and the *V*_50_ was similar to wild-type Shaker (Figure 4B and Table 1). Thus, we observe a sharp transition from nonfunctional to wild-type like behavior when we introduce the Trp residue adjacent to but not directly on the interface.

**Table 1:**
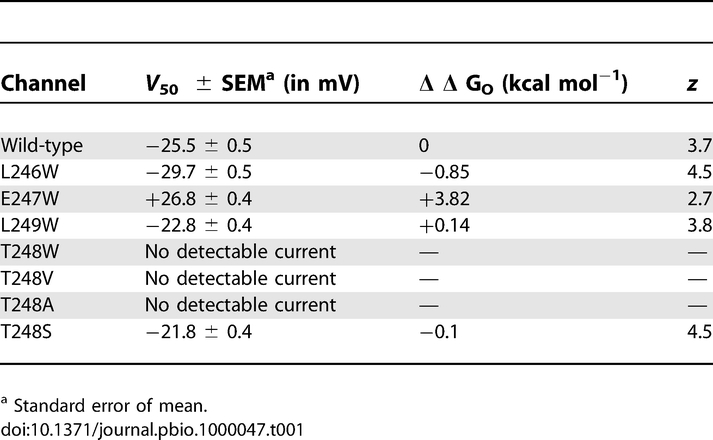
Voltage Activation Relations for S1 Interface Mutants in Shaker

**Figure 5:**
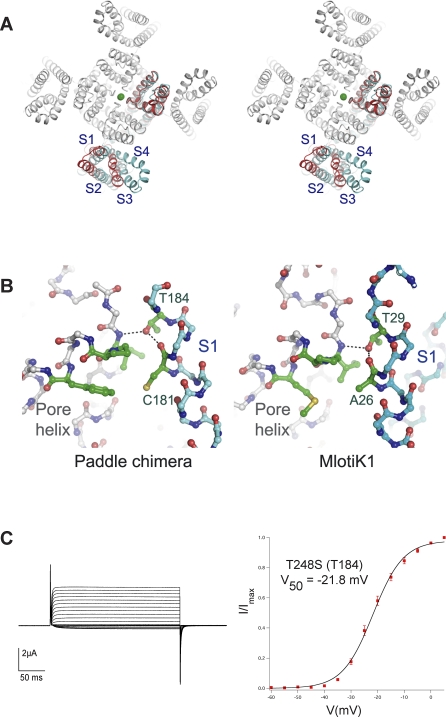
Chemistry of Interaction at the S1–Pore Interface (A and B) Comparison of the S1–pore interface between paddle chimera (PDB ID: 2R9R) and MlotiK1 (PDB ID: 3BEH). (A) Stereoview of superposition of the paddle chimera (cyan) and MlotiK1 (red) structures viewed from the extracellular side. Superposition was done using residues 337–344, 359–389 in paddle chimera, and the corresponding residues 142–149 and 164–194 in MlotiK1. (B) Close-up view of the S1–pore interfaces. Pore helices are shown in gray and S1 helices are shown in cyan. Co-evolved residues (green) are shown in ball-and-stick representation. (C) Voltage-dependent gating properties of T248S Shaker. The corresponding residue in Kv1.2 is denoted in parentheses. On the left are shown families of ionic currents at 5-mV increments, and on right, the normalized currents are fitted to a two-state Boltzmann. The holding voltage was −80 mV, tail voltage was −60 mV.

We also studied the S1–pore interface using an approach that would seem to be less disruptive than Trp substitution. The structure of a different 6-transmembrane (6-TM) channel, MlotiK1, was determined by Clayton et al. [24]. Although MlotiK1 is not a voltage-dependent K^+^ channel, it is related to Kv channels and has a similar architecture. Comparison of the MlotiK1 structure with the paddle chimera structure offers interesting clues concerning potentially important chemical interactions between S1 and the pore. A superposition of the paddle chimera and MlotiK1 structures made by aligning the pores shows that the S1–S4 domains coincide in space at only a single location, which corresponds to the interface between S1 and the pore (Figure 5A). In other words the S1–S4 domains adopt different orientations with respect to the pore, but the S1–pore interface is preserved. A more detailed comparison even shows chemical similarities within the S1–pore interfaces: the hydroxyl group of T184 in the paddle chimera (T29 in MlotiK1) forms a hydrogen bond with the backbone carbonyl oxygen of C181 (A26 in MlotiK1) on S1 (helix capping) and the backbone amide of I361 (numbering based on Kv1.2) (I162 in MlotiK1) on the pore helix (Figure 5B). This dual mode of interaction seems to require stringent specifications of the side chain functional group. Inspired by this observation, we generated further mutations in Shaker at T248 (T184 in Kv1.2) and tested them for channel function to examine the importance of the hydroxyl group at this position. Because T248W did not yield functional channels, we reasoned that if a tryptophan mutant introduces too severe a steric clash with the closely packed side chains in the surrounding region, replacement with an isosteric valine or a smaller alanine residue should be well tolerated. However, to our surprise, neither T248V nor T248A produced detectable currents (Table 1). This outcome is surprising, because Kv channels have been extensively studied with alanine scanning mutagenesis and very few mutations abolish function altogether [17]. On the other hand, replacement of T248 with serine resulted in functional channels, similar to wild-type Shaker (Figure 5C and Table 1). These data underscore the importance of the hydroxyl group at the S1–pore interface and the importance of the S1–pore interface to channel function.

### Constraining the S1–Pore Interface with a Disulfide Cross-Bridge

**Figure 6:**
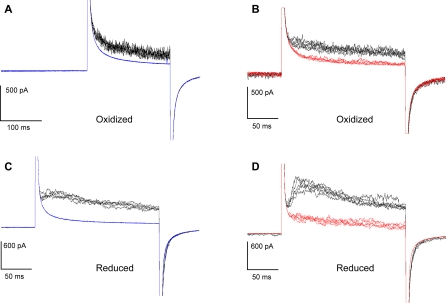
Representative Experiments Showing the Functional Effects of Disulfide Cross-Linking of S1–Pore Interface in KvAP Current traces were elicited after depolarization to positive voltages before (black) or after (blue) the addition of BaCl_2_ to the internal side, or before (black) and after (red) addition of CTX to the external side. (A and B) Air-oxidized 47Cys/183Cys KvAP. (C and D) DTT reduced 47Cys/183Cys (See Materials and Methods). Every experiment is from a separately painted membrane.

We further examined the S1–pore interface by covalently linking the two surfaces together through disulfide bridge formation. For these experiments, we turned to KvAP since it has been extensively tested in the bilayer system with mutagenesis and chemical modifications [9,11]. The use of a different Kv channel also allows us to assess whether the applicability of ideas concerning the S1–pore interface applies to Kv channels that are substantially different than Shaker. Five double cysteine mutants of KvAP containing one cysteine in S1 and another on the pore were tested. The channels were expressed in Escherichia coli, purified in the presence of detergent, reconstituted into lipid vesicles under reducing conditions, and then air-oxidized in the vesicles. Among the five combinations of double cysteine mutants (see Text S1 for the list), only one pair—T47C and V183C in KvAP (T184 and I361 in Kv1.2)—showed significant cross-linking of subunits on nonreducing SDS-PAGE (unpublished data), indicating that a disulfide bridge can be formed across the interface between S1 and the pore helix. Oxidized mutant channels in the planar bilayer system (see Materials and Methods) resulted in brief channel openings and small non-inactivating macroscopic currents (Figure 6A and 6B). Internal barium and external charybdotoxin, well-known K^+^ channel inhibitors, were used to confirm the identity of the channels as KvAP (Figure 6). In the reduced state (achieved by adding DTT to the channels in membrane vesicles prior to fusion with the bilayer), the same double mutant channels exhibited properties more similar to wild-type KvAP channels (Figure 6 C and 6D). Reduced channels are quickly converted back to oxidizing gating behavior by addition of oxidizing agent (Cu^2+^-phenanthroline) to the bilayer (unpublished data). These data indicate that a disulfide bridge across the interface is associated with an alteration of gating. In other words, channel gating is sensitive to reversible chemical modifications of the S1–pore interface.

## Discussion

This study was ultimately motivated by a puzzling feature of Kv channels revealed by the crystal structures: the voltage sensors exist as appendages without extensive contacts with the pore [4]. This being the case, how do voltage-driven conformational changes within the voltage sensors transmit mechanical forces onto the pore to open and close the gate? One region of contact in the crystal structures, that formed by the S4-S5 linkers and S6, appears to transmit motions of S4 to the gate [4,19]. A second region of contact in the crystal structures, that formed by S1 and the pore helix, was hypothesized to be important [4]. The present study tests this hypothesis with a statistical analysis of protein sequences and systematic experiments. We show that the S1–pore interface is indeed essential to Kv channel function.

We began by using SCA to identify co-evolved amino acids in Kv channels, in particular those crossing the interface between the voltage sensor and the pore. SCA reports information derived solely from sequence data and thus are independent of atomic structural data. We then map the set of co-evolved amino acids onto the atomic structure of the paddle chimera Kv channel in order to inspect their locations. We do not assume a priori that co-evolved amino acids identified by SCA are necessarily important. Instead, we use the SCA results to motivate new experiments and to interpret old experiments. In the end, based on experimental data, we find a strong correlation between co-evolution and importance to structure and/or function in the Kv channel family. Through this approach, we reach what we believe is a new insight into the function of Kv channels.

Many of the co-evolved amino acids identified by SCA have been studied in the past through mutation and are known to influence channel function (Figure 3). Amino acids in the pore surrounding the selectivity filter, when mutated, affect ion conduction and structure [25,26] as well as gating [21]. Mutations in the pore surrounding the S6 helix bundle crossing (gate) appear to influence a late-opening transition in gating [21]. Furthermore, co-evolved amino acids in the pore are coupled to each other in mutant cycle analysis of gating [21,27,28]. Thus, co-evolved amino acids in the pore in some cases appear to be important for the structure of the selectivity filter and in other cases for stabilizing conformations of the pore associated with gating states.

At two locations, the S4-S5 linker and the extracellular extent of S1, co-evolved residues cross the interface between the voltage sensor and the pore. It is well established through mutational studies that the S4-S5 linker plays an important role in coupling voltage-sensor action to pore gating. In their effort to attach functional voltage sensors to the non–voltage-dependent K^+^ channel KcsA, Lu and colleagues discovered that compatibility across the S4-S5 linker-to-pore interface is required (i.e., amino acids making both sides of this interface must come from the same voltage-dependent channel) [19]. The S4-S5 linker shows up in the co-evolved set of amino acids presumably because it is under selective pressure to link voltage sensor actions to pore gating through the protein–protein interface it makes with the pore (Figure 3).

The S1–pore interface identified by SCA analysis in the present study was not anticipated from past mutational studies, mainly because this region of Kv channels has not been studied in a highly systematic manner. In retrospect, several past mutational studies hinted at the importance of this region but no physical interpretation was provided [20–22]. The crystal structures of Kv1.2 and paddle chimera showed that S1 makes an apparently physically tight contact with the pore over a small area near the extracellular membrane surface [4,5]. Amino acids on both sides of the S1–pore interface turn out to be part of the co-evolved set (Figure 2). Experiments presented here demonstrate that this interface is important for channel function (Figures 4, 5, and 6). Through disruptive Trp substitution, through more subtle mutations of a specific hydrogen-bonding side chain hydroxyl in the Shaker K^+^ channel, and through disulfide cross-link oxidation and reduction in the KvAP channel, we demonstrate the functional importance of the S1–pore interface. It is worthwhile to note here that the sole purpose of our experiments with disulfide cross-linking at the S1–pore interface is to address the binary question whether this is a functionally important interface or not. Disulfide bond formation has been successfully used to probe three-dimensional proximity of different parts of proteins that might be distal in primary sequence. However, it is a fact that disulfide bonds can perturb local protein structure [29,30] and affect function. Given the chemically complicated nature of interactions formed at protein–protein interfaces, it is easy to see how placement of an engineered disulfide linkage might not allow the exact recapitulation of the native structural state and interactions. We attribute the difference between the oxidized and reduced versions of the mutant channel to such an effect. The point we wish to make is this: by constraining the S1–pore interface with a disulfide linkage, and by reversing the chemistry with reducing agents, we can affect the function of Kv channels. Thus, we conclude that the S1–pore interface is an important protein–protein contact for normal Kv channel function.

**Figure 7:**
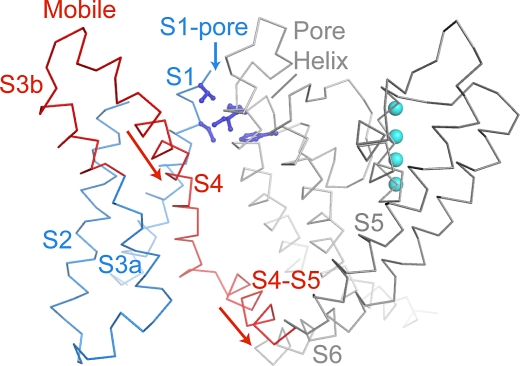
Model of Force Transmission in Kv Channels Shown is a complete monomeric subunit of the paddle chimera (PDB ID: 2R9R) and the pore region only from the adjacent subunit with which the S1–pore interface is formed. Components of the voltage sensor that are mobile along the transmembrane axis are shown in red, and components that are static along the transmembrane axis are shown in blue. The pore regions are shown in gray. The side chains of the residues forming the S1–pore interface are shown in blue ball and stick rendition, and the potassium ions lining the filter region are shown as cyan spheres. The blue arrow denotes the location of the S1–pore interface with respect to the entire protein structure. The red arrows indicate the putative direction of force transmission from the voltage sensor onto the S4-S5 linker and from the S4-S5 linker to the pore.

What might be the role of this interface? The crystal structures and associated functional studies assessing motion within voltage sensors have led to the hypothesis that with respect to motion along the transmembrane axis, voltage sensors contain a stationary half (S1, S2, and S3a) and a mobile half (S3b, S4, and S4-S5 linker) [4,7,11,31]. In this hypothesis, the electric field within the membrane exerts force on the charged S4 amino acids and brings about a motion of the mobile half: the voltage-sensor paddle (S3b-S4) is proposed to move about a “hinge” between S3a and S3b and exert a force onto the S4-S5 linker. The linker constricts (closes) or dilates (opens) the inner helix bundle (S6 helices) to gate the pore. In this view, the S1–pore interface might serve to brace the stationary half of the voltage sensor with respect to the pore, thus allowing a more efficient transference of force by the voltage sensor on the gate (Figure 7).

## Materials and Methods

### Sequence alignments.

Multiple amino acid sequences representing the Kv family were obtained from the non-redundant database using PSI-BLAST (e-score < 0.001) [15]. KvAP, Shaker, and BK channels were used for initial searches, and multiple iterations of PSI-BLAST were performed. Sequences that contain both the K^+^ channel selectivity filter (TVGYG or similar) and the voltage-sensor sequences were selected. Non–voltage-dependent channels (i.e., CNG and SK channels) were manually removed based on the annotation in the database. Three hundred and sixty Kv sequences were obtained and they include eukaryotic Kv1 to Kv10, ERG, HCN, BK, bacteria, archea, and plant Kv channel families from 107 species (see Text S2 for the list of species). The full sequence alignments are sufficiently diverse that positions with low conservation show amino acid frequencies near to their mean values found in all natural proteins [12,14]. The sequences were initially aligned using ClustalW [16] and manually adjusted based on the structures of KvAP and the paddle chimera. Only amino acids within the voltage sensor or pore regions (residues 144–417 in rat Kv 1.2) were included for alignment, whereas intracellular domains (i.e., T1, PAS, and CNB domains) were removed since they are not present in all Kv channels. Accessibility studies were used to aid in the alignment of S4 [32–35]. The full sequence alignment is provided as supporting information (Text S3).

### Statistical coupling analysis and clustering.

The code for the SCA was provided by S. W. Lockless (Rockefeller University). The calculation was performed as described [14]. The subalignment size cutoff value for the choice of perturbation was >0.4. This size cutoff value led us to choose 95 site-specific perturbations to build the statistical coupling matrix. Cutoff values from 0.35 to 0.45 did not change significantly the positions that form the final cluster. Two-dimensional hierarchical clustering of the matrix was carried out with MATLAB Ver. 6.1. (Mathworks) using the city-block distance metric as described [14]. The clustering algorithm is based on coupled two-way clustering analysis developed for gene microarray data [36]. After the first clustering, a sub-matrix (containing 143 positions and 59 perturbations) was extracted, and focused independent clustering was performed to refine the cluster. The second cluster was chosen based on the cutoff of the average perturbation value of 1 kT* in units of “statistical energy” [12].

### Mutagenesis, expression and electrophysiology of Shaker K^+^ channels.

Mutations were introduced in Shaker-IR [37] cDNA in the pBluescript KS (+) vector by the QuikChange method (Stratagene) and confirmed by sequencing the entire cDNA. These Shaker constructs were linearized with HindIII, and RNA was prepared by in vitro transcription with T7 RNA polymerase (Promega). mRNA was injected into Xenopus laevis oocytes, and K^+^ currents were recorded using a two-electrode voltage clamp (OC 725C, Werner Instrument Corporation) 1–3 d after injection. Data were filtered at 1 kHz (8-pole Bessel). Microelectrodes typically measured resistances in the range 0.3–0.8 MΩ when filled with 3 M KCl. Bath solution contained (in mM): 96 NaCl, 2 KCl, 0.3 CaCl_2_, 1 MgCl_2_, 5 HEPES (pH 7.6). Oocytes were typically held at −80 mV and stepped for 250 ms to different test voltages followed by repolarization. All experiments were carried out at room temperature. Voltage-activation curves were generated using the measured tail currents and fitted to a two-state Boltzmann equation:

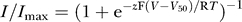
where *I*/*I*_max_ is the normalized tail current amplitude, *z* is the effective charge, *V*_50_ is the activation half voltage, F is the Faraday constant, R is the universal gas constant, and *T* is temperature.

### Preparation and cross-linking of mutant KvAP channels.

All mutagenesis was performed using the QuikChange method (Stratagene). The single native cysteine in KvAP was mutated to serine (C247S). Double cysteine mutant constructs were made on this cysteineless background. Mutant channels were expressed in E. coli and purified in the presence of detergents as described [38]. The channels were maintained under reducing conditions by the inclusion of 10 mM dithiothreitol (DTT) in the buffers following metal-affinity purification step. The purified channels were reconstituted in POPE : POPG (3:1) lipid vesicles as described elsewhere [39] in the presence of DTT. Following reconstitution, DTT was removed from the buffers and cross-linking was induced by air-oxidation and dialysis at room temperature for 3–5 d, and subsequently verified by non-reducing SDS-PAGE.

### Electrophysiology of mutant KvAP channels.

Electrophysiology of mutant KvAP channels was performed essentially as described [38], except that planar bilayer membranes were painted with 1,2-diphytanoyl-sn-glycero-3-phosphocoline (DPhPC). The channels were held at −120 mV and repeatedly pulsed to +120 mV test voltage. Experiments with charybdotoxin (CTX) and BaCl_2_ were carried out using the abovementioned protocol prior to and after the addition of external CTX (4 μM) and internal BaCl_2_ (2 mM). For testing the effect of reduction of the cross-link, the cross-linked channels were incubated with the presence of 50 mM DTT overnight at room temperature and then studied using the same protocols as described above.

## Supporting Information

Figure S1 Click here for additional data file. Families of Ionic Currents at 5-mV Increments
(A) WT Shaker. Holding voltage was −80 mV, tail voltage was −60 mV. (B) L246W Shaker. Holding voltage was −80 mV, tail voltage was −60 mV. (C) E247W Shaker. Holding voltage was −80 mV, tail voltage was −30 mV. (D) L249W Shaker. Holding voltage was −80 mV, tail voltage was −60 mV. The corresponding residues in Kv1.2 are labeled in parentheses.
(1.18 MB PDF)

Text S1 List of Double Cysteine Mutants Tested for Cross-Linking in KvAP The asterisk denotes a gap in the sequence of Kv1.2 in the sequence alignment. We think KvAP has an extra residue in this region that does not correspond exactly to any residue in Kv1.2 (see Figure 3). However one of the nearest neighbors is given as an approximate equivalent residue.
(34 KB DOC)

Text S2 List of Species (44 KB DOC)

Text S3 Sequence Alignment of 360 Kv Channels Each sequence begins with GI number. The sequence that starts with Kv_1221 is the sequence of the paddle chimera that was used for mapping of the results from SCA.
(376 KB DOC)

## Abbreviations

Kv channels: voltage-dependent K^+^ channels
SCA: statistical coupling analysis
