# Supplementary material for: Two Separate Interfaces between the Voltage Sensor and Pore Are Required for the Function of Voltage-Dependent K+ Channels
Source: PLoS Biol. 2009 Mar 3;7(3):e1000047. doi: 10.1371/journal.pbio.1000047 (PMC2650729; doi:10.1371/journal.pbio.1000047)
Supplement: Supplementary file 1 [file pbio.1000047.sg001.pdf]

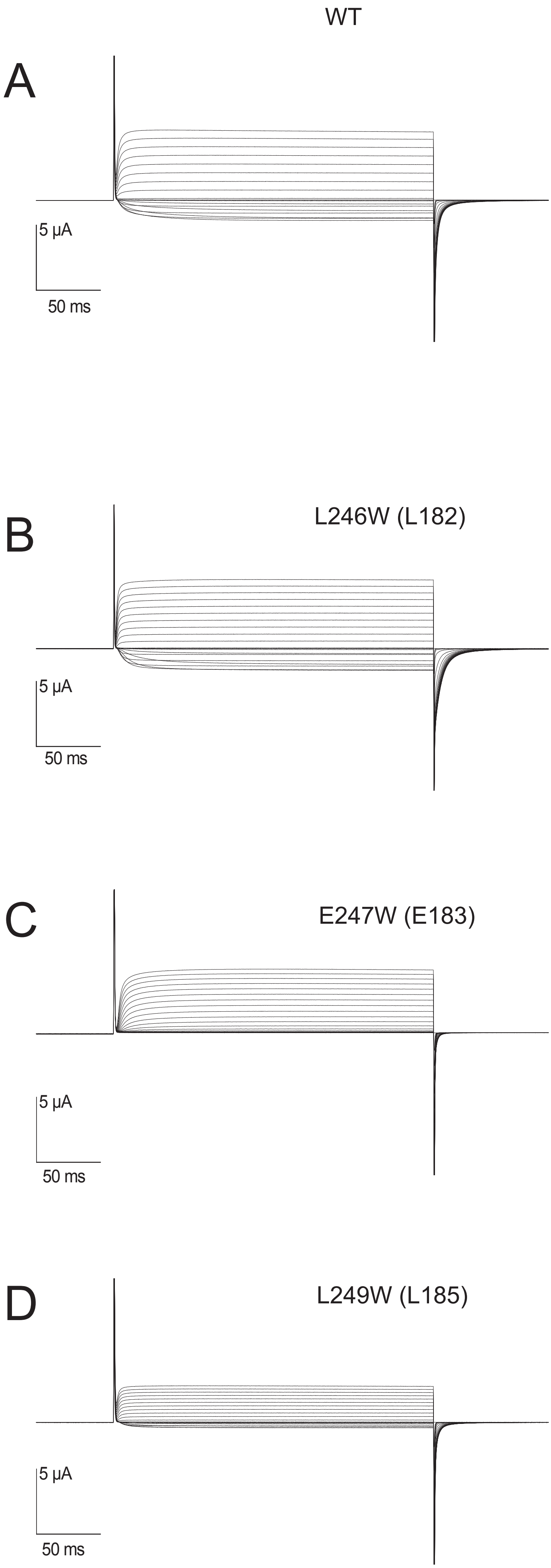
WT
A
5 μA
50 ms
B
L246W (L182)
5 μA
50 ms
C
E247W (E183)
5 μA
50 ms
D
L249W (L185)
5 μA
50 ms
