# Supplementary material for: Two Separate Interfaces between the Voltage Sensor and Pore Are Required for the Function of Voltage-Dependent K+ Channels
Source: PLoS Biol. 2009 Mar 3;7(3):e1000047. doi: 10.1371/journal.pbio.1000047 (PMC2650729; doi:10.1371/journal.pbio.1000047)
Supplement: Supplementary file 2 [file pbio.1000047.sd001.doc]

**Supporting Information**

**Text S1**

| **Double Cysteine mutants in KvAP** | **Corresponding numbering in rat Kv1.2** |
| --- | --- |
| V43C,L187C | C181,F365 |
| Y46C,V183C | T184*,F365 |
| Y46C,L187C | T184*,F365 |
| T47C,V183C | T184,I361 |
| T47C,187C | T184,F365 |

List of double cysteine mutants tested for crosslinking in KvAP. The asterisk denotes a gap in the sequence of Kv1.2 in the sequence alignment. We think KvAP has an extra residue in this region that does not correspond exactly to any residue in Kv1.2 (Figure 3 in main text). However one of the nearest neighbors is given as an approximate equivalent residue.
