# Supplementary material for: Two Separate Interfaces between the Voltage Sensor and Pore Are Required for the Function of Voltage-Dependent K+ Channels
Source: PLoS Biol. 2009 Mar 3;7(3):e1000047. doi: 10.1371/journal.pbio.1000047 (PMC2650729; doi:10.1371/journal.pbio.1000047)
Supplement: Supplementary file 3 [file pbio.1000047.sd002.doc]

**Supporting Information**

**Text S2**

**List of species**-

Aedes aegypti

Anopheles Gambia

Arabidopsis thaliana

Aeropyrum pernix

Aquifex aeolicus

Apteronotus leptorhynchus

Alcanivorax borkumensis

Aplysia californica

Anopheles gambiae

Bifidobacterium longum

Bacteroides fragilis

Bacteroides thetaiotaomicron

Bos Taurus

Columba livia

Chlorobium phaeobacteroides

Chlorobium limicola

Chromobacterium violaceum

Canis familiaris

Cancer borealis

Caenorhabditis elegans

Clostridium acetobutylicum

Chloroflexus aurantiacus

Danio rerio

Drosophila melanogaster

Desulfovibrio desulfuricans

Desulfitobacterium hafniense

Deinococcus radiodurans

Equus caballus

Flavobacterium sp.

Flavobacteria bacterium

Gallus gallus

Haemopis marmorata

Hirudo medicinalis

Halocynthia roretzi

Heliothis virescens

Homo sapiens

Ictalurus punctatus

Loligo opalescens

Loligo pealei

Lactobacillus reuteri

Manduca sexta

Mus musculus

Methanosarcina acetivorans

Methanococcoides burtonii

Magnetospirillum magnetotacticum

Macaca mulatta

Monodelphis domestica

Mycoplasma pulmonis

Mesocricetus auratus

Magnetococcus sp.

Mustela putorius furo

Methanosarcina mazei

Microbulbifer degradans

Nostoc punctiforme

Nostoc sp. PCC 7120

Oryctolagus cuniculus

Oncorhynchus mykiss

Oryctolagus cuniculus

Periplaneta Americana

Pseudomonas putida

Pseudomonas entomophila

Pseudomonas fluorescens

Pseudomonas syringae pv.

Pseudomonas aeruginosa

Pseudomonas mendocina

Panulirus interruptus

Polyorchis penicillatus

Panulirus interruptus

Pan troglodytes

Prochlorococcus marinus

Paramecium tetraurelia

Populus tremula

Rattus norvegicus

Ralstonia metallidurans

Rhodopseudomonas palustris

Streptomyces coelicolor

Streptomyces avermitilis

Streptococcus suis

Sus scrofa

Strongylocentrotus purpuratus

Shewanella denitrificans

Shewanella frigidimarina

Shewanella sp. MR-7

Shewanella sp. ANA-3

Shewanella sp. MR-4

Shewanella baltica

Shewanella woodyi

Shewanella pealeana

Salmonella typhimurium

Salmonella enterica

Schistosoma mansoni

Sternopygus macrurus

Samanea saman

Strongylocentrotus purpuratus

Squalus acanthias

Tetraodon nigroviridis

Trichodesmium erythraeum

Tribolium castaneum

Trachemys scripta

Vibrio splendidus

Vibrionales bacterium

Vibrio angustum

Vibrio cholerae

Xenopus laevis

Xylella fastidiosa

Xanthomonas campestris pv.

Xanthomonas axonopodis pv.
