# Supplementary material for: Two Separate Interfaces between the Voltage Sensor and Pore Are Required for the Function of Voltage-Dependent K+ Channels
Source: PLoS Biol. 2009 Mar 3;7(3):e1000047. doi: 10.1371/journal.pbio.1000047 (PMC2650729; doi:10.1371/journal.pbio.1000047)
Supplement: Supplementary file 4 [file pbio.1000047.sd003.doc]

```
15600964_1_220       ----------------KLTLRKKL-----------------------LEKVENTTETPMLILSVVYVVVALLPDIAVLS---------------------------------------------------------------------------------------------------------------------------------------------------------------------------------------------------------------------------------------------------------PDDLEFLDGLLWIVWGIFATELLVKIFVS-----------------PKPLQYMMQ----------------------NWPDVLIVAMPFL-------------------------------------------------------------------------------------------------------------------------------RPLRFLRILLVLPKAW--------------------------RQTKSV-----------LRQKTFSFIGLTS-----LSTVLLSAAFVYLV--EKG-------------------------------------------------------------------------TP------------------------------------------------------------------------SPINSYADALWWAMSTITTVGYGDMYPVTGFGRGVA-VFLMLTGITLFGLLTASVASFFVEDDTA--------------------------------
22971581_1_222       --------------TMSSEQRATL-----------------------LHRFERISELPMLLLSFGFLIIFLLVESNVFE---------------------------------------------------------------------------------------------------------------------------------------------------------------------------------------------------------------------------------------------------------ATITLVLDGLLWLIWGIFLAELVAKLYLA-----------------PDRLHYLRS----------------------HWFEVIIIVLPFL-------------------------------------------------------------------------------------------------------------------------------RPLRLLWLPIVLARLW--------------------------KQSQRA-----------LRRKMPAFIGVSS-----LVMVLITATLMFIA--ERG-------------------------------------------------------------------------SG------------------------------------------------------------------------GPITSFADAIWWALATITTVGYGDTYPVTALGRGIA-TFLMIAGIALFGLLTANVAAFFVEEDTV--------------------------------
21225475_20_246      -VRGTAARSRRRLSTMAR-MPDTNT----------------------TDLWERRTQGPLLALAVLFAVAYALPVVMP---------------------------------------------------------------------------------------------------------------------------------------------------------------------------------------------------------------------DAP-----------------------------------EQLLFACHVANWVVWAAFAIDYAVRLWLS-----------------EDRLRFVRS----------------------HPLALLAVLLPLL-------------------------------------------------------------------------------------------------------------------------------RPLRLLKLVSML-LLAG-------------------------QRARMA-----------SQVRVTTYVAGS--C---LGLLVFG-ALAVLEV-ER-------------------------------------------------------------------------GRPG-----------------------------------------------------------------------ASIQTLGDALWWAFTTMTTVGYGDMAPTTGLGRLLA-IGLMLSGIALLGVVTANIAAWFISR--F--------------------------------
29832342             --AGSPRPLRGALGTYDAGMKEQSA----------------------QARWEQHTQRPLLALAVAFAVAYAVPIVDS---------------------------------------------------------------------------------------------------------------------------------------------------------------------------------------------------------------------DAS-----------------------------------RSLTGVCTAVEWVVWGSFAADYLVRLAIT-----------------AERREFVRT----------------------HWLDLCAVVLPILQP-------------------------------------------------------------------------------------------------------------------------------LRLLRLVSTLMLLG--------------------------RRARMA-----------SQIRLTTYVAGSVI-----GLLMFG-SLAVLSV-ER-------------------------------------------------------------------------DSPN-----------------------------------------------------------------------GNIRTLGDAVWWSFTTMTTVGYGDHAPTTGLGRMIA-VGLMLSGIALLGVVTANIATWFISR--FEKDDVE--------------------------
46190713             -----------------------G-----------------------LKTWKKWTDWPLMVLSVVFLVVYSWEILS------------------------------------------------------------------------------------------------------------------------------------------------------------------------------------------------------------------------------------------------------------RTHIALCETVINVIWVVFVVDYVVSLWLA-----------------DDRWRWFKH----------------------NLFALLTIALPML-------------------------------------------------------------------------------------------------------------------------------RPLRLLRLLTVL-HVLN-------------------------RTSGMA-----------VRGRITVYAVGA------VGMLMYVGALAVYSV-ER-------------------------------------------------------------------------GASG-----------------------------------------------------------------------STITDFGTALWWAFVTVTTVGYGDFSPVTFQGKIIA-VVLMFTGIALIGIVT-TLASWIVDQVNL--------------------------------
146318766            ----------RNLPCNGGNMLKRLI---------------------RHPFYEKAMTYIAVVYVFLIVLELMDASIANS--------------------------------------------------------------------------------------------------------------------------------------------------------------------------------------------------------------------------------------------------------------SGYRVLDLLLWFIFVCDYFAQLYYA-----------------DDILEYVQG----------------------HILELIAIIPFDSFFSF--L--RL----------------------------------------------------------------------------------------G------------------------RLARLFRLVKVTRIF-ALSNRF------------------WDTINKLLHTNS------------LSKVLMLNI--------SAVLVSSVLLSVI--EG--------------------------------------------------------------------------KS-----------------------------------------------------------------------------FFDALWWSIVTMTTVGYGDIVPQDTISKIIA-ILLMLVGICTFGMVTSTINRFFFETERETK------------------------------
146320969            ----------RNLPCNGGNMLKRLI---------------------RHPFYEKAMTYIAVVYVFLIVLELMDASIANS--------------------------------------------------------------------------------------------------------------------------------------------------------------------------------------------------------------------------------------------------------------SGYRVLDLLLWFIFVCDYFAQLYYA-----------------DDILEYVQG----------------------HILELIAIIPFDSFFSF--L--RL----------------------------------------------------------------------------------------G------------------------RLARLFRLVKVTRIF-ALSNRF------------------WDTINKLLHTNS------------LSKVLMLNI--------SAVLVSSVLLSVI--EG--------------------------------------------------------------------------KS-----------------------------------------------------------------------------FFDALWWSIVTMTTVGYGDIVPQDTISKIIA-ILLMLVGICTFGMVTSTITRFFSETERETK------------------------------
92088991             ---------------NNINAAKEYI-------------------------YNISMALLAVISIVLIALDYAHEIDINA--------------------------------------------------------------------------------------------------------------------------------------------------------------------------------------------------------------------------------------------------------------MPYTAIDNAILIIFAIDYFVRLFSA-----------------KDKKTFFRD----------------------NVFDLLSIIPATEISSV--F--RL----------------------------------------------------------------------------------------A------------------------RIGRLLRILKILRIL-RLVG-L------------------TGRLEKFLKING------------LLYYLYTSI--------VVVLITSSLYCIS--ER--------------------------------------------------------------------------VS-----------------------------------------------------------------------------FSTAVWWSIVTSTTVGYGDISPETFIGKLAA-IIDMLIGIGLIGMLTSSITEYFSQSSNDE-------------------------------
15894597_1_228       ----------------NITTHNKI-------------------------LYELTSSLLALIASIMLIIELSVKLSNETLYIFD-------------------------------------------------------------------------------------------------------------------------------------------------------------------------------------------------------------------------------------------------------------TIDNIILIIFTIDYFLRLYIS-----------------KDKKKFFKE----------------------NIIDLLSIIPFNSIF-----------------------------------------------------------------------------------------------------------------------QGFRLLKISKLLKFTKLI-KFLKLF-MAFSLLLRF--------KSHFTKFVKTNN------------FHYVIYTTI---------FVLITGTIGMHLT-EG--------------------------------------------------------------------------LSLGN-----------------------------------------------------------------------------ALWWSFVTITTVGYGDISPSTPFGRVIA-SILMLIGIGFLSMLTGTISTFFISKKNG--------------------------------
23125946_1_240       ------------MLLSRQETEFYLK-------------DLETP----IGKAINLTLAALVLLSSGIFVAET-------YNIPDY------------------------------------------------------------------------------------------------------------------------------------------------------------------------------------------------------------------------------------------------------VRFQLHLVDTAIVIIFAVEYLLRLWSA-----------------ENKIQYVFSFY--------------------SIIDLMAILPFFLGMVDI--------------------------------------------------------------------------------------------------------------------------SFIRLLRWFRILRLI-RFIDRK-----------------FLFATISTED----------GMIFTRILFTL-------FAIVFIYSGLIYQV--EH------------------------------------------------------------------------PVNPQ------------------------------------------------------------------------NYGTFLDAFYFSVVTMTTVGFGDVIPISELGRLLT-VLMIFTGIALIPWQVGDLIKQVVKTANQ--------------------------------
17232125_1_240       ------------MLLSRQQTEFYLT-------------DLETP----LGKAINLTTAVLVLLSSGIFVAQT-------YAIPDS------------------------------------------------------------------------------------------------------------------------------------------------------------------------------------------------------------------------------------------------------IRLQLDQIDTVILVIFAVEYVLRLWSA-----------------ENKLKYIFSFY--------------------AIIDLIAILPFLLGLVNI--------------------------------------------------------------------------------------------------------------------------SFIRILRWFRILRLI-RFIDSK-----------------FLFGSISSED----------GIIFIRILFTL-------FTIIFVYSGLIYQV--EH------------------------------------------------------------------------PVNAQ------------------------------------------------------------------------VYSTFLDAFYFSVVTMTTVGFGDVTPISELGRLLT-VLMILTGVAIIPWQVGDLIRRVVKTANQ--------------------------------
16755829_52_371      -------KHLGWMTEAKDWAGELIS-------------GQSL-----TGRFLVLLVFILSIGSLIIYFYDASFQNFQVETCIPWQ------------------------------------------------------------------------------------------------------------------------------------------------------------------------------------------------------------------------------------------------------DSPSQQIDLGFNIFFLVYFFIRFIAA-----------------SDKVWFLLEMY--------------------SWIDFFTIPPSFVAIYLQRNWLGF--------------------------------------------------------------------------------------------------------------------LGFRFLRALRLMTVP-DILQYL--------------------NILKTSS----------SIRLTQLVTIFV-------AVCLTGAGLVHLL--EN------------------------------------------------------------------------SGDFFKG------------------------------------------------------------------FINPHRITYADSVYFVLVTMSTVGYGDIYCTTLCGRLFM-IFFILFGLAMFASYVPEIADLIGNRQ----------------------------------
72001309             ------EKHLGWMTEAKDWAGELIS-------------GQSL-----TGRFLVLLVFILSIGSLIIYFYDASFQNFQVETCIPW------------------------------------------------------------------------------------------------------------------------------------------------------------------------------------------------------------------------------------------------------QDSPSQQIDLGFNIFFLVYFFIRFIAA-----------------SDKVWFLLEMY--------------------SWIDFFTIPPSFVAIYLQRNW-----------------------------------------------------------------------------------------------------------------------LGFRFLRALRLMTVP-DILQYL--------------------NILKTSS----------SIRLTQLVTIFV-------AVCLTGAGLVHLL--EN------------------------------------------------------------------------SGDFFKG------------------------------------------------------------------FINPHRITYADSVYFVLVTMSTVGYGDIYCTTLCGRLFM-IFFILFGLAMFASYVPEIADLIGNRQKYGGEYKGEHG-----------------------
17566004_1_292       -----------KFFVAKDWAGELIS-------------GQSL-----TGRFLVLLVFILSIGSLIIYFYDASFQNFQVETCIPWQ------------------------------------------------------------------------------------------------------------------------------------------------------------------------------------------------------------------------------------------------------DSPSQQIDLGFNIFFLVYFFIRFIAA-----------------SDKVWFLLEMY--------------------SWIDFFTIPPSFVAIYLQRNWLGEHFWTHIRTNLGFFIFPFKNRTPDVLSFNLTPISAI---------------------------------------------------------------------------------FGFRFLRALRLMTVP-DILQYL--------------------NILKTSS----------SIRLTQLVTIFV-------AVCLTGAGLVHLL--EN------------------------------------------------------------------------SGDFFKG------------------------------------------------------------------FINPHRITYADSVYFVLVTMSTVGYGDIYCTTLCGRLFM-IFFILFGLAMFASYVPEIADLIGNRQ----------------------------------
21296709_85_338      ------DFEGTFMTEAKDWAGELIS-------------GQTT-----TGRILVVLVFILSIASLIIYFIDASNEE--VERCQKWS------------------------------------------------------------------------------------------------------------------------------------------------------------------------------------------------------------------------------------------------------NNITQQIDLAFNIFFMVYFFIRFIAA-----------------SDKLWFMLEMY--------------------SFVDYFTIPPSFVSIYLDRTW-----------------------------------------------------------------------------------------------------------------------IGLRFLRALRLMTVP-DILQYL--------------------NILKTSS----------SIRLAQLVSIFI-------SVWLTAAGIIHLL--EN------------------------------------------------------------------------SGDPLE-------------------------------------------------------------------FNNPQQLSYWTCVYFLIVTMSTVGYGDVYCETVLGRTFL-VFFLLVGLAMFASSIPEIIELVGS-----GS-----------------------------
118784160            ------DFEGTFMTEAKDWAGELIS-------------GQTT-----TGRILVVLVFILSIASLIIYFIDASNEE--VERCQKW------------------------------------------------------------------------------------------------------------------------------------------------------------------------------------------------------------------------------------------------------SNNITQQIDLAFNIFFMVYFFIRFIAA-----------------SDKLWFMLEMY--------------------SFVDYFTIPPSFVSIYLDRTW-----------------------------------------------------------------------------------------------------------------------IGLRFLRALRLMTVP-DILQYL--------------------NILKTSS----------SIRLAQLVSIFI-------SVWLTAAGIIHLL--EN------------------------------------------------------------------------SGDPLE-------------------------------------------------------------------FNNPQQLSYWTCVYFLIVTMSTVGYGDVYCETVLGRTFL-VFFLLVGLAIFASCIPEIIDLIGTRPKYGGTLKNERG-----------------------
25991361             ------EFEGTFMTEAKDWAGELIS-------------GQTT-----TGRILVVLVFILSIASLIIYFIDASSEE--VERCQKW------------------------------------------------------------------------------------------------------------------------------------------------------------------------------------------------------------------------------------------------------SNNITQQIDLAFNIFFMVYFFIRFIAA-----------------SDKLWFMLEMY--------------------SFVDYFTIPPSFVSIYLDRTW-----------------------------------------------------------------------------------------------------------------------IGLRFLRALRLMTVP-DILQYL--------------------NILKTSS----------SIRLAQLVSIFI-------SVWLTAAGIIHLL--EN------------------------------------------------------------------------SGDPLE-------------------------------------------------------------------FHNPQQLSYWTCVYFLIVTMSTVGYGDVYCQTVLGRTFL-VFFLLVGLAIFASSIPEIIELVGTRSKYGGEYKREHG-----------------------
91091496             ------EFEGTFMTEAKDWAGELIS-------------GQTT-----TGRILVVLVFILSIASLIIYFIDASNEG--VERCQEW------------------------------------------------------------------------------------------------------------------------------------------------------------------------------------------------------------------------------------------------------SNNITQQIDLAFNIFFMVYFLIRFIAA-----------------SDKLWFMLEMY--------------------SFVDYFTIPPSFVSIYLDRTW-----------------------------------------------------------------------------------------------------------------------IGLRFLRALRLMTVP-DILQYL--------------------NILKTSS----------SIRLAQLVSIFI-------SVWLTAAGIIHLL--EN------------------------------------------------------------------------SGDPLE-------------------------------------------------------------------FKNPNPLPYWTCVYFLIVTMSTVGYGDVYCHTVLGRTFL-VFFLLVGLAIFASSIPEIIDLVGTRSKYCGEYKREHG-----------------------
62472846             ------EFEGTFMTEAKDWAGELIS-------------GQTT-----TGRILVVLVFILSIASLIIYFVDASSEE--VERCQKW------------------------------------------------------------------------------------------------------------------------------------------------------------------------------------------------------------------------------------------------------SNNITQQIDLAFNIFFMVYFFIRFIAA-----------------SDKLWFMLEMY--------------------SFVDYFTIPPSFVSIYLDRTW-----------------------------------------------------------------------------------------------------------------------IGLRFLRALRLMTVP-DILQYL--------------------NVLKTSS----------SIRLAQLVSIFI-------SVWLTAAGIIHLL--EN------------------------------------------------------------------------SGDPLD-------------------------------------------------------------------FNNAHRLSYWTCVYFLIVTMSTVGYGDVYCETVLGRTFL-VFFLLVGLAMFASSIPEIIELVGSGNKYGGELKREHG-----------------------
157776               ------EFEGTFMTEAKDWAGELIS-------------GQTT-----TGRILVVLVFILSIASLIIYFVDASSEE--VERCQKW------------------------------------------------------------------------------------------------------------------------------------------------------------------------------------------------------------------------------------------------------SNNITQQIDLAFNIFFMVYFFIRFIAA-----------------SDKLWFMLEMY--------------------SFVDYFTIPPSFVSIYLDRTW-----------------------------------------------------------------------------------------------------------------------IGLRFLRALRLMTVP-DILQYL--------------------NVLKTSS----------SIRLAQLVSIFI-------SVWLTAAGIIHLL--EN------------------------------------------------------------------------SGDPLD-------------------------------------------------------------------FDNAHRLSYWTCVYFLIVTMSTVGYGDVYCETVLGRTFL-VFFLLVGLAMFASSIPEIIELVGSGNKYGGELKREHG-----------------------
48476133             ------EFEGTFMTEAKDWAGELIS-------------GQTT-----TGRILVVLVFILSIASLIIYFIDASSEE--VERCQKW------------------------------------------------------------------------------------------------------------------------------------------------------------------------------------------------------------------------------------------------------SDNITQQIDLAFNIFFMVYFFIRFIAA-----------------SDKLWFMLEMY--------------------SFVDYFTIPPSFVSIYLDRTW-----------------------------------------------------------------------------------------------------------------------IGLRFLRALRLMTVP-DILQYL--------------------NILKTSS----------SIRLAQLVSIFI-------SVWLTAAGIIHLL--EN------------------------------------------------------------------------SGDPLE-------------------------------------------------------------------FENAQKLSYWTCVYFLIVTMSTVGYGDVFCHTVLGRTFL-VFFLLVGLAMFASSIPEIIELVGSGSKYGGELKREHG-----------------------
62472908             ------EFEGTFMTEAKDWAGELIS-------------GQTT-----TGRILVVLVFILSIASLIIYFVDASSEE--VERCQKW------------------------------------------------------------------------------------------------------------------------------------------------------------------------------------------------------------------------------------------------------SNNITQQIDLAFNIFFMVYFFIRFIAA-----------------SDKLWFMLEMY--------------------SFVDYFTIPPSFVSIYLDRTW-----------------------------------------------------------------------------------------------------------------------IGLRFLRALRLMTVP-DILQYL--------------------NVLKTSS----------SIRLAQLVSIFI-------SVWLTAAGIIHLL--EN------------------------------------------------------------------------SGDPLD-------------------------------------------------------------------FNNAHRLSYWTCVYFLIVTMSTVGYGDVYCETVLGRTFL-VFFLLVGLAIFASCIPEIIDLIGTRAKYGGTLKNEKG-----------------------
62472912             ------EFEGTFMTEAKDWAGELIS-------------GQTT-----TGRILVVLVFILSIASLIIYFVDASSEE--VERCQKW------------------------------------------------------------------------------------------------------------------------------------------------------------------------------------------------------------------------------------------------------SNNITQQIDLAFNIFFMVYFFIRFIAA-----------------SDKLWFMLEMY--------------------SFVDYFTIPPSFVSIYLDRTW-----------------------------------------------------------------------------------------------------------------------IGLRFLRALRLMTVP-DILQYL--------------------NVLKTSS----------SIRLAQLVSIFI-------SVWLTAAGIIHLL--EN------------------------------------------------------------------------SGDPLD-------------------------------------------------------------------FNNAHRLSYWTCVYFLIVTMSTVGYGDVYCETVLGRTFL-VFFLLVGLAVFASWIPEITELAAQRSKYGGTYSKDPR-----------------------
134148768            -------------TEAKDWAGELIS-------------GQTT-----TGRILVVLVFILSIASLVIYFIDASNMDG-VEHCQPW------------------------------------------------------------------------------------------------------------------------------------------------------------------------------------------------------------------------------------------------------SANTTQQIDLAFNIFFMVYFFIRFIAA-----------------SDKLWFMLEMY--------------------SFVDYFTIPPSFVSIYLDRTW-----------------------------------------------------------------------------------------------------------------------IGLRFLRALRLMSVP-DILQYL--------------------NVLKTSS----------SIRLAQLCSIFI-------AVWLTGAGIIHLL--EN------------------------------------------------------------------------SGDPLT-------------------------------------------------------------------FENAHPLSYWTCVFFLIVTMSTVGYGDVYCQTVFGRTFL-VFFLLVGLAVFASWIPEITEIVGNRSKYGGEYKRSHG-----------------------
39545756             ------DPEIGWMTEAKDWAGELIS-------------GQTT-----TGRILVGLVFLLSIASLIIYFIDASTNTS-VETCLPW------------------------------------------------------------------------------------------------------------------------------------------------------------------------------------------------------------------------------------------------------SSSTTQQVDLAFNVFFMIYFFIRFVAA-----------------NDKLWFWVELF--------------------SFVDYFTIPPSFVAIYLDRNW-----------------------------------------------------------------------------------------------------------------------LGLRFLRALRLMSIP-DILTYL--------------------NVLKTST----------LIRLVQLVVSFV-------SLWLTAAGFLHLL--EN------------------------------------------------------------------------SGDPFFD------------------------------------------------------------------FGNAQHLTYWECLYFLMVTMSTVGFGDIFATTVLGRTFV-VIFIMIFIGLFASFIPEIAEILGKRQKYGGSYKKERG-----------------------
2662316_89_342       ------AAEVGWMTSVKDWAGVMIS-------------AQTL-----TGRVLVVLVFALSIGALVIYFIDSSNPI---ESCQNFY------------------------------------------------------------------------------------------------------------------------------------------------------------------------------------------------------------------------------------------------------KDFTLQIDMAFNVFFLLYFGLRSIAA-----------------NDKLWFWLEVN--------------------SVVDFFTVPPVFVSVYLNRSW-----------------------------------------------------------------------------------------------------------------------LGLRFLRALRLIQFS-EILQFL--------------------NILKTSN----------SIKLVNLLSIFI-------STWLTAAGFIHLV--EN------------------------------------------------------------------------SGDPWEN------------------------------------------------------------------FQNNQALTYWECVYLLMVTMSTVGYGDVYAKTTLGRLFM-VFFILGGLAMFASYVPEIIELIGNRK----------------------------------
2570854_64_383       ------AAEVGWMTSVKDWAGVMIS-------------AQTL-----TGRVLVVLVFALSIGALVIYFIDSSNPI---ESCQNFY------------------------------------------------------------------------------------------------------------------------------------------------------------------------------------------------------------------------------------------------------KDFTLQIDMAFNVFFLLYFGLRFIAA-----------------NDKLWFWLEVN--------------------SVVDFFTVPPVFVSVYLNRSW-----------------------------------------------------------------------------------------------------------------------LGLRFLRALRLIQFS-EILQFL--------------------NILKTSN----------SIKLVNLLSIFI-------STWLTAAGFIHLV--EN------------------------------------------------------------------------SGDPWEN------------------------------------------------------------------FQNNQALTYWECVYLLMVTMSTVGYGDVYAKTTLGRLFM-VFFILGGLAMFASYVPEIIELIGNRK----------------------------------
34333802_128_381     ------AAEVGWMTSVKDWAGVMIS-------------AQTL-----TGRVLVVLVFALSIGALVIYFIDSSNPI---ESCQNFY------------------------------------------------------------------------------------------------------------------------------------------------------------------------------------------------------------------------------------------------------KDFTLQIDMAFNVFFLLYFGLRFIAA-----------------NDKLWFWLEVN--------------------SVVDFFTVPPVFVSVYLNRSW-----------------------------------------------------------------------------------------------------------------------LGLRFLRALRLIQFS-EILQFL--------------------NILKTSN----------SIKLVNLLSIFI-------STWLTAAGFIHLV--EP------------------------------------------------------------------------SGDPWEN------------------------------------------------------------------FQNNQALTYWECVYLLMVTMSTVGYGDVYAKTTLGRLFM-VFFILGGLAMFASYVPEIIELIGNRK----------------------------------
1907289_47_366       ------AAEVGWMTSVKDWAGVMIS-------------AQTL-----TGRVLVVLVFALSIGALVIYFIDSSNPI---ESCQNFY------------------------------------------------------------------------------------------------------------------------------------------------------------------------------------------------------------------------------------------------------KDFTLQIDMAFNVFFLLYFGLRFIAA-----------------NDKLWFWLEVN--------------------SVVDFFTVPPVFVSVYLNRSW-----------------------------------------------------------------------------------------------------------------------LGLRFLRALRLIQFS-EILQFL--------------------NILKTSN----------SIKLVNLCSIFI-------STWLTAAGFIHLV--EN------------------------------------------------------------------------SGDPWEN------------------------------------------------------------------FQNNQQLTYWECVYLLMVTMSTVGYGDVYAKTTLGRLFM-VFFILGGLAMFASYVPEIIELIGNRK----------------------------------
3136118_24_343       ------AAEVGWMTSVKDWAGVMIS-------------AQTL-----TGRVLVVLVFALSIGALVIYFIDSSNPI---ESCQNFY------------------------------------------------------------------------------------------------------------------------------------------------------------------------------------------------------------------------------------------------------KDFTLQINMAFNVFFLLYFGLRFIAA-----------------NDKLWFWLEVN--------------------SVVDFFTVPPVFVSVYLNRSW-----------------------------------------------------------------------------------------------------------------------LGLRFLRALRLIQFS-EILQFL--------------------NILKTSN----------SIKLVNLCSIFI-------STWLTAAGFIHLV--EN------------------------------------------------------------------------SGDPWEN------------------------------------------------------------------FQNNQPLTYWECVYLLMVTMSTVGYGDVYAKTTLGRLFM-VFFILGGLAMFASYVPEIIELIGNRK----------------------------------
1305547_124_377      ------AAEVGWMTSVKDWAGVMIS-------------AQTL-----TGRVLVVLVFALSIGALVIYFIDSSNPI---ESCQNFY------------------------------------------------------------------------------------------------------------------------------------------------------------------------------------------------------------------------------------------------------KDFTLQIDMAFNVFFLLYFGLRFIAA-----------------NDKLWFWLEVN--------------------SVVDFFTVPPVFVSVYLNRSW-----------------------------------------------------------------------------------------------------------------------LGLRFLRALRLIQFS-EILQFL--------------------NILKTSN----------SIKLVNLLSIFI-------STWLTAAGFIHLV--EN------------------------------------------------------------------------SGDPWEN------------------------------------------------------------------FQNNQAPTYWECVYLLMVTMSTVGYGDVYAKTSLGRLFM-VFFILGGLAMFASYVPEIIELIGNRK----------------------------------
3452426_125_378      ------AAEVVWMTSVKDWAGVMIS-------------AQTL-----TGRVLVVLVFALSIGALVIYFIDSSNPI---QSCQNFY------------------------------------------------------------------------------------------------------------------------------------------------------------------------------------------------------------------------------------------------------KDFTLQIDMAFNVFFLLYFGLRFIAA-----------------NDKLWFWLEVN--------------------SVVDFFTVPPVFVSVYLNRSW-----------------------------------------------------------------------------------------------------------------------LGLRFLRALRLIQFS-EILQFL--------------------NILKTSN----------SIKLVNLLSIFI-------STWLTAAGFIHLV--EN------------------------------------------------------------------------SGDPWEN------------------------------------------------------------------FQNNQALTYWECVYLLMVTMSTVGYGDVYAKTTLGRLFM-VFFILGGLAMFASYVPEIIELIGNRK----------------------------------
1127824_23_342       ------AAEVGWMTSVKDWAGVMIS-------------AQTL-----TGRVLVVLVFALSIGALVIYFIDSSNPI---ESCQNFY------------------------------------------------------------------------------------------------------------------------------------------------------------------------------------------------------------------------------------------------------KDFTLQIDMAFNVFFLLYFGLRFIAA-----------------NDNLWFWLEVN--------------------SVVDFFTVPPVFVSVYLNRSW-----------------------------------------------------------------------------------------------------------------------LGLRFLRALRLIQFS-EILQFL--------------------NILKTSN----------SIKLVNLLSIFI-------STWLTAAGFIHLV--EN------------------------------------------------------------------------SGDPWEN------------------------------------------------------------------FQNSQALTYWECVYLLMVTMSTVGYGDVYAKTTPGGLFI-VFFILGGLAMFASYVPEIIEIIGNRK----------------------------------
14582152_56_375      ------VAEVGWMTSVKDWAGVMIS-------------AQTL-----TGRVLVVTVFALSIGALMIYFIDSSNPI---ESCQNFY------------------------------------------------------------------------------------------------------------------------------------------------------------------------------------------------------------------------------------------------------KDFTLQIDMAFNIFFLLYFGLRFIAA-----------------NDKLWFWLEVN--------------------SVVDFFTVPPVFVSVYLNRSW-----------------------------------------------------------------------------------------------------------------------LGLRFLRALRLIQFS-EILQFL--------------------NILKTSN----------SIKLVNLCSIFI-------STWLTAAGFIHLV--EN------------------------------------------------------------------------SGDPWRN------------------------------------------------------------------FENSQDLSYWECMYLLMVTMSTVGYGDVYAKTTLGRLFM-VFFILGGLAMFASYVPEIIELIGNRK----------------------------------
115621189            ------NEDVPWMTAIREYSASLIS-------------AQTK-----IGKCLVILSFTVSLGSFVIYILQLDEPI---ERCFSL------------------------------------------------------------------------------------------------------------------------------------------------------------------------------------------------------------------------------------------------------SEDHIFQVDLAFNIFFLLYFILRFIAA-----------------QDKFWFWLSLN--------------------SVVDFFTIPPIFVSIYMVRTW-----------------------------------------------------------------------------------------------------------------------LGLRFLRALRLMQLS-EILQFL--------------------SILRTSN----------SIKLLNLVTLLL-------TLWLGFAGIIHLV--EN------------------------------------------------------------------------SGDPWLQ------------------------------------------------------------------FANKQPLSYWTCIYFLVVTMSTVGYGDVYAKTVLGRIFA-TLFIFGGLALFATVVPEIYEILSQRGKYSGSLQSEKG-----------------------
120300928_79_331     ------LRKLYFHGVFRQRIEMLLS-------------AQTV-----VGQVLVILVFVLSIGSLVIYFINSMDPVR---RCSSY-------------------------------------------------------------------------------------------------------------------------------------------------------------------------------------------------------------------------------------------------------EDKIVHGDLSFNAFFSFYFGLRFWAA-----------------EDKIKFWLEMN--------------------SIVDIFTIPPTFISYYLKSNW-----------------------------------------------------------------------------------------------------------------------LGLRFLRALRLLELP-KILQIL--------------------QVIKTSN----------SVKLSKLLSIVI-------STWFTAAGFLHLV--EN------------------------------------------------------------------------SGDPWLN------------------------------------------------------------------GRNSQTMSYFESIYLVTATMSTVGFGDVVAKTSLGRIFI-VFFTLGSLILFANYIPEMVELFSTRK----------------------------------
20381097_79_331      ------VRSLHFQGQFRDHIEMLLS-------------AQTF-----VGQVLVILVFVLSIGSLIIYFINSADPVG---SCSSY-------------------------------------------------------------------------------------------------------------------------------------------------------------------------------------------------------------------------------------------------------EDKTIPIDLVFNAFFSFYFGLRFMAA-----------------DDKIKFWLEMN--------------------SIVDIFTIPPTFISYYLKSNR-----------------------------------------------------------------------------------------------------------------------LGLRFLRALRLLELP-QILQIL--------------------RAIKTSN----------SVKFSKLLSIIL-------STWFTAAGFIHLV--EN------------------------------------------------------------------------SGDPWLK------------------------------------------------------------------GRNSQNISYFESIYLVMATTSTVGFGDVVAKTSLGRTFI-MFFTLGSLILFANYIPEMVELFANKR----------------------------------
15838027_1_260       -----------TNSGWRRHWFEIV-------------YRHDTP----PSRNFDLLLMLAILGSVLVVMLDSVLALQAH------------------------------------------------------------------------------------------------------------------------------------------------------------------------------------------------------------------------------------------------------------WKDFLYALEWLFTLIFTADYLIRLVVV-----------------QQRLRYVLSIW--------------------GIIDLLSILPTYLSLFMPGTQSLL------------------------------------------------------------------------------------------------------------------IVRVLRLLRLFRILKMT-HYVEES--------------------GELVQALWR--------SRRKVMLFICTV------MTIAMIAGATMYVI--EG-------------------------------------------------------------------------PQH-------------------------------------------------------------------------GFTSIPTGMYWAIVTMTTVGFGDITPQSTLGRFVT-SALILIGYSIIAVPTGIYTAELTQGLLR--------------------------------
52856624_1_260       -----------TNSGWRRHWFEIV-------------YRHDTP----PSRNFDLLLMSAILGSVLVVMLDSVLILQAH------------------------------------------------------------------------------------------------------------------------------------------------------------------------------------------------------------------------------------------------------------WKYFLYTLEWLFTLIFTADYLIRLVVV-----------------QQRLRYVLSIW--------------------GIIDLLSILPTYLSLFMPGTQSLL------------------------------------------------------------------------------------------------------------------IVRVLRLLRLFRILKMT-HYVEES--------------------GELIQALWR--------SRRKVMLFLCTV------MTIAMIAGATMYVI--EG-------------------------------------------------------------------------PQH-------------------------------------------------------------------------GFTSIPTGMYWAIVTMTTVGFGDITPQSTLGRFVT-SALILIGYSIIAVPTGIYTAELTQGLLR--------------------------------
21232712_10_260      -----------TTDGWRRQWFDII-------------YRHDTR----PSRNFDLLLVVTILTSVVVIMIDSVPRIHAQ------------------------------------------------------------------------------------------------------------------------------------------------------------------------------------------------------------------------------------------------------------SAHWLVPLEWAFTVVFTIEYALRLAVV-----------------RRPPRYALSIW--------------------GVIDLLSILPSYLSFFVPGAQTLL------------------------------------------------------------------------------------------------------------------VVRVLRILRLFRILKLT-RYVEES--------------------GQLLDALWR--------SRRKVLVFLFTV------LTITVIAGATMYVI--EG-------------------------------------------------------------------------PQH-------------------------------------------------------------------------GFTSIPTSMYWAIVTMATVGFGDLVPQTTLGRFVT-SALILIGYSIIAVPTGIYTAELASNLRR--------------------------------
21244155_1_260       -----------TDDGWRRQWFDII-------------YRHDTR----PSRNFDLMLVVAILASVVVIMIDSVPRIHSH------------------------------------------------------------------------------------------------------------------------------------------------------------------------------------------------------------------------------------------------------------AADWLVPLEWAFTVIFTIEYALRLSVV-----------------RRPLHYALSVW--------------------GVIDLLSILPSYLSFFVPGAQTLL------------------------------------------------------------------------------------------------------------------VVRVLRILRLFRILKLT-RYIEES--------------------GQLLDALWR--------SRRKVLVFLFTV------LTITVIAGATMYII--EG-------------------------------------------------------------------------PQH-------------------------------------------------------------------------GFTSIPTSMYWAIVTMATVGFGDLVPQTTLGRFVT-SALILIGYSIIAVPTGIYTAELASSLRE--------------------------------
23121342_110_360     -----------TESGWRRRWFDII-------------YRHDTP----SSRNFDLLLIGLILASVLVIMGDSVPRFHAR------------------------------------------------------------------------------------------------------------------------------------------------------------------------------------------------------------------------------------------------------------WSGWLYGLEWIFTVLFTIEYGLRLAVV-----------------RRPWRYALSSW--------------------GLIDLLSILPTYLSLLIPGSQSLL------------------------------------------------------------------------------------------------------------------VVRVLRMLRLFRILKLT-QYVEEG--------------------GMLVGALWR--------SRRKIFVFISAV------LTVVVIFGALMYVV--EG-------------------------------------------------------------------------PEH-------------------------------------------------------------------------GFTSIPTGMYWAIVTMATVGFGDIAPATPLGRFIA-SVLIPDRLQRDRGADRHLQRRAGQQPAR--------------------------------
22977967_1_267       -----------PESGWRQRWYTII-------------FEADTR----PGRTFDIVLLVAIIASVAVVMLDSIQAIHAR------------------------------------------------------------------------------------------------------------------------------------------------------------------------------------------------------------------------------------------------------------LGTVFTALEWLFTLLFTTEYIMRLSVV-----------------RRPLRYVFSFY--------------------GIIDFLSIIPTWASFFVPELAFLI------------------------------------------------------------------------------------------------------------------DVRLLRLLRVFRILKLT-VYFEEA--------------------EILMLALSN--------SRRKIFVFLGTV------FIITVILGTIMYVV--EG-------------------------------------------------------------------------PTH-------------------------------------------------------------------------GFTSIPVSMYWAIVTLTTTGFGDMVPKTPIGQFIT-SITILLGYGIIAFPTGIVGAELAASIIR--------------------------------
34499017             -----------PLSGWRRKLYIII-------------YEADTR----AGRIFDMALIVAILISVACVMLESVSSIRQRYG------------------------------------------------------------------------------------------------------------------------------------------------------------------------------------------------------------------------------------------------------------TLLAVLDWLFTILFTIEYALRLICV-----------------HKPMRYVKSFF--------------------GVIDLLSILPLYLGLFIPES--RF----------------------------------------------------------------------------------------------------------------LADIRILRLLRMFRILKLS-RHLGEA--------------------AQLQKALLA--------SRRKITVFLATV------ILLVIVLGTLMYVI--EG-------------------------------------------------------------------------PEH-------------------------------------------------------------------------GFTSIPISIYWAIVTLTTVGFGDITPKTPMGQALA-SLVMITGYGIIAVPTGIVSAEIARSAAPEPSDNR--------------------------
21229291             -----------PDDDWRNTLYTII-------------FEADTP----AGKRFDEILIVTILLSVIVVMLDSVKEIAASYG------------------------------------------------------------------------------------------------------------------------------------------------------------------------------------------------------------------------------------------------------------DLFYSLEWIFTIMFTVEYLLRLVCV-----------------GRPVRYATSFF--------------------GIIDLIAILPAYFSLLLPGS--EY----------------------------------------------------------------------------------------------------------------LLVIRSLRLLRIFRVLKLV-QYLGEA--------------------DLLIRALRA--------SRRKITLFLFTV------LNLVVILGSLMYVI--EG-------------------------------------------------------------------------GKN-------------------------------------------------------------------------GFTSIPTSVYWAVITLTTVGYGDIVPETGLGQAVA-SVVMITGYSIIAVPTGIITSEISYASRYTK------------------------------
20090882             -----------PENNWRNTLYTII-------------FEADTP----AGKLFDEVLILTILLSVIVVMLDSVSGIAAVYG------------------------------------------------------------------------------------------------------------------------------------------------------------------------------------------------------------------------------------------------------------GLFYILEWIFTILFTVEYFLRLICV-----------------GRPLRYATSFF--------------------GIIDLLAILPTYLSLLLPGS--RY----------------------------------------------------------------------------------------------------------------LLVIRSLRLLRIFRVLKLV-QYVGEA--------------------DLLIKALQA--------SRRKITLFLFAV------LNLVVILGSLMYVI--EG-------------------------------------------------------------------------AES-------------------------------------------------------------------------GFTSIPRSIYWAVITLTTVGYGDIVPVTNLGQALA-SVIMIIGYSIIAVPTGIVTSEITFASKNLN------------------------------
91773675             -----------ECEGWRSYMYKYI-------------FEANTP----IGKYFDITLILTIILSVIVVMLDSVHSISSIHH------------------------------------------------------------------------------------------------------------------------------------------------------------------------------------------------------------------------------------------------------------QKLYMAEIIFTILFTIEYLLRMICV-----------------KSKVRYATSFF--------------------GIIDLLAIIPTYLSFFFPGS--QY----------------------------------------------------------------------------------------------------------------LLIIRVFRLLRIFRVLKLV-QYLTEA--------------------DMLVQALKD--------SSKKITVFTFTV------LNLVLILGSLMYVI--EG-------------------------------------------------------------------------EQN-------------------------------------------------------------------------GFTSIPRSVFWAITTLTTVGYGDLVPVTPLGQAMA-SVVMLLGYSIIAVPTGIITHSIINTSMAARPIMISEKT----------------------
23473800_5_255       -----------PVHGWRRTLHEII-------------FEADTP----AGRLFDIILIIAILFSVLVVLLDSVTTVRMR------------------------------------------------------------------------------------------------------------------------------------------------------------------------------------------------------------------------------------------------------------YAGLLFTLEWLFTLLFTVEYILRLLCV-----------------LRPAAYARSFF--------------------GVVDLLAILPTWATLLLPGT--HYLA----------------------------------------------------------------------------------------------------------------VVRLLRVLRIFRVLKLA-QYVSEA--------------------RHLIRALRD--------SRRKILVFLLAV------LSLVTIFGTLMYVI--EG-------------------------------------------------------------------------EKY-------------------------------------------------------------------------GFTSIPRSMYWAVVTLTTVGYGDISPGTPLGQGIA-SFIMLLGYGIIAVPTGIVTSALVRP-RP--------------------------------
148546804            -----------NPASLRERLFVIV-------------FQTDTV----AGRRFDKILLLIILASLVTVILDSIDEVHQGYA------------------------------------------------------------------------------------------------------------------------------------------------------------------------------------------------------------------------------------------------------------GLLAGIEWGFTAIFLAEYITRLYCS-----------------PKPLRYAFSFY--------------------GLVDLLAIVPGILALYYSDA--QY----------------------------------------------------------------------------------------------------------------LLIIRVIRMLRIFRVLKLS-PYLKQA--------------------HYLMEALRG--------SKQKIIVFLVTV------STLVTVFGTLMYVI--EG-------------------------------------------------------------------------PEH-------------------------------------------------------------------------GFTSIPKGIYWAIVTLTTVGFGDIVPKTPLGQVLS-SLVMITGYSIIAVPTGIFTAELANAMRGE-------------------------------
126357202            -----------AQQSLRERLYIMV-------------FQTDTV----AGRRFDKILLLIILASLVTVILDSIDEVHQGYA------------------------------------------------------------------------------------------------------------------------------------------------------------------------------------------------------------------------------------------------------------GLLAAIEWGFTAIFLAEYITRLYCS-----------------PKPLRYAFSFY--------------------GLVDLLAIVPGIIALYYSDA--QY----------------------------------------------------------------------------------------------------------------LLIIRVIRMLRIFRVLKLS-PYLKQA--------------------HYLMEALRG--------SKQKIIVFLVTV------STLVTVFGTLMYVI--EG-------------------------------------------------------------------------PEH-------------------------------------------------------------------------GFTSIPKGIYWAIVTLTTVGFGDIVPKTPLGQVLS-SLVMITGYSIIAVPTGIFTAELANAMRGE-------------------------------
104780825            -----------NPSSLRERLYVIV-------------FQTDTV----AGRRFDKILLLIILASLVTVILDSIDDVHRNYA------------------------------------------------------------------------------------------------------------------------------------------------------------------------------------------------------------------------------------------------------------GLLAGIEWGFTAIFLAEYITRLYCS-----------------PKPLRYAFSFY--------------------GLVDLLAIVPGIIALYYSDA--QY----------------------------------------------------------------------------------------------------------------LLMIRVIRMLRIFRVLKLS-PYLKQA--------------------HYLLAALQG--------SKQKIIVFLVSV------STLVTVFGTLMYVI--EG-------------------------------------------------------------------------PEH-------------------------------------------------------------------------GFTSIPKGIYWAIVTLTTVGFGDIVPKTPLGQVLS-SLVMITGYSIIAVPTGIFTAELANALRGE-------------------------------
119856792            -----------SSQSLRERLYVIV-------------FQTDTV----AGRRFDKTLLLIILASLVTVILDSIDEIHQNYA------------------------------------------------------------------------------------------------------------------------------------------------------------------------------------------------------------------------------------------------------------GLLAGIEWGFTAIFLAEYITRLYCS-----------------PKPMRYAFSFY--------------------GLVDLLAIVPGIIALYYSDA--QY----------------------------------------------------------------------------------------------------------------LLIIRVIRMLRIFRVLKLG-QYLNQA--------------------NYLMAALRG--------SKQKIIVFLLSV------STLVTVFGTLMYVI--EG-------------------------------------------------------------------------PEH-------------------------------------------------------------------------GFTSIPKGIYWAIVTLTTVGFGDIVPKTPLGQVLS-SLVMITGYSIIAVPTGIFTAELANAMRGE-------------------------------
23057785_1_250       -----------SNKHWREQLYVMI-------------FQSDTP----AGRRFDGILLLIILASLVIVMLDSIDSVHRN------------------------------------------------------------------------------------------------------------------------------------------------------------------------------------------------------------------------------------------------------------YADVLAYIEWGFTIIFAGEYILRLYCS-----------------PKPLRYAFSFY--------------------GLVDLLAIVPGILALYYSDA--QYLL----------------------------------------------------------------------------------------------------------------IIRIIRMLRIFRVLKLS-PYLKQA--------------------NYLMSALRG--------SKQKIVVFLVSV------CTLVTVFGTLMYVI--EG-------------------------------------------------------------------------PEH-------------------------------------------------------------------------GFTSIPKGIYWAIVTLTTVGFGDIVPKTPLGQVIS-SLVMITGYSIIAVPTGIFTAELANAMRG--------------------------------
28870782             -----------TSLSRRERLHIVI-------------FEANTP----AGRMFDKIVLVAILLSLLVTVIDSIESIHRDYA------------------------------------------------------------------------------------------------------------------------------------------------------------------------------------------------------------------------------------------------------------TLFAWIEWGFTLLFAIEYIMRLYCS-----------------PRPLKYAFSFY--------------------GLVDLLAIVPGVLSIYYSDA--QY----------------------------------------------------------------------------------------------------------------LLIVRIIRMLRIFRVLKLG-TYLRQA--------------------NYLLAALRG--------SKQKIIVFLVTV------STLVTVFGTLMYVI--EG-------------------------------------------------------------------------PEH-------------------------------------------------------------------------GFTSIPKGIYWAIVTLTTVGFGDIVPKTPVGQMLS-SLVMIIGYSIIAVPTGIFTAELANAMRGE-------------------------------
71736018             -----------TSLSRRERLHIVI-------------FEANTP----AGRTFDKIVLVAILLSLLVTVIDSIESIHRDYA------------------------------------------------------------------------------------------------------------------------------------------------------------------------------------------------------------------------------------------------------------MLFAWIEWGFTLLFAIEYILRLYCS-----------------PRPLKYAFSFY--------------------GLVDLLAIVPGVLSIYYSDA--QY----------------------------------------------------------------------------------------------------------------LLIVRIIRMLRIFRVLKLG-TYLRQA--------------------NYLLAALRG--------SKQKIIVFLVTV------STLVTVFGTLMYVI--EG-------------------------------------------------------------------------PEH-------------------------------------------------------------------------GFTSIPKGIYWAIVTLTTVGFGDIVPKTPVGQMLS-SLVMIIGYSIIAVPTGIFTAELANAMRGE-------------------------------
66046611             -----------SSLSRRERLHIVI-------------FEANTP----AGRMFDKAVLVAILLSLLVTIIDSIESIHRDYA------------------------------------------------------------------------------------------------------------------------------------------------------------------------------------------------------------------------------------------------------------MLFAWIEWGFTLMFAIEYMLRLYCS-----------------PRPLKYAFSFY--------------------GLVDLLAIVPGVLSIYYSDA--QY----------------------------------------------------------------------------------------------------------------LLIVRIIRMLRIFRVLKLG-TYLRQA--------------------NYLLAALRG--------SKQKIIVFLVTV------STLVTVFGTLMYVI--EG-------------------------------------------------------------------------PEH-------------------------------------------------------------------------GFTSIPKGIYWAIVTLTTVGFGDIVPKTPLGQMLS-SLVMITGYSIIAVPTGIFTAELANAMRGE-------------------------------
15596693             -----------APDSWRERLYIVI-------------FQTDTR----DGRRFDSALLLVILASLLVVMIDSIDEIHQDYG------------------------------------------------------------------------------------------------------------------------------------------------------------------------------------------------------------------------------------------------------------DLLAYIEWGFTGIFLVEYLLRLYCS-----------------PKPLRYAFSFY--------------------GLVDLLAILPGFLALLYPDA--QY----------------------------------------------------------------------------------------------------------------LLIVRVIRMLRIFRVLKLR-QYLSQA--------------------NFLLTALRG--------SKQKIIVFFLTV------MTLVTVFGALMYVV--EG-------------------------------------------------------------------------PEH-------------------------------------------------------------------------GFTSIPRGIYWAIVTLTTVGFGDITPKTPLGQAIA-SLVMLTGYSIIAVPTGIFTAELATAMRQDPAN----------------------------
94415057             -----------APDSWRERLYVII-------------FQTDTR----DGRRFDSALLLVILASLLVVMIDSIDEIHQDYG------------------------------------------------------------------------------------------------------------------------------------------------------------------------------------------------------------------------------------------------------------DLLAYIEWGFTGIFLVEYLLRLYCS-----------------PKPLRYAFSFY--------------------GLVDLLAILPGFLALLYPDA--QY----------------------------------------------------------------------------------------------------------------LLIVRVIRMLRIFRVLKLR-QYLSQA--------------------NFLLTALRG--------SKQKIIVFFLTV------MTLVTVFGALMYVV--EG-------------------------------------------------------------------------PEH-------------------------------------------------------------------------GFTSIPRGIYWAIVTLTTVGFGDITPKTPLGQAIA-SLVMITGYSIIAVPTGIFTAELATAMRQDPAS----------------------------
146307802            -----------KGQTWRQRLYIII-------------FETSTP----AAQRFDNWLLVTILASLVVVMLDSVEHFHNQYA------------------------------------------------------------------------------------------------------------------------------------------------------------------------------------------------------------------------------------------------------------EVFRALEWFFTVLFAIEYGLRLYIS-----------------PKPMRYAFSFF--------------------GLVDLLAVLPAILALMFADA--QY----------------------------------------------------------------------------------------------------------------LMIVRAIRLIRVFRVLKLR-QYLSQA--------------------NFLLVALRG--------SKQKIIVFLVSV------STLVTVYGALMYVI--EG-------------------------------------------------------------------------PAN-------------------------------------------------------------------------GFTSIPISIYWAVVTLTTVGFGDITPQTPIGQMLA-TLVMITGYSIIAVPTGIFTAELANAMRLEG------------------------------
67938293             -----------QQSTFRKKLQHII-------------FDFNTI----PAKLFDLVLIAAILLSVVVVMLDSVTRIHLDYG------------------------------------------------------------------------------------------------------------------------------------------------------------------------------------------------------------------------------------------------------------DVLYIMEWFFTLLFTLEYALRLYVA-----------------SSKTKYAFSFF--------------------GIIDLLSILPTYFSILFPGT--QY----------------------------------------------------------------------------------------------------------------LLVIRFFRVLRIFRLLKLG-KFVREG--------------------EFVKASLMA--------SSRKIVIFLFFV------LVTVSIIGALMYLI--EG-------------------------------------------------------------------------EEN-------------------------------------------------------------------------GFHSIPEGVYWAIVTITTVGYGDIYPQTIMGRMLA-AVLMITGYSIIAVPTGIVSAEMASMNEKIKSDLSG-------------------------
67918083             -----------AKRTFREVARHII-------------FDSSTI----PSKLFDLGLIGAICASVLVVMLDSVAPLHKLYG------------------------------------------------------------------------------------------------------------------------------------------------------------------------------------------------------------------------------------------------------------GPLYVLEWFFTVLFTLEYFLRLYAA-----------------KSRTRYATSFF--------------------GLIDLFSILPTYFSMLFPGA--QY----------------------------------------------------------------------------------------------------------------FLIVRFFRVLRIFRLLKLM-TFVREA--------------------DFVTASIRA--------SGRKIMVFLFFV------LVLVSIVGAIMYMI--EG-------------------------------------------------------------------------PEN-------------------------------------------------------------------------GFRSIPESIYWAIVTVTTVGYGDISPQTPAGRFLA-ALLMITGYSIIAVPTGIVSSEMSSMRGRNSRNRS--------------------------
110833952            ------------MENLRQRWQHII-------------FGTDTP----AGQLFDILLIVAILGSVVAVMLDSVASLHEQYG------------------------------------------------------------------------------------------------------------------------------------------------------------------------------------------------------------------------------------------------------------EALYAAEWFFTILFTIEYSVRLWVS-----------------ERPLRYARSFF--------------------GIVDLLSIIPTYLSLLVPGS--NY----------------------------------------------------------------------------------------------------------------LLTIRALRVLRVFRVMKLA-KFMGEA--------------------NQLGIALIR--------TRRKIAVFMFSV------FVVIIIFGSLMYVV--EG-------------------------------------------------------------------------PEH-------------------------------------------------------------------------GFTSIPQSIYWAIVTITTVGYGDISPQTGFGKMIA-SLAMITGYAIIAVPTGIVTAEISAARVSMRYERE--------------------------
84394609             -----------SRKPLKHHLYVII-------------FGTHTP----AGRAFDISLIVAILASLLVLILESIPNVMTEWS------------------------------------------------------------------------------------------------------------------------------------------------------------------------------------------------------------------------------------------------------------QQLRYIEYTFTALFTLEYLLRLYCS-----------------PKPKSYATSFY--------------------GVVDLLAILPTYLAIIFPGA--SF----------------------------------------------------------------------------------------------------------------MGVVRLLRVMRIFRILKLV-RYLQDS--------------------NILLRSLLM--------ARRKILIFFSTV------GILVVIFGALIFVI--EG-------------------------------------------------------------------------PEN-------------------------------------------------------------------------GFTSIPHSIYWAIVTITTVGYGDMIPQTALGKAIA-SLTMLLGYSILAVPTGIITAELSNEMNSHK------------------------------
145961395            -----------SRKPLKHHLYVII-------------FGTHTP----AGRAFDISLIVAILASLLVLILESIPNVMTEWS------------------------------------------------------------------------------------------------------------------------------------------------------------------------------------------------------------------------------------------------------------QELRYIEYSFTALFTVEYLLRLYCS-----------------PKPKSYATSFY--------------------GVVDLLAILPTYLAIIFPGA--SF----------------------------------------------------------------------------------------------------------------MGVVRLLRVMRIFRILKLV-RYLQDS--------------------NILLRSLLM--------ARRKILIFFSTV------GILVVIFGALIFVI--EG-------------------------------------------------------------------------PEN-------------------------------------------------------------------------GFTSIPHSIYWAIVTITTVGYGDMVPQTAIGKAIA-SLTMLLGYSILAVPTGIITAELSNEMNSHK------------------------------
90580102             -----------IKLTLRQRLYQII-------------FGTSTR----AGQLFDIGLIIAIVSSELILIISSVASIESKFS------------------------------------------------------------------------------------------------------------------------------------------------------------------------------------------------------------------------------------------------------------TALLGAEWLFTILFTIEYILRLYCS-----------------PRPLAYARSFY--------------------GIIDLIAIVPAYISFFVPGS--NY----------------------------------------------------------------------------------------------------------------LLIVRLIRVLRIFRVLKLA-RFLQDS--------------------NILLRSLRH--------AQRKIFVFFSSV------LILVTVFGSLMFLI--EG-------------------------------------------------------------------------PEN-------------------------------------------------------------------------GFTSIPKSIYWAVVTITTVGYGDIVPQTVMGKALA-SLTMLLGYSILAVPTGILTAELNQEMKTHR------------------------------
23026443_10_264      -----------KKLINREYLYEVI-------------FGTDTP----AGKGFDLVLIYMILLSVLVLMLDSVEAIRSQ------------------------------------------------------------------------------------------------------------------------------------------------------------------------------------------------------------------------------------------------------------FKWLLFGLEWMFTVIFTLEYLVRIYCT-----------------PNIRRYLTSFY--------------------GIIDLISVLPSYLSLFIPGA--NFLL----------------------------------------------------------------------------------------------------------------IVRLLRVLRVFRILKLV-RYLSEA--------------------NVLIRSMAM--------ARRKIFVFFITV------LVLSTVFGALMFVV--EG-------------------------------------------------------------------------PEH-------------------------------------------------------------------------GFTSIPRSIYWTIVTITTVGYGDITPQTTLGQVIA-SAAMLTGYSILAVPTGILTAELFQEMTR--------------------------------
91793727             -----------STADTRQHLRAII-------------FGTETR----WGRYFDISLIVCILLSVSLIFIDTIDSVHQEYG------------------------------------------------------------------------------------------------------------------------------------------------------------------------------------------------------------------------------------------------------------GLIRIGEWFFTVLFTLEYSLRLYCS-----------------ENRFSYAKSFY--------------------GLVDFISILPSYLGLFIPGA--NA----------------------------------------------------------------------------------------------------------------ALAIRVLRLFRIFRVLKLL-RYLAEG--------------------NLLLKAMMQ--------SSRKVFLFFFSV------SLIIMVLSVVMYVI--EG-------------------------------------------------------------------------PHN-------------------------------------------------------------------------GFTSIPKSMYWTVVTITTVGYGDITPQTPLGQGIA-ALTMLIGYSIIAIPTGILTAEISHEMVKMR------------------------------
114562766            -----------ADSPLKQQLRSVI-------------FGTETP----AGKRFDITLMVCIVLSVILIFIDTIEHINSRYG------------------------------------------------------------------------------------------------------------------------------------------------------------------------------------------------------------------------------------------------------------DYISIAEWTFTVFFTVEYILRLYCS-----------------LNRLHYARSFF--------------------GVVDLVSILPSYLGLIFPGA--NV----------------------------------------------------------------------------------------------------------------ALALRVLRLFRVFRVLKLL-RYLSDG--------------------NLLIKAMMQ--------SSRKVFIFFFSV------SLIIMVLSVVMYVV--EG-------------------------------------------------------------------------PNN-------------------------------------------------------------------------GFTSIPKSMYWTVVTITTVGYGDITPQTPLGQGIA-ALTMLIGYSIIAIPTGILTAEISHEMVRTR------------------------------
114047781            -----------TESPLKRQLRTII-------------FGTDTA----AGRYFDIGLMVCIVLSVALVFLDTVEAVHQDYG------------------------------------------------------------------------------------------------------------------------------------------------------------------------------------------------------------------------------------------------------------HIIRVLEWVFTLIFTIEYGLRLYCA-----------------THPVLYARSFY--------------------GLVDLLSVLPSYLALFIPGA--NF----------------------------------------------------------------------------------------------------------------TLVIRVLRLFRIFRVLKLL-RYLSEG--------------------NLLLKAMLQ--------SSRKVFLFFFSV------SLIIMVLSAVMYVV--EG-------------------------------------------------------------------------PEN-------------------------------------------------------------------------GFSSIPKSVYWTIVTITTVGYGDITPKTELGQAIA-AFTMLLGYSIIAIPTGILTAEISQEVGRHR------------------------------
117920862            -----------TESPLKHQLRTII-------------FGTDTP----AGRYFDIGLMVCIVLSVALVFLDTVEAVHQDYG------------------------------------------------------------------------------------------------------------------------------------------------------------------------------------------------------------------------------------------------------------HIIRVLEWVFTLIFTIEYGLRLYCA-----------------THPVLYARSFY--------------------GLVDLLSVLPSYLALFIPGA--NF----------------------------------------------------------------------------------------------------------------TLVIRVLRLFRIFRVLKLL-RYLSEG--------------------NLLLKAMLQ--------SSRKVFLFFFSV------SLIIMVLSAVMYVV--EG-------------------------------------------------------------------------PEN-------------------------------------------------------------------------GFSSIPKSVYWTIVTITTVGYGDITPKTELGQAIA-AFTMLLGYSIIAIPTGILTAEISQEVGRHR------------------------------
113970546            -----------TESPLKRQLRTII-------------FGTDTP----AGRYFDIGLMVCIVLSVALVFLDTVEAVHQDYG------------------------------------------------------------------------------------------------------------------------------------------------------------------------------------------------------------------------------------------------------------HIIRVLEWVFTLIFTIEYGLRLYCA-----------------THPVSYARSFY--------------------GLVDLLSVLPSYLALFIPGA--NF----------------------------------------------------------------------------------------------------------------TLVIRVLRLFRIFRVLKLL-RYLSEG--------------------NLLLKAMLQ--------SSRKVFLFFFSV------SLIIMVLSAVMYVV--EG-------------------------------------------------------------------------PEN-------------------------------------------------------------------------GFSSIPKSVYWTIVTITTVGYGDITPKTELGQAIA-AFTMLLGYSIIAIPTGILTAEISQEVGRHR------------------------------
113950647            -----------SESPLKRQLRTII-------------FGTDTP----AGRYFDIALMVCIVLSVGLVFLDTVEMFHREYG------------------------------------------------------------------------------------------------------------------------------------------------------------------------------------------------------------------------------------------------------------QIIRVLEWVFTVIFTIEYGLRLYCA-----------------THPVLYARSFY--------------------GVVDLLSVLPSYLALLIPGA--NF----------------------------------------------------------------------------------------------------------------TLVIRILRLFRIFRVLKLL-RYLSEG--------------------NVLLRAMMQ--------SSRKVFLFFFSV------SLIVMVLSAVMYVV--EG-------------------------------------------------------------------------PDN-------------------------------------------------------------------------GFSSIPKSVYWTIVTITTVGYGDITPKTGLGQAIA-AFTMLIGYSIIAIPTGILTAEISQEVGRHR------------------------------
118074699            -----------QEDTIKLKLRRVI-------------FGTDTR----LGRYFDISLIICIILSVTLVFLDTVASINSQYG------------------------------------------------------------------------------------------------------------------------------------------------------------------------------------------------------------------------------------------------------------QLISVLEWFFTIVFTIEYLLRLYCS-----------------AQPVQYVRSFY--------------------GVVDLLSILPSYLALLFPGA--NF----------------------------------------------------------------------------------------------------------------TLVIRILRLFRIFRVLKLL-RYLSEG--------------------NVLLRAMLQ--------SGRKVFIFFFSV------SLIVMVLSAIMYVV--EG-------------------------------------------------------------------------PGN-------------------------------------------------------------------------GFTSIPKSIYWTIVTITTVGYGDITPGTNLGQAIA-AFTMLLGYSIIAIPTGILTAEISQEMGKSK------------------------------
118756580            -----------PESSLKLKLRRVI-------------FGTDTP----AGRYFDISLMVCIVMSVLLVFLDTVASVHERYG------------------------------------------------------------------------------------------------------------------------------------------------------------------------------------------------------------------------------------------------------------DMIVIMEWGFTFIFTIEYILRLYCS-----------------AKPLSYARSFY--------------------GVVDLLSVLPTYLALFFPGA--SF----------------------------------------------------------------------------------------------------------------TMVIRILRLFRIFRVLKLL-RYLSEG--------------------NLLLRAMMQ--------SSRKVFIFFFSV------SLIVVVLSAVMYVV--EG-------------------------------------------------------------------------PNN-------------------------------------------------------------------------GFTSIPKSIYWTIVTITTVGYGDITPGTPLGQAIA-AFTMLLGYSIIAIPTGILTAEISQEIGRTR------------------------------
15805856_10_260      -----------HRPPWRRWLGNLI-------------FGLSTP----AARAYDKIVIVLIVASVLAVTLESVPELSHA------------------------------------------------------------------------------------------------------------------------------------------------------------------------------------------------------------------------------------------------------------VRARLRQTEWVFTVMFTADYLLRLLGA-----------------RRPLRYALSFY--------------------GLVDLLTILPSYLSLLFPGTQYLL------------------------------------------------------------------------------------------------------------------VVRALRLLRVFRVFKLA-RYSDQA--------------------ALIGEALQA--------SREKIIVFFISV------LSMVIVFGTLLYMV--EG-------------------------------------------------------------------------PES-------------------------------------------------------------------------GFTSIPTSIYWAVVTVTTVGYGDISPKTGLGKFIA-TLAMLSGYAIIAVPTGIVTVGLQQAQEA--------------------------------
86144204             -----------QKHTWKTKLHEVI-------------YEADTP----AGKSFDVVLLILIFLSIVLVMLESVAPLQAEYG------------------------------------------------------------------------------------------------------------------------------------------------------------------------------------------------------------------------------------------------------------DYFYLGEWIITIFFTVEYILRVISI-----------------KKPSSYIFSFY--------------------GLIDLISTIPLYLSFFIVGT--NA----------------------------------------------------------------------------------------------------------------LLTIRALRLLRVFRILKVT-RYIGEG--------------------NKLAKALKD--------SRPKILVFLFAV------MILSVIAGTLMYIV--EG-------------------------------------------------------------------------PEH-------------------------------------------------------------------------GFKSIPVSVYWCIVTLTTVGFGDIAPVTALGQFIA-TVIMVMGYGIIAVPTGIVSAEMAKGGQPEPEPNK--------------------------
89891602             -----------SSPVWKRKIHEII-------------YEADTP----AGKWFDVILLVLIVISLILVMLESIPGLGEKYA------------------------------------------------------------------------------------------------------------------------------------------------------------------------------------------------------------------------------------------------------------FEFITAEWIVTIFFSIEYILRIISI-----------------NKPTKYIFSFY--------------------GIIDFLSTIPLYLTFFFGGF--SA----------------------------------------------------------------------------------------------------------------LLAVRSLRLLRVFRILKIT-RYIGEA--------------------DKLSRAIKA--------SGPKIAVFLFAV------MVVSIIFGTLMYLV--EG-------------------------------------------------------------------------PEH-------------------------------------------------------------------------GFDSIPKSIYWCIVTLTTVGFGDIAPETPLGQFLA-TLIMIMGYGIIAVPTGIVSAEYAQDLKKNNETNRSKES----------------------
60682834             -----------HNQPLKHKLYVII-------------FESDTP----AGKAFDVTLIICILLSILLAIIESLQGLPSWLS------------------------------------------------------------------------------------------------------------------------------------------------------------------------------------------------------------------------------------------------------------TPFIVLEYLFTVFFTFEYVTRIYCS-----------------PNPRKYIFSFF--------------------GIVDLLATLPLYLAFFLPGA--RY----------------------------------------------------------------------------------------------------------------LLIIRAFRIIRVFRIFKLF-NFWLEG--------------------ERLLTSLRE--------SSKKIAVFFLFV------VILVVAIGTLMYMI--EG-----------------------------------------------------------------------TQPNT-------------------------------------------------------------------------QFNNIPNSIYWAIVTMTTVGYGDITPATALGKFLS-ACVMLIGYTIIAVPTGIVSASMMKEYKKLK------------------------------
29349894             -----------NDQKLKHKLYIII-------------FESDTP----SGKLFDVVLIGCILASVLLVIIESLKGLPSYLT------------------------------------------------------------------------------------------------------------------------------------------------------------------------------------------------------------------------------------------------------------TPFVVMEYLFTAFFTFEYLTRIYCS-----------------PRPKKYIFSFF--------------------GIVDLLATLPLYIGLIFPGA--RY----------------------------------------------------------------------------------------------------------------LLIIRAFRLIRVFRVFKLF-NFLNEG--------------------ERLLTALRE--------SSKKIAVFFLFV------VILVTSIGTLMYMI--EG-----------------------------------------------------------------------TLPNS-------------------------------------------------------------------------QFNNIPNSIYWAIVTMTTVGYGDITPVTGLGKFLS-ACVMLIGYTIIAVPTGIVSASMMKDYKRRR------------------------------
16765085             -----------SLSSVRRRLYHNL-------------FDLTTR----SGRRFEGLCALFALLSVLVIFVESGVGTEYHLTFDEW--------------------------------------------------------------------------------------------------------------------------------------------------------------------------------------------------------------------------------------------------------HIFVWLELCVTLIFTGEYLLRLFSW-----------------PEPAKYVFSFW--------------------GFIDLVTILPLYVMWLWPEISLNY----------------------------------------------------------------------------------------------------------------MFAWRAMRAIRVLRILKLL-RFMPSL--------------------RVFWSAIIS--------ARHQLILFYSFI------AIVMIIFGALMYLI--EG-------------------------------------------------------------------------PKY-------------------------------------------------------------------------GFTTLNASVYWAIVTVTTVGYGDITPHTPLGRIVA-SVLILIGYSVIAIPTGLITTHMSSAFQKRH------------------------------
16760140             -----------SLSSVRRRLYHNL-------------FDLTTR----SSRRFEGLCALFALLSVLVIFVESGVGTEYHLTFDEW--------------------------------------------------------------------------------------------------------------------------------------------------------------------------------------------------------------------------------------------------------HIFVWLELCVTLIFTGEYLLRLFSW-----------------PEPAKYVFSFW--------------------GFIDLVTILPLYVMWLWPEISLNY----------------------------------------------------------------------------------------------------------------MFAWRAMRAIRVLRILKLL-RFMPSL--------------------RVFWSAIIS--------ARHQLILFYSFI------AIVMIIFGALMYLI--EG-------------------------------------------------------------------------PKY-------------------------------------------------------------------------GFTTLNASVYWAIVTVTTVGYGDITPHTPLGRIVA-SVLILIGYSVIAIPTGLITTHMSSAFQKRH------------------------------
23132840_1_252       ---------------------TVL--------------EATTP----AGKLYNVLIFGAILLSVLALLLEPDPLGNSALRQT--------------------------------------------------------------------------------------------------------------------------------------------------------------------------------------------------------------------------------------------------------NVLWIDVAQNICLAVFAADFILHLLLV-----------------EQPRRYLFSFY--------------------GVIDASAVLFFFVPQIRSELLL----------------------------------------------------------------------------------------------------------------------WVFKFGRILRVFKLL-KFIDEA--------------------RVLGQALRG--------SARTICVYLFFV------FLLQVVLGYFIFVI--ES-----------------------------------------------------------------------GKPDS-------------------------------------------------------------------------QFQTVSNGVYWAIVTMTTVGYGDLVPQTALGRLLA-SVVMMLGFGIIAIPTGLLTVSGVRHQQK--------------------------------
23041655_27_276      ----------QCQSWQEN-IYYIF--------------EYSNP-KNFLSLLDDLIITILTSISVIAFILDTSPK-----------------------------------------------------------------------------------------------------------------------------------------------------------------------------------------------------------------------FSQH-------------------------------------YPEQLKLIEILVTIFFTIEYLIRIWIC-----------------TVNPKYNHPIYG--RLRYVITPL--------ALIDIVAILPFYLQVLFPS-LEFI--------------------------------------------------------------------------------------------------------------------KFTLLLRLFRLFKIS-RYSESL--------------------RTIFKVIAA--------KKEELIATLLVV------IFVLVFVSSLMFLV--ESEV------------------------------------------------------------------------QPN------------------------------------------------------------------------KFSSILETMWWGVVTLTTVGYGDVYPVTHLGKLLS-ATVAFLGIGIFALPAGIIASGFAEEVQK--------------------------------
23001380_131_268     -----------FSILEKD--PKTS--------------EFGW-----IAMIVNFSIVALVFSNIFAVIIQTEKS----------------------------------------------------------------------------------------------------------------------------------------------------------------------------------------------------------------------IATKYDT--------------------------------------FFSLLETVSVVFFTIEYLLRVWIS-----------------NLHKRFSHVVWG--RFKYTLTPI--------AIVDLLAILPFYLNLLFPIDFRFI--------------------------------------------------------------------------------------------------------------------TILRFFRLLRIFKLR-RYSQAL--------------------NTLWKVIVD--------KKEELVITFVTI------LGLLIVSASLMYYI--ERTS------------------------------------------------------------------------QPE------------------------------------------------------------------------LFSSIPATMWWSVSTLTTVGYGDIVPITSFGKLLS-ACIAMLGIAMFALPAALLSSGFTEQIKKQRIKNTIHTCPHCQGKIN--------------
46201458_59_312      -------------TLRRT-VYHMLGGE-----------GHSDL----LVKVVDAFLIALIVLNVAAVVLESIEH-----------------------------------------------------------------------------------------------------------------------------------------------------------------------------------------------------------------------LSIA-------------------------------------HGPVFHVFDLASVAIFSLEYALRLWTA---------------VELDDSRFHHPVWG--RLRWAVSPM--------AIVDLLSVLPFFLGIVVEIDL-----------------------------------------------------------------------------------------------------------------------RAIRVLRVLRVFKLG-RYSMAM--------------------NVMGAVARQ--------EARSIGAVLFVM------MVILVLTSSLIYLF--EHRA------------------------------------------------------------------------QPH------------------------------------------------------------------------VFSDIPTSMWWAVVTLTTLGYGDMVPITPLGRMLG-ALTAILGVGMIALPAGVLASGFSEQMRI--------------------------------
22961412_12_265      --------------RARHYFYEILESQ-----AQTSR----------MGVIVNRFIVFFIVLSVGITVMESVPA-----------------------------------------------------------------------------------------------------------------------------------------------------------------------------------------------------------------------MRED-------------------------------------YGRLFQALELLCLVMFSIEYYIRIWIA-----------------PEHLPYHHMSPLRAAWAYMISPQ--------GIVDCISVMPLWIALFGPDDL-----------------------------------------------------------------------------------------------------------------------RVLIILRMLRLLKFA-RYSSGM--------------------RSLLEVLES--------ERRALMACLVIL------ACATLVSATAMHIA--EGHV------------------------------------------------------------------------QPE------------------------------------------------------------------------KFGTIPDAMWWAIVTLSTIGYGDVVPATGIGRMVA-SATIICGLIMIALPVGIVANAFSEVIHR--------------------------------
6644152_160_426      --------------SFRQTMWRAFENP-----------HTSTL----ALVFYY-VTGFFIAVSVITNVVETVPC-GTVPG----------------------------------------------------------------------------------------------------------------------------------------------------------------------------------------------------------------SKELPCG---------------------------------ERYSVAFFCLDTACVMIFTVEYLLRLFAA-----------------PSRYRFIRSVM--------------------SIIDVVAIMPYYIGLVMTN-NEDVSG----------------------------------------------------------------------------------------------------------------AFVTLRVFRVFRIFKFS-RHSQGL--------------------RILGYTLKS--------CASELGFLLFSL------TMAIIIFATVMFYA--EK-------------------------------------------------------------------------GSS-----------------------------------------------------------------------ASKFTSIPASFWYTIVTMTTLGYGDMVPKTIAGKIFG-SICSLSGVLVIALPVPVIVSNFSRIYHQ--------------------------------
12751419_160_426     --------------SFRQTMWRAFENP-----------HTSTL----ALVFYY-VTGFFIAVSVITNVVETVPC-GTVPG----------------------------------------------------------------------------------------------------------------------------------------------------------------------------------------------------------------SKELPCG---------------------------------ERYSVAFFCLDTACVMIFTVEYLLRLFAA-----------------PSRYRFIRSVM--------------------SIIDVVAIMPYYIGLVMTN-NEDVSG----------------------------------------------------------------------------------------------------------------AFVTLRVFRVFRIFKFS-RHSQGL--------------------RILGYTLKS--------CASELGFLLFSL------TMAIIIFATVMFYA--EK-------------------------------------------------------------------------GSS-----------------------------------------------------------------------ASKFTSIPASFWYTIVTMTTLGYGDMVPKTIAGKIFG-SICSLSGVLVIALPVPVIVTNFSRIYHQ--------------------------------
6007797_160_426      --------------SFRQTMWRAFENP-----------HTSTL----ALVFYY-VTGFFIAVSVITNVVETVPC-GTVPG----------------------------------------------------------------------------------------------------------------------------------------------------------------------------------------------------------------SKELPCG---------------------------------ERYSVAFFCLDTACVMIFTVEYLLRLFAA-----------------PSRYRFIRSVM--------------------SIIDVVAIMPYYIGLVMTN-NEDVSG----------------------------------------------------------------------------------------------------------------AFVTLRVFRVFRIFKFS-RHSQGL--------------------RILGYTLKS--------CASELGFLLFSL------TMAIIIFATVMFYA--EK-------------------------------------------------------------------------GSS-----------------------------------------------------------------------ASKFTSIPASFWYTIVTMTTLGYGDMVPKTIAGKIFG-SICSLSGVLVIALPVPVIVSNFSGIYHQ--------------------------------
2935434_160_426      --------------SFRQTMWRAFENP-----------HTSTL----ALVFYY-VTGFFIAVSVITNVVETVPC-GTVPG----------------------------------------------------------------------------------------------------------------------------------------------------------------------------------------------------------------SKELPCG---------------------------------ERYSVAFFCLDTACVMIFTGEYLLRLFAA-----------------PSRYRFIRSVM--------------------SIIDVVAIMPYYIGLVMTN-NEDVSG----------------------------------------------------------------------------------------------------------------AFVTLRVFRVFRIFKFS-RHSQGL--------------------RILGYTLKS--------CASELGFLLFSL------TMAIIIFATVMFYA--EK-------------------------------------------------------------------------GSS-----------------------------------------------------------------------ASKFTSIPASFWYTIVTMTTLGYGDMVLKTIAGKIFG-SICSLSGVLVIALPVPVIVSNFSRIYHQ--------------------------------
6653655_161_427      --------------SFRQTMWRAFENP-----------HTSTL----ALVFYY-VTGFFIAVSVITNVVETVPC-GTVPG----------------------------------------------------------------------------------------------------------------------------------------------------------------------------------------------------------------NKELPCG---------------------------------ERYAVAFFCLDTACVMIFTVEYLLRLFAA-----------------PSRYRFIRSVM--------------------SIIDVVAIMPYYIGLVMTN-NEDVSG----------------------------------------------------------------------------------------------------------------AFVTLRVFRVFRIFKFS-RHSQGL--------------------RILGYTLKS--------CASELGFLLFSL------TMAIIIFATVMFYA--EK-------------------------------------------------------------------------GSS-----------------------------------------------------------------------ASKFTSIPASFWYTIVTMTTLGYGDMVPKTIAGKIFG-SICSLSGVLVIALPVPVIVSNFSRIYHQ--------------------------------
17981488_160_426     --------------SFRQTMWRAFENP-----------HTSTL----ALVFYY-VTGFFIAVSVITNVVETVPC-GTVPG----------------------------------------------------------------------------------------------------------------------------------------------------------------------------------------------------------------SKELPCG---------------------------------ERYSVAFFCLDTACVMIFTVEYLLRLFAA-----------------PSRYRFVRSVM--------------------SIIDVVAIMPYYIGLVMTN-NEDVSG----------------------------------------------------------------------------------------------------------------AFVTLRVFRVFRIFKFS-RHSQGL--------------------RILGYTLKS--------CASELGFLLFSL------TMAIIIFATVVFYA--EK-------------------------------------------------------------------------GSS-----------------------------------------------------------------------ASKFTSIPASFWYTIVTMTTLGYGDMVPKTIAGKIFG-SICSLSGVLVIALPVPVIVSNFSRIYHQ--------------------------------
2738111_160_431      --------------NLRETMWRAFENP-----------HTSTL----ALVFYY-VTGFFIAVSVIANVVETVPC-GTVPG----------------------------------------------------------------------------------------------------------------------------------------------------------------------------------------------------------------NKELPCG---------------------------------ERYQVAFFCLDTACVMIFTVEYLLRLFAA-----------------PSRYRFMRSVM-----------------SIDRSVMVVAIMPYYIGLVMTNINEDVS----------------------------------------------------------------------------------------------------------------GAFVTLRVFRVFRIFKFS-RHSQGL--------------------RILGYTLKS--------CASELGFLLFSL------TMAIIIFATVMFYA--EK-------------------------------------------------------------------------GST-----------------------------------------------------------------------SSKFTSIPASFWYTIVTMTTLGYGDMVPKTIAGKIFEFHLTSLSGVLVIALPDPVMVSNFSRIYHQ--------------------------------
21314406_162_429     --------------TARQRVWRAFENP-----------HTSTM----ALVFYY-VTGFFIAVSVIANVVETVPC-GSSPGH---------------------------------------------------------------------------------------------------------------------------------------------------------------------------------------------------------------IKELPCG---------------------------------ERYAVAFFCLDTACVMIFTVEYLLRLAAA-----------------PSRYRFVRSVM--------------------SIIDVVAILPYYIGLVMTD-NEDVSG----------------------------------------------------------------------------------------------------------------AFVTLRVFRVFRIFKFS-RHSQGL--------------------RILGYTLKS--------CASELGFLLFSL------TMAIIIFATVMFYA--EK-------------------------------------------------------------------------GSS-----------------------------------------------------------------------ASKFTSIPAAFWYTIVTMTTLGYGDMVPKTIAGKIFG-SICSLSGVLVIALPVPVIVSNFSRIYHQ--------------------------------
21311759_148_415     --------------TARQRVWRAFENP-----------HTSTM----ALVFYY-VTGFFIAVSVIANVVETVPC-GSSPGH---------------------------------------------------------------------------------------------------------------------------------------------------------------------------------------------------------------IKELPCG---------------------------------ERYAVAFFCLDTACVMIFTVEYLLRLAAA-----------------PSRYRFVRSVM--------------------SIIDVVAILPYYIGLVMTD-NEDVSG----------------------------------------------------------------------------------------------------------------AFVTLRVFRVFRIFKFS-RHSQGL--------------------RILGYTLKS--------CASELGFLLFSL------TMAIIIFATVMFYA--EK-------------------------------------------------------------------------GSS-----------------------------------------------------------------------ASKFTSIPAAFWYTIVTMTTLGYGDMVPRTIAGKIFG-SICSLSGVLVIALPVPVIVSNFSRIYHQ--------------------------------
7648673_162_429      --------------TARQRVWRAFENP-----------HTSTM----ALVFYY-VTGFFIAVSVIANVVETVPC-GSSPGH---------------------------------------------------------------------------------------------------------------------------------------------------------------------------------------------------------------IKELPCG---------------------------------XRYAVAFFCLDTACVMIFTVEYLLRLAAA-----------------PSRYRFVRSVM--------------------SIIDVVAILPYYIGLVMTD-NEDVSG----------------------------------------------------------------------------------------------------------------AFVTLRVFRVFRIFKFS-RHSQGL--------------------RILGYTLKS--------XASELGFLLFSL------TMAIIIFATVMFYA--EK-------------------------------------------------------------------------GSS-----------------------------------------------------------------------ASKFTSIPAAFWYTIVTMTTLGYGDMVPKTIAGKIFG-SICSLSGVLVIALPVPVIVSNFSRIYHQ--------------------------------
18032120_162_429     --------------TARQRMWRAFENP-----------HTSTL----ALVFYY-VTGFFIAVSVIANVVETVPC-GVSPGR---------------------------------------------------------------------------------------------------------------------------------------------------------------------------------------------------------------IKELPCG---------------------------------ERYAVAFFCLDTACVMIFTVEYLLRLLAA-----------------PSRYKFVRSVM--------------------SIIDVVAIMPYYIGLVMTD-NEDVSG----------------------------------------------------------------------------------------------------------------AFVTLRVFRVFRIFKFS-RHSQGL--------------------RILGYTLKS--------CASELGFLLFSL------TMAIIIFATVMYYA--EK-------------------------------------------------------------------------GSS-----------------------------------------------------------------------ASKFTSIPAAFWYTIVTMTTLGYGDMVPKTIAGKIFG-SICSLSGVLVIALPVPVIVSNFSRIYHQ--------------------------------
2143920_1_236        --------------------WRAFENP-----------HTSTL----ALVFYY-VTGFFIAVSVITNVVETVPC-GQVPG----------------------------------------------------------------------------------------------------------------------------------------------------------------------------------------------------------------RQGTAVW---------------------------------REYSVVFFCLDTACVMIFTVEYLLRLFQH-----------------PAGYRFIRSVM--------------------SIIDVVAIMPYYIGLVMTN-NEDVSG----------------------------------------------------------------------------------------------------------------AFVTLRVFRVFRIFKFS-RHSQGL--------------------RILGYTLKS--------CASELGFLLFSL------TMAIIIFATVMFYA--EK-------------------------------------------------------------------------SSS-----------------------------------------------------------------------ASKFTSIPASFWYTIVTMTTLGYGDMVPKTIAGKIFG-SICSLSGVLVIALPVPVIVSP---------------------------------------
27436981_163_431     --------------SLRQRLWRAFENP-----------HTSTA----ALVFYY-VTGFFIAVSVIANVVETIPCRGSARRS---------------------------------------------------------------------------------------------------------------------------------------------------------------------------------------------------------------SREQPCG---------------------------------ERFPQAFFCMDTACVLIFTGEYLLRLFAA-----------------PSRCRFLRSVM--------------------SLIDVVAILPYYIGLLVPK-NDDVSG----------------------------------------------------------------------------------------------------------------AFVTLRVFRVFRIFKFS-RHSQGL--------------------RILGYTLKS--------CASELGFLLFSL------TMAIIIFATVMFYA--EK-------------------------------------------------------------------------GTN-----------------------------------------------------------------------KTNFTNIPAAFWYTIVTMTTLGYGDMVPSTIAGKIFG-SICSLSGVLVIALPVPIIVSNFSRIYHQ--------------------------------
6680526_163_431      --------------SLRQRLWRAFENP-----------HTSTA----ALVFYY-VTGFFIAVSVIANVVETIPCRGTPRWP---------------------------------------------------------------------------------------------------------------------------------------------------------------------------------------------------------------SKEQSCG---------------------------------DRFPTAFFCMDTACVLIFTGEYLLRLFAA-----------------PSRCRFLRSVM--------------------SLIDVVAILPYYIGLFVPK-NDDVSG----------------------------------------------------------------------------------------------------------------AFVTLRVFRVFRIFKFS-RHSQGL--------------------RILGYTLKS--------CASELGFLLFSL------TMAIIIFATVMFYA--EK-------------------------------------------------------------------------GTS-----------------------------------------------------------------------KTNFTSIPAAFWYTIVTMTTLGYGDMVPSTIAGKIFG-SICSLSGVLVIALPVPVIVSNFSRIYHQ--------------------------------
17737641_162_429     --------------NMRQKMWRAFENP-----------HTSTS----ALVFYY-VTGFFIAVSVMANVVETVPC-GHRPGR---------------------------------------------------------------------------------------------------------------------------------------------------------------------------------------------------------------AGTLPCG---------------------------------ERYKIVFFCLDTACVMIFTAEYLLRLFAA-----------------PDRCKFVRSVM--------------------SIIDVVAIMPYYIGLGITD-NDDVSG----------------------------------------------------------------------------------------------------------------AFVTLRVFRVFRIFKFS-RHSQGL--------------------RILGYTLKS--------CASELGFLVFSL------AMAIIIFATVMFYA--EK-------------------------------------------------------------------------NVN-----------------------------------------------------------------------GTNFTSIPAAFWYTIVTMTTLGYGDMVPETIAGKIVG-GVCSLSGVLVIALPVPVIVSNFSRIYHQ--------------------------------
21291851_162_429     --------------NIRQRMWRAFENP-----------HTSTA----ALVFYY-VTGFFIAVSVMANVVETVPC-GVRPGR---------------------------------------------------------------------------------------------------------------------------------------------------------------------------------------------------------------AGTLSCG---------------------------------ERYKIVFFCLDTACVMIFTAEYLLRLFAA-----------------PCRCKFVRSVM--------------------SIIDVVAILPYYIGLGITD-NDDVSG----------------------------------------------------------------------------------------------------------------AFVTLRVFRVFRIFKFS-RHSQGL--------------------RILGYTLKS--------CASELGFLVFSL------AMAIIIFATVMFYA--EK-------------------------------------------------------------------------NVD-----------------------------------------------------------------------GTNFTSIPAAFWYTIVTMTTLGYGDMVPETIAGKIVG-GVCSLSGVLVIALPVPVIVSNFSRIYHQ--------------------------------
14190047_162_429     --------------TIRERMWRAFENP-----------HTSTA----ALVFYY-VTGFFIAVSVMANVVETVPC-GRLPGK---------------------------------------------------------------------------------------------------------------------------------------------------------------------------------------------------------------KESQPCG---------------------------------ERYKIIFFCGDTACVMIFTAEYLLRMFAA-----------------PDRCKFVRSVM--------------------SIIDVVAILPYYIGLGITD-NDDVSG----------------------------------------------------------------------------------------------------------------AFVTLRVFRVFRIFKFS-RHSQGL--------------------RILGYTLKS--------CASELGFLVFSL------AMAIIIFATVMFYA--EK-------------------------------------------------------------------------NVE-----------------------------------------------------------------------DTTFTSIPSAFWYTIVTMTTLGYGDMVPHTPTGKIVG-GVCSLSGVLVIALPVPVIVSNFSRIYHQ--------------------------------
17544122_165_430     --------------SLKEKMWAAFENP-----------HTTSI----ALVFYY-VIGFFIAVSVMCNIVETIPC-GYEDN-----------------------------------------------------------------------------------------------------------------------------------------------------------------------------------------------------------------VSVTCG---------------------------------EAYEEQFFVIDTACVIIFTIEYFLRLISA-----------------PDRIKFMRSIM--------------------SVIDVIAIMPYYVSLVLTD-NKDVSG----------------------------------------------------------------------------------------------------------------LFVTLRVFRVFRIFKFS-RHSQGL--------------------RILGYTLKS--------CASELGFLVFSL------AMAIIIFATIMYYA--EK-------------------------------------------------------------------------KVD-----------------------------------------------------------------------ATRFTSIPSAFWYTIVTLTTLGYGDMVPSTIMGKIVG-GVCSLSGVLVIALPVPVIVSNFSRIYHQ--------------------------------
1763619_179_439      --------------NFRQKVHGLFENP-----------QSTFL----ARILYY-ITGFFIAVSVGSTIIETIDC----------------------------------------------------------------------------------------------------------------------------------------------------------------------------------------------------------------------SANRPCG---------------------------------EVYNKIFFNIEAVCVVVFTIEYLARLYSA-----------------PCRFRHARISL--------------------SIIDVIAILPFYIGLAMTK--TSISGA----------------------------------------------------------------------------------------------------------------FVSLRVFRVFRIFKFS-RHSKGL--------------------RILGSTLTS--------CASELGFLLFSL------SMAIIIFATVVFYV--EK-------------------------------------------------------------------------DVN-----------------------------------------------------------------------DSDFTSIPASFWYTIVTMTTLGYGDMVPKTIPGKLVG-SICSLSGVLVIALPVPVIVSNFSRIYLQ--------------------------------
1763617_150_409      --------------TFREKCWIFCEDP------------TFSLA---AKCFYY-FSCLVITLSIVVNTVETVECRTSVNNT---------------------------------------------------------------------------------------------------------------------------------------------------------------------------------------------------------------AVFQRCS---------------------------------DAHPQIFFVLDSACVTFFALEYMFRFYST-----------------DNRYNYLKNVM--------------------SIIDILAILPFFVDLILFHLNVGTN--------------------------------------------------------------------------------------VVIS----------------------NILVAFRSVRIIRVFKLA-RHSQRL--------------------RVLSSSIYR--------STSELGFILFMY------INVVVMFATCIFYA--EN-------------------------------------------------------------------------SSAG---------------------------------------------------------------------MKSAFSSIPEAMWYTVVTTTTLGYGDVVPVTIQGKLIG-SMCCLMGVLVIALPVPIIQMKAS-------------------------------------
20827940_190_485     --------------RWQPRVWALFEDP-----------YSSRA----ARYVAF-ASLFFILISITTFCLETHEGFIHI----------------------------------SNK----------------------------------------------------------------------------TVTQAS-----------PIPG-------------------------------------------------------------------------A---PPENIT------------------------------NVEVETEPFLTYVEGVCVVWFTFEFLMRVTFC-----------------PDKVEFLKSSL--------------------NIIDCVAILPFYLEVGLSGLSSKA----------------------------------------------------------------------------------------AKD--------------------VLGFLRVVRFVRILRIFKLT-RHFVGL--------------------RVLGHTLRA--------STNEFLLLIIFL----ALG--VLIFATMIYYA--ERIG-----------------------------------------------------------------------ADPDDILG----------------------------SNHTY------------------------------------FKNIPIGFWWAVVTMTTLGYGDMYPKTWSGMLVG-ALCALAGVLTIAMPVPVIVNNFGMYYSL--------------------------------
3023499_267_562      --------------RWQPRVWALFEDP-----------YSSRA----ARYVAF-ASLFFILISITTFCLETHEGFIHI----------------------------------SNK----------------------------------------------------------------------------TVTQAS-----------PIPG-------------------------------------------------------------------------A---PPENIT------------------------------NVEVETEPFLTYVEGVCVVWFTFEFLMRVTFC-----------------PDKVEFLKSSL--------------------NIIDCVAILPFYLEVGLSGLSSKA----------------------------------------------------------------------------------------AKD--------------------VLGFLRVVRFVRILRIFKLT-RHFVGL--------------------RVLGHTLRA--------STNEFLLLIIFL----ALG--VLIFATMIYYA--ERIG-----------------------------------------------------------------------ADPDDILG----------------------------SNHTY------------------------------------FKNIPIGFWWAVVTMTTLGYGDMYPKTWSGMLVG-GLCALAGVLTIAMPVPVIVNNFGMYYSL--------------------------------
24497460_267_562     --------------RWQPRVWALFEDP-----------YSSRA----ARYVAF-ASLFFILISITTFCLETHEGFIHI----------------------------------SNK----------------------------------------------------------------------------TVTQAS-----------PIPG-------------------------------------------------------------------------A---PPENIT------------------------------NVEVETEPFLTYVEGVCVVWFTFEFLMRITFC-----------------PDKVEFLKSSL--------------------NIIDCVAILPFYLEVGLSGLSSKA----------------------------------------------------------------------------------------AKD--------------------VLGFLRVVRFVRILRIFKLT-RHFVGL--------------------RVLGHTLRA--------STNEFLLLIIFL----ALG--VLIFATMIYYA--ERIG-----------------------------------------------------------------------ADPDDILG----------------------------SNHTY------------------------------------FKNIPIGFWWAVVTMTTLGYGDMYPKTWSGMLVG-ALCALAGVLTIAMPVPVIVNNFGMYYSL--------------------------------
23320901_269_564     --------------RWQPRVWALFEDP-----------YSSRA----ARYVAF-ASLFFILISITTFCLETHEGFIHI----------------------------------SNK----------------------------------------------------------------------------TVTQAS-----------PIPG-------------------------------------------------------------------------A---PPENVT------------------------------NVEVETEPFLTYVEGVCVVWFTFEFLMRVTFC-----------------PDKGEFLKSSL--------------------NIIDCVAILPFYLEVALSGLSSKA----------------------------------------------------------------------------------------AKD--------------------VLGFLRVVRFVRILRIFKLT-RHFVGL--------------------RVLGHTLRA--------STNEFLLLIIFL----ALG--VLIFATMIYYA--ERIG-----------------------------------------------------------------------ADPDDILG----------------------------SNHTY------------------------------------FKNIPIGFWWAVVTMTTLGYGDMYPKTWSGMLVG-ALCALAGVLTIAMPVPVIVNNFGMYYSL--------------------------------
109125708            --------------QTPEPALCLPEPRTAQG------AGTARS----EAYVAF-ASLFFILISITTFCLETHEGFIHI----------------------------------SNK----------------------------------------------------------------------------TVTQAS-----------PIPG-------------------------------------------------------------------------A---PPENITN------------------------------VEVETEPFLTYVEGVCVVWFTFEFLMRITFC-----------------PDKVEFLKSSL--------------------NIIDCVAILPFYLEVGLSGLSSK----------------------------------------------------------------------------------------AAKD--------------------VLGFLRVVRFVRILRIFKLT-RHFVGL--------------------RVLGHTLRA--------STNEFLLLIIFL----ALG--VLIFATMIYYA--ERIG-----------------------------------------------------------------------ADPDDILG----------------------------SNHTY------------------------------------FKNIPIGFWWAVVTMTTLGYGDMYPKTWSKKLVG-ALCALAGVLTIAMPVPVIVNNFGMYYSLAMAKQKLPKKKNKHIP----------------
5817540_148_440      --------------RLRPKVWALFEDP-----------YSSKY----ARYIAL-ASLFFILISITTFCLETHEAFNDV----------------------------------NNK----------------------------------------------------------------------------TEVFT--------------QGN----------------------------------------------------------------------------ITKTET------------------------------ILEMETAPFLNYVEGICVIWFTFEFLIRVIFC-----------------PDKMEFIKSSL--------------------NIIDFVAILPFYLEIGLSGLSSKA----------------------------------------------------------------------------------------AKD--------------------VLGFLRVVRFVRILRIFKLT-RHFVGL--------------------RVLGHTLRA--------STNEFLLLIIFL----ALG--VLIFATMIYYA--ERIG-----------------------------------------------------------------------ADPDDITG----------------------------SKHTY------------------------------------FKNIPIGFWWAVVTMTTLGYGDMYPMTWSGMLVG-ALCALAGVLTIAMPVPVIVNNFGMYYSL--------------------------------
6981122_167_459      --------------RWQPRIWALFEDP-----------YSSRY----ARYVAF-ASLFFILVSITTFCLETHERFN----------------------------------PIVNK----------------------------------------------------------------------------TEIEN-------------------------------------------------------------------------------------------VRNGTQVRY-----------------------------YREAETEAFLTYIEGVCVVWFTFEFLMRVVFC-----------------PNKVEFIKNSL--------------------NIIDFVAILPFYLEVGLSGLSSKA----------------------------------------------------------------------------------------AKD--------------------VLGFLRVVRFVRILRIFKLT-RHFVGL--------------------RVLGHTLRA--------STNEFLLLIIFL----ALG--VLIFATMIYYA--ERIG-----------------------------------------------------------------------AQPNDPSA----------------------------SEHTH------------------------------------FKNIPIGFWWAVVTMTTLGYGDMYPQTWSGMLVG-ALCALAGVLTIAMPVPVIVNNFGMYYSL--------------------------------
4826786_167_459      --------------RWQPRIWALFEDP-----------YSSRY----ARYVAF-ASLFFILVSITTFCLETHERFN----------------------------------PIVNK----------------------------------------------------------------------------TEIEN-------------------------------------------------------------------------------------------VRNGTQVRY-----------------------------YREAETEAFLTYIEGVCVVWFTFEFLMRVIFC-----------------PNKVEFIKNSL--------------------NIIDFVAILPFYLEVGLSGLSSKA----------------------------------------------------------------------------------------AKD--------------------VLGFLRVVRFVRILRIFKLT-RHFVGL--------------------RVLGHTLRA--------STNEFLLLIIFL----ALG--VLIFATMIYYA--ERIG-----------------------------------------------------------------------AQPNDPSA----------------------------SEHTH------------------------------------FKNIPIGFWWAVVTMTTLGYGDMYPQTWSGMLVG-ALCALAGVLTIAMPVPVIVNNFGMYYSL--------------------------------
4960161_167_459      --------------RWQPRIWALFEDP-----------YSSRY----ARYVAF-ASLFFILVSITTFCLETHERFN----------------------------------PIVNK----------------------------------------------------------------------------TEIEN-------------------------------------------------------------------------------------------VRNDTQVRY-----------------------------YREAETEAFLTYIEGVCVVWFTFEFLMRVVFC-----------------PNKIEFIKNSL--------------------NIIDFVAILPFYLEAGLSGLSSKA----------------------------------------------------------------------------------------AKD--------------------VLGFLRVVRFVRILRIFKLT-RHFVGL--------------------RVLGHTLRA--------STNEFLLLIIFL----ALG--VLIFATMIYYA--ERIG-----------------------------------------------------------------------AQPNDPSA----------------------------SEHTH------------------------------------FKNIPIGFWWAVVTMTTLGYGDMYPQTWSGMLVG-ALCALAGVLTIAMPVPVIVNNFGMYYSL--------------------------------
6959886_156_446      --------------RWQRLLWALFEDP-----------YSSKY----ARWIAF-ASLFFILVSITTFCLETHEAFN----------------------------------PIINR----------------------------------------------------------------------------TEIDP-------------------------------------------------------------------------------------------LDNSTRV-------------------------------YQETETVVFLTYIEGVCVLWFTFEFIMRVTFC-----------------PSKLEFFRNTL--------------------NIIDFIAIMPFYLEVGLSGLSSKA----------------------------------------------------------------------------------------AKD--------------------VLGFLRVVRFVRILRIFKLT-RHFVGL--------------------RVLGHTLRA--------STNEFLLLIIFL----ALG--VLIFATMIYYA--ERIG-----------------------------------------------------------------------AKPNDPRA----------------------------SEHTH------------------------------------FKNIPIGFWWAVVTMTTLGYGDMYPQTWSGMLVG-ALCALAGVLTIAMPVPVIVNNFGMYYSL--------------------------------
21217563_206_496     --------------RLQPRMWALFEDP-----------YSSRA----ARFIAF-ASLFFILVSITTFCLETHEAFN----------------------------------IVKNK----------------------------------------------------------------------------TEPVI---------------------------------------------------------------------------------------------NGTSVVL-----------------------------QYEIETDPALTYVEGVCVVWFTFEFLVRIVFS-----------------PNKLEFIKNLL--------------------NIIDFVAILPFYLEVGLSGLSSKA----------------------------------------------------------------------------------------AKD--------------------VLGFLRVVRFVRILRIFKLT-RHFVGL--------------------RVLGHTLRA--------STNEFLLLIIFL----ALG--VLIFATMIYYA--ERVG-----------------------------------------------------------------------AQPNDPSA----------------------------SEHTQ------------------------------------FKNIPIGFWWAVVTMTTLGYGDMYPQTWSGMLVG-ALCALAGVLTIAMPVPVIVNNFGMYYSL--------------------------------
21217559_206_496     --------------KLQPRMWALFEDP-----------YSSRA----ARFIAF-ASLFFILVSITTFCLETHEAFN----------------------------------IVKNK----------------------------------------------------------------------------TEPVI---------------------------------------------------------------------------------------------NGTSAVL-----------------------------QYEIETDPALTYVEGVCVVWFTFEFLVRIVFS-----------------PNKLEFIKNLL--------------------NIIDFVAILPFYLEVGLSGLSSKA----------------------------------------------------------------------------------------AKD--------------------VLGFLRVVRFVRILRIFKLT-RHFVGL--------------------RVLGHTLRA--------STNEFLLLIIFL----ALG--VLIFATMIYYA--ERVG-----------------------------------------------------------------------AQPNDPSA----------------------------SEHTQ------------------------------------FKNIPIGFWWAVVTMTTLGYGDMYPQTWSGMLVG-ALCALAGVLTIAMPVPVIVNNFGMYYSL--------------------------------
73968773             --------------RLQPRVWALFEDP-----------YSSRA----ARFIAF-ASLFFILVSITTFCLETHEAFN----------------------------------IVKNK----------------------------------------------------------------------------TEPVING---------------------------------------------------------------------------------------------TSVVLQ-----------------------------YEIETDPALTYVEGVCVVWFTFEFLVRIIFS-----------------PNKLEFIKNLL--------------------NIIDFVAILPFYLEVGLSGLSSK----------------------------------------------------------------------------------------AAKD--------------------VLGFLRVVRFVRILRIFKLT-RHFVGL--------------------RVLGHTLRA--------STNEFLLLIIFL----ALG--VLIFATMIYYA--ERVG-----------------------------------------------------------------------AQPNDPSA----------------------------SEHTQ------------------------------------FKNIPIGFWWAVVTMTTLGYGDMYPQTWSGMLVG-ALCALAGVLTIAMPVPVIVNNFGMYYSLAMAKQKLPRKRKKHIP----------------
12313898_203_495     --------------GWQPRMWALFEDP-----------YSSRA----ARVVAF-ASLFFILVSITTFCLETHEAFN----------------------------------IDRNV----------------------------------------------------------------------------TEILR--------------VGN-----------------------------------------------------------------------------ITSVHF-----------------------------RREVETEPILTYIEGVCVLWFTLEFLVRIVCC-----------------PDTLDFVKNLL--------------------NIIDFVAILPFYLEVGLSGLSSKA----------------------------------------------------------------------------------------ARD--------------------VLGFLRVVRFVRILRIFKLT-RHFVGL--------------------RVLGHTLRA--------STNEFLLLIIFL----ALG--VLIFATMIYYA--ERIG-----------------------------------------------------------------------ARPSDPRG----------------------------NDHTD------------------------------------FKNIPIGFWWAVVTMTTLGYGDMYPKTWSGMLVG-ALCALAGVLTIAMPVPVIVNNFGMYYSL--------------------------------
24497462_203_495     --------------GWQPRMWALFEDP-----------YSSRA----ARVVAF-ASLFFILVSITTFCLETHEAFN----------------------------------IDRNV----------------------------------------------------------------------------TEILR--------------VGN-----------------------------------------------------------------------------ITSVHF-----------------------------RREVETEPILTYIEGVCVLWFTLEFLVRIVCC-----------------PDTLDFVKNLL--------------------NIIDFVAILPFYLEVGLSGLSSKA----------------------------------------------------------------------------------------ARD--------------------VLGFLRVVRIVRILRIFKLT-RHFVGL--------------------RVLGHTLRA--------STNEFLLLIIFL----ALG--VLIFATMIYYA--ERIG-----------------------------------------------------------------------ARPSDPRG----------------------------NDHTD------------------------------------FKNIPIGFWWAVVTMTTLGYGDMYPKTWSGMLVG-ALCALAGVLTIAMPVPVIVNNFGMYYSL--------------------------------
20875213_207_499     --------------GWQPRMWALFEDP-----------YSSRA----ARVVAF-ASLFFILVSITTFCLETHEAFN----------------------------------IDRNV----------------------------------------------------------------------------TEIHR--------------VGN-----------------------------------------------------------------------------ITSVRF-----------------------------RREVETEPILTYIEGVCVMWFTLEFLVRIVCC-----------------PDTLDFVKNLL--------------------NIIDFVAILPFYLEVGLSGLSSKA----------------------------------------------------------------------------------------ARD--------------------VLGFLRVVRFVRILRIFKLT-RHFVGL--------------------RVLGHTLRA--------STNEFLLLIIFL----ALG--VLIFATMIYYA--ERIG-----------------------------------------------------------------------ARPSDPRG----------------------------NDHTD------------------------------------FKNIPIGFWWAVVTMTTLGYGDMYPKTWSGMLVG-ALCALAGVLTIAMPVPVIVNNFGMYYSL--------------------------------
21311755_29_321      --------------GWQPRMWALFEDP-----------YSSRA----ARVVAF-ASLFFILVSITTFCLETHEAFN----------------------------------IDRNV----------------------------------------------------------------------------TEIHR--------------VGN-----------------------------------------------------------------------------ITSVRF-----------------------------RREVETEPILTYIEGVCVLWFTLEFLVRIVCC-----------------PDTLDFVKNLL--------------------NIIDFVAILPFYLEVGLSGLSSKA----------------------------------------------------------------------------------------ARD--------------------VLGFLRVVRFVRILRIFKLT-RHFVGL--------------------RVLGHTLRA--------STNEFLLLIIFL----ALG--VLIFATMIYYA--ERIG-----------------------------------------------------------------------ARPSDPRG----------------------------NDHTD------------------------------------FKSIPIGFWWAVVTMTTLGYGDMYPKTWSGMLVG-ALCALAGVLTIAMPVPVIVNNFGMYYSL--------------------------------
109467415            --------------SLFPVRPTVADVTA----------ISYSG----PQVVAF-ASLFFILVSITTFCLETHEAFN----------------------------------IDRNV----------------------------------------------------------------------------TEIHR--------------VGN--------------------------------------------------------------------------I---TSVRFR-----------------------------REVETEPILTYIEGVCVMWFTLEFLVRIVCC-----------------PDTLDFVKNLL--------------------NIIDFVAILPFYLEVGLSGLSSK----------------------------------------------------------------------------------------AARD--------------------VLGFLRVVRFVRILRIFKLT-RHFVGL--------------------RVLGHTLRA--------STNEFLLLIIFL----ALG--VLIFATMIYYA--ERIG-----------------------------------------------------------------------ARPSDPRG----------------------------NDHTD------------------------------------FKNIPIGFWWAVVTMTTLGYGDMYPKTWSGMLVG-ALCALAGVLTIAMPVPVIVNNFGMYYSLAMAKQKLPKKRKKHVP----------------
125888610            ------------------LVRLENDQP-----------NPAKT----SQYVAF-GSLLFILISISTFCMETHEAFN----------------------------------TIYNK----------------------------------------------------------------------------TENVT--------------EGN------------------------------------------------------------------------V---TREEIV-------------------------------YEVVTDSWLTYVEGVCVVWFTIEVFTRVVFC-----------------PDKMEFFKSPL--------------------NIIDFVAVLPFYLEVGLSGLSSK----------------------------------------------------------------------------------------AAKD--------------------VLGFLRVVRFVRILRIFKLT-RHFVGL--------------------RVLGHTLRA--------STNEFLLLIIFL----ALG--VLIFATMIYYA--ERIG-----------------------------------------------------------------------ASPDDPTAS----------------------------AHTN------------------------------------FKNIPIGFWWAVVTMTTLGYGDMYPETWSGMLVG-ALCALAGVLTIAMPVPVIVNNFGMYYSLAMAKQKLPKKKNKHIP----------------
11878237_186_479     --------------VWQPRIWALFEDP-----------YSSKY----ARYVAF-GSLLFILISISTFCMETHEAFN----------------------------------TIYNK----------------------------------------------------------------------------TENIT--------------VGN---------------------------------------------------------------------------VTREEI-------------------------------VYEVVTDSWLTYVEGVCVIWFTIEVFMRVTFC-----------------PDKAEFFKSLL--------------------NIIDFVAILPFYLEVALSGLSSKA----------------------------------------------------------------------------------------AKD--------------------VLGFLRVVRFVRILRIFKLT-RHFVGL--------------------RVLGHTLRA--------STNEFLLLIIFL----ALG--VLIFATMIYYA--ERIA-----------------------------------------------------------------------GNPDDPHRP---------------------------ARHTN------------------------------------FKNIPIGFWWAVVTMTTLGYGDMYPETWSGMLVG-ALCALAGVLTIAMPVPVIVNNFGMYYSL--------------------------------
47208124             --------------VWQPNMWALFEDP-----------YSSKY----AQYVAF-GSLFFILLSISTFCLETHEVFN----------------------------------TIYNK----------------------------------------------------------------------------TENTT--------------TGN------------------------------------------------------------------------S---TREEVVCV----------------------------LFEVVTDEWLTYVEGVCVIWFTIEVLLRVIFC-----------------PDKAEFFRSPL--------------------NIIDFVAIVPFYLEVALSGLSSK----------------------------------------------------------------------------------------TAKD--------------------VLSFLRVVRFVRILRIFKLT-RHFVGL--------------------RVLGHTLRA--------STNEFLLLIIFL----ALG--VLIFATMIYYA--ERLG-----------------------------------------------------------------------ADQKDPTAA----------------------------AHTD------------------------------------FKNIPIGFWWAVVTMTTLGYGDMFPKTWSGMLVG-ALCALAGVLTIAMPVPVIVNNFGMYYSLAMAKQKLPKKRNKHIP----------------
17946111_151_436     --------------EMKPRIWSLFDEP-----------YSSNA----AKTIGV-VSVFFICISILSFCLKTHPDMRVP--------------------------------IVR------------------------------------------------------------------------------NITVKTAN----------------------------------------------------------------------------------------GSNGWFLDK------------------------------TQTNAHIAFFYIECVCNAWFTFEILVRFISS-----------------PNKWEFIKSSV--------------------NIIDYIATLSFYIDLVLQRFASHL---------------------------------------------------------------------------------------ENAD--------------------ILEFFSIIR---IMRLFKLT-RHSSGL--------------------KILIQTFRA--------SAKELTLLVFFL----VLG--IVIFASLVYYA--ERIQ-----------------------------------------------------------------------PNPH-----------------------------------------------------------------------NDFNSIPLGLWWALVTMTTVGYGDMAPKTYIGMFVG-ALCALAGVLTIALPVPVIVSNFAMYYSH--------------------------------
3219511_151_433      --------------RIQPKIWALFDEP-----------YSSTA----AKIIAV-ISVFFIVISILSFCLKTHPEMRVP--------------------------------VLK------------------------------------------------------------------------------NATSRTVN----------------------------------------------------------------------------------------GTYTWRLEK------------------------------KETEPHEAFFYIECASNAWFTFEIIIRFVVA-----------------PNKLDFLKAPV--------------------NIIDIVATLSFYLDFMLTELKQES------------------------------------------------------------------------------------------D--------------------MLEFFSIIR---IMRLCKLT-RHSAGL--------------------KILIHTFKA--------SAKELILLIFFL----VLG--IVIFAALIYYA--ERIQ-----------------------------------------------------------------------YNPE-----------------------------------------------------------------------NDFESIPVGLWWAIVTMTTVGYGDMAPKTYVGMFVG-ALCALAGVLCIALPVPVIVSNFALFYSH--------------------------------
45445057_153_573     --------------RIKPKIWAMFDEP-----------SSSTG----AKIVAG-MSVFFIFVSVISFCLKTHPGFRVD--------------------------------LPSGAHDAHGPGAGGPPHGHDPMGEPPQTHQYHQHSVTNYTSY--------SSGNFTASGQATPIATIKGGQRQRL---KRNINGSILNEFI--------------------------------------------------------------------------------EEKILGHNGRRKHG-WI--------------------------ETYGQPHEAFFYVELVCNVWFFIEVIIRLI------------------------FIKSPV--------------------NIIDFTATLSFYTDVMQRMGEYTG---------------------------------------------------------------------------------------------------------------LLEAFSIVR---IMRLFKLT-RHSPGL--------------------RILIHTFKA--------SAKELTLLVFFL----VLG--IVFFASLAYYA--EKLQ-----------------------------------------------------------------------DNPD-----------------------------------------------------------------------NQFKSIPLGLWWAIVTMTTVGYGDVAPKTYPGMFVG-ALCALAGVLTIALPVPVIVSNFSMFYSH--------------------------------
72000220_146_435     --------------KIKPRIWRLFDEP-----------NSSRS----AQFIAA-ISVFFLITAIIVFCLKTHPGLRIP--------------------------------ELAPFG--------------------------------------------------------------------NF---SRNHRSTSSR---------------------------------------------------------------------------------------IHPAQINIDK------------------------------ANSRPHPTFMYIETICNIWFTIEILARFSSC-----------------PSRFEYLRAPV--------------------NIIDIVATLTFYIDLLSSMFGATA---------------------------------------------------------------------------------------------------------------DLEFFSIIR---IMRLFKLT-HHNSGL--------------------KILMHTFRA--------SAKELMLLVFFL----VLG--VVVFASLVYYA--ERVE-----------------------------------------------------------------------SNED-----------------------------------------------------------------------NQFVSIPIGLWWAIVTMTTIGYGDITPHTYLGRLIG-SICALAGVLTIALPVPVIVSNFAMFYSH--------------------------------
2218158_179_463      --------------KLKPQMWSLFDEP-----------YSSQA----AKLIAG-ISVLFIFISIFSFCLKTHQSFRLP--------------------------------VLIG----------------------------------------------------------------------------QNITMPG------------------------------------------------------------------------------------------GVVQPSIER------------------------------VSTEPLPIFGQIEMLCNIWFTLELIIRFVFC-----------------PSKIRFFKSPL--------------------NMIDLVATLSFYADAMMVRVVEDE----------------------------------------------------------------------------------------PKD--------------------VVEFLSMIR---IFRLFKLT-QHHQGL--------------------QILIHTFRA--------SAKELILLVFFL----ILG--IVIFAALVYYA--EKME-----------------------------------------------------------------------ANPN-----------------------------------------------------------------------NQFQSIPLGLWWAICTMTTVGYGDMTPHTSFGRLVG-SLCAVMGVLTIALPVPVIVSNFAMFYSH--------------------------------
22832462_159_456     --------------EKQRKVWLLFEYP-----------ESSQA----ARVVAI-ISVFVILLSIVIFCLETLPEFKHY---------------------------------KVFN---------------------------------------------------------------------------TTTNGTK--------------------------------------------------------------------------------------------IEEDE---------------------------------VPDITDPFFLIETLCIIWFTFELTVRFLAC-----------------PNKLNFCRDVM--------------------NVIDIIAIIPYFITLATVVAEE------EDT-----------------LNLPKAPVSP-----------------------------------------------QDKSSNQAMS---------------------LAILRVIRLVRVFRIFKLS-RHSKGL--------------------QILGRTLKA--------SMRELGLLIFFL----FIG--VVLFSSAVYFA--EA-------------------------------------------------------------------------GSENS-----------------------------------------------------------------------FFKSIPDAFWWAVVTMTTVGYGDMTPVGFWGKIVG-SLCVIAGVLTIALPVPVIVSNFNYFYHR--------------------------------
45549143_204_501     --------------EKQRKVWLLFEYP-----------ESSQA----ARVVAI-ISVFVILLSIVIFCLETLPEFKHY---------------------------------KVFN---------------------------------------------------------------------------TTTNGTK--------------------------------------------------------------------------------------------IEEDE---------------------------------VPDITDPFFLIETLCIIWFTFELTVRFLAC-----------------PNKLNFCRDVM--------------------NVIDIIAIIPYFITLATVVAEE------EDT-----------------LNLPKAPVSP-----------------------------------------------QDKSSNQAMS---------------------LAILRVIRLVRVFRIFKLS-RHSKGL--------------------QILGRTLKA--------SMRELGLLIFFL----FIG--VVLFSSAVYFA--EA-------------------------------------------------------------------------GSENS-----------------------------------------------------------------------FFKSIPDAFWWAVVTMTTVGYGDMTPVGFWGKIVG-SLCVVAGVLTIALPVPVIVSNFNYFYHR--------------------------------
21392146_159_456     --------------EKQRKVWLLFEYP-----------ESSQA----ARVVAI-ISVFVILLSIVIFCLETLPEFKHY---------------------------------KVFN---------------------------------------------------------------------------TTTNGTK--------------------------------------------------------------------------------------------IEEDE---------------------------------VPDITDPFFLIETLCIIWFTFELTVRFLAC-----------------PNKLNFCRDVM--------------------NVIDIIAIIPYFITLATVVAEE------EDT-----------------LNLPKAPVSP-----------------------------------------------QDKSSNQAMS---------------------LAMLRVIRLVRVFRIFKLS-RHSKGL--------------------QILGRTLKA--------SMRELGLLIFFL----FIG--VVLFSSAVYFA--EA-------------------------------------------------------------------------GSENS-----------------------------------------------------------------------FFKSIPDAFWWAVVTMTTVGYGDMTPVGFWGKIVG-SLCVIAGVLTIALPVPVIVSNFNYFYHR--------------------------------
85111_192_489        --------------EKQRKVWLLFEYP-----------ESSQA----ARVVAI-ISVFVILLSIVIFCLETLPEFKHY---------------------------------KVFN---------------------------------------------------------------------------TTTNGTK--------------------------------------------------------------------------------------------IEEDE---------------------------------VPDITDPFFLIETLCIIWFTFELTVRFLAC-----------------PNKLNFCRDVM--------------------NVIDIIAIIPYFITLATVVAEE------EDT-----------------LNLPKAPVSP-----------------------------------------------QDKSSNQAMS---------------------LAILRVIRLVRVFRIFKLS-RHSKGL--------------------QILGRTLKA--------SMRELGLLIFFL----FIG--VVLFSSAVYFA--EA-------------------------------------------------------------------------GSENS-----------------------------------------------------------------------FFKSIPDAFWWAVVTMTTVGYGDMTPVGVWGKIVG-SLCAIAGVLTIALPVPVIVSNFNYFYHR--------------------------------
116130663_143_440    --------------ENQRKVWLLFEYP-----------ESSQA----ARVVAI-ISVFVILLSIVIFCLETLPEFKHY---------------------------------KVFN---------------------------------------------------------------------------TTTNGTK--------------------------------------------------------------------------------------------IEEDE---------------------------------VPDITDPFFLIETICIIWFTFELSVRFLAC-----------------PNKLNFFRDVM--------------------NIIDIIAIIPYFITLATVVAEE------EDT-----------------LNLPKAPVSP-----------------------------------------------QDKSTNQAMS---------------------LAILRVIRLVRVFRIFKLS-RHSKGL--------------------QILGRTLKA--------SMRELGLLIFFL----FIG--VVLFSSAVYFA--EA-------------------------------------------------------------------------GTEMS-----------------------------------------------------------------------FFKSIPDAFWWAVVTMTTVGYGDMRPVGVWGKIVG-SLCAIAGVLTIALPVPVIVSNFNYFYHR--------------------------------
119115080            --------------ENQRKVWLLFEYP-----------ESSQA----ARVVAI-ISVFVILLSIVIFCLETLPEFKHY---------------------------------KVFN---------------------------------------------------------------------------TTTNG--------------------------------------------------------------------------------------------TKIEEDE---------------------------------VPDITDPFFLIETICIIWFTFELSVRFLAC-----------------PNKLNFFRDVM--------------------NIIDIIAIIPYFITLATVVAEE------EDT-----------------LNLPKAPVSP-----------------------------------------------QDKSTNQAMS---------------------LAILRVIRLVRVFRIFKLS-RHSKGL--------------------QILGRTLKA--------SMRELGLLIFFL----FIG--VVLFSSAVYFA--EA-------------------------------------------------------------------------GSENSF-----------------------------------------------------------------------FKSIPDAFWWAVVTMTTVGYGDMTPVGVWGKIVG-SLCAIAGVLTIALPVPVIVSNFNYFYHRETDQEEMQSQNFNHVT----------------
2959684_161_459      --------------ENQRKVWLLFEYP-----------ESSQN----ARVVAI-ISVFVILLSIVIFCLETLPEFKHY---------------------------------KVFN---------------------------------------------------------------------------TTTNGTV--------------------------------------------------------------------------------------------VEEDE---------------------------------VPEVTDPFFLIETICIVWFSTELLVRFFAS-----------------PLKGAFLKDVM--------------------NIIDIVAIIPYFITLATVVAEE------ESE----------------ELVIPAETLSP-----------------------------------------------HEKGSNQAMS---------------------LAILRVIRLVRVFRIFKLS-RHSKGL--------------------QILGRTLKA--------SMRELGLLIFFL----FIG--VVLFSSAVYFA--EA-------------------------------------------------------------------------GSEKS-----------------------------------------------------------------------YFKSIPDAFWWAVVTMTTVGYGDMTPVGVWGKIVG-SLCAIAGVLTIALPVPVIVSNFNYFYHR--------------------------------
155764_173_470       --------------EFQRRVWLLFEYP-----------ESSAA----ARLCAI-FSVVIILLSIVIFCLETLPQFKHY---------------------------------RVVN---------------------------------------------------------------------------STANDSK------------------------------------------------------------------------------------------ESIEEDD---------------------------------IPKFNEPFFIIETCCIIWFTFELLVRFASC-----------------PEKLGFFKNIM--------------------NCIDIVAIIPYFITLGTVVADQ-----------------------------SKS-------------------------------------------------------NNQAMS---------------------LAILRVIRLVRVFRIFKLS-RHSKGL--------------------QILGQTLKA--------SMRELGLLIFFL----FIG--VILFSSAVYFA--EA-------------------------------------------------------------------------DADQT-----------------------------------------------------------------------HFKSIPDAFWWAVVTMTTVGYGDMRPIGVWGKLVG-SLCAIAGVLTIALPVPVIVSNFNYFYHR--------------------------------
510098_158_446       --------------RFQRKVWLLFEYP-----------ESSLA----ARILAI-GSVFVILLSIIIFCLETLPHFRRY---------------------------------KIINNLN------------------------------------------------------------------------STLCYEE------------------------------------------------------------------------------------------LTFEEDD---------------------------------LPTIDQPFFIIETFCIVWFSCELLVRFASS-----------------PKKFEFFKVLM--------------------NVIDVVSIIPYFITLGAVIIDD-----------------------------PKQ-------------------------------------------------------INQTTS---------------------LAVLRVIRLVRVFRIFKLS-RHSKGL--------------------QILGQTLRA--------SVRELGLLVFFL----LIC--VILFSSAVYFA--EA-------------------------------------------------------------------------DADTS-----------------------------------------------------------------------LFRSIPDGFWWAVVTMTTVGYGDMRPVTVWGKLIG-SLCAIAGVLTIALPVPVIVSNFNYFYHR--------------------------------
1245451_147_427      --------------EFQRRVWLLVEHP-----------ESSTP----ARVIAI-VSVSVIVISIVTFCLETLPEFKRY---------------------------------ELLN---------------------------------------------------------------------------DTI------------------------------------------------------------------------------------------------IVDDA---------------------------------MPKFGTPFFIIETGCIIWFTMELLIRFASC-----------------PGKLDFFKDSM--------------------NAIDVVSIMPYFITLGTVIQND-----------------------------TNT------------------------------------------------------QDKQNMS---------------------LAILRVIRLVRVFRIFKLS-RHSKGL--------------------QILGQTLKA--------SLRELGLLIFFL----VIG--VVLFSSAVYFA--EV-------------------------------------------------------------------------DTEQS-----------------------------------------------------------------------HFKSIPDSFWWAVVTMTTVGYGDMSPVGVYGKLVG-SLCAIAGVLTIALPVPVIVSNFNYFYHR--------------------------------
64427051_193_480     --------------DFHRQFWLLFEYP-----------ESSSA----ARGVAV-VSVLVVVISITIFCLETLPEFREDRE------------------------------LKVVRD--------------------------------------------------------------------------PSINTN-------------------------------------------------------------------------------------------KTGLSQT-----------------------------------MFTDPFFMVESTCIVWFTFELVLRFVVC-----------------PSKTDFFKNIM--------------------NIIDIISIIPYFATLITELVQE-----------------------------TEP------------------------------------------------------SAQQNMSL---------------------AILRIIRLVRVFRIFKLS-RHSKGL--------------------QILGQTLKA--------SMRELGLLIFFL----FIG--VILFSSAVYFA--EV-------------------------------------------------------------------------DEPES-----------------------------------------------------------------------HFSSIPDGFWWAVVTMTTVGYGDMCPTTPGGKIVG-TLCAIAGVLTIALPVPVIVSNFNYFYHR--------------------------------
5031819_193_480      --------------DIHRQFWLLFEYP-----------ESSSA----ARAVAV-VSVLVVVISITIFCLETLPEFREDRE------------------------------LKVVRD--------------------------------------------------------------------------PNLNMS-------------------------------------------------------------------------------------------KTVLSQT-----------------------------------MFTDPFFMVESTCIVWFTFELVLRFVVC-----------------PSKTDFFRNIM--------------------NIIDIISIIPYFATLITELVQE-----------------------------TEP------------------------------------------------------SAQQNMSL---------------------AILRIIRLVRVFRIFKLS-RHSKGL--------------------QILGQTLKA--------SMRELGLLIFFL----FIG--VILFSSAVYFA--EV-------------------------------------------------------------------------DEPES-----------------------------------------------------------------------HFSSIPDGFWWAVVTMTTVGYGDMCPTTPGGKIVG-TLCAIAGVLTIALPVPVIVSNFNYFYHR--------------------------------
126311565            --------------NRPSFDGILYYYQ------------SGGKI---RRPANVPIDVFADEISFYELGVEAMDQFREDEG-----------------------------FIKDPKTQ------------------------------------------------------------------------LPKNDFH---------RQFWLLFEYPESSSAARGVAVVSVLVVVISITIFCLETLPEFREERELKVVRDPS-----------------------LNLSKTVLSHN-----------------------------------MFTDPFFMVESTCIIWFTFELVLRFVVC-----------------PSKTGFFRNIM--------------------NIIDIISIIPYFVTLITELVQE-----------------------------TEP------------------------------------------------------SAQQNMS---------------------LAILRIIRLVRVFRIFKLS-RHSKGL--------------------QILGQTLKA--------SMRELGLLIFFL----FIG--VILFSSAVYFA--EV-------------------------------------------------------------------------DEPES-----------------------------------------------------------------------HFSSIPDGFWWAVVTMTTVGYGDMCPTTPGGKIVG-TLCAIAGVLTIALPVPVIVSNF----MKVSENK---------------------------
148669948            --------------NRPSFDGILYYYQ------------SGGKI---RRPANVPIDVFADEISFYELGSEAMDQFREDEG-----------------------------FIKDPETL------------------------------------------------------------------------LPTNDFH---------RQFWLLFD----------------------------------------------------------------------INTNKTGLSQT-----------------------------------MFTDPFFMGESTCIVWFTFELVLRFVVC-----------------PSKTDFFKNIM--------------------NIIDIISIIPYFATLITELVQE-----------------------------TEP------------------------------------------------------SAQQNMSL---------------------AILRIIRLVRVFRIFKLS-RHSKGL--------------------QILGQTLKA--------SMRELGLLIFFL----FIG--VILFSSAVYFA--EV-------------------------------------------------------------------------DEPES-----------------------------------------------------------------------HFSSIPDGFWWAVVTMTTVGYGDMCPTTPGGKIVG-TLCAIAGVLTIALPVPVIVSNFNYFYHRETENEEKPNI----------------------
1098962_404_694      --------------DFHRQFWLLYPVK------------GAPHA---ARTVAV-VSVLVVVISITIFCLVTLARVPGGQG------------------------------AEGSSE-------------------------------------------------------------------------TPASATN-------------------------------------------------------------------------------------------KTAFPQT-----------------------------------MFTDPFFMVESTCIVWFTLELVLRFVVC-----------------PSKTAFYRNIM--------------------NIIDIISIIPYFATLITELVQE-----------------------------TEP------------------------------------------------------SAQQNMSL---------------------AILRIIRLVRVFRIFKLS-RHSKGL--------------------QILGQTLKA--------SMRELGLLIFFL----FIG--VILFSSAVYFA--EV-------------------------------------------------------------------------DEPES-----------------------------------------------------------------------HFSSIPDGFWWAVVTMTTVGYGDMCPITPGGKIVG-TLCAIAGVLTIALPVPVIVSNFNYFYHR--------------------------------
4504817_284_583      --------------EFKKQIWLLFEYP-----------ESSSP----ARGIAI-VSVLVILISIVIFCLETLPEFRDDRDL-----------------------------VMALSA--------------------------------------------------------------------------GGHGGLL-----------------------------------------------------------------------------------------NDTSAPHLENSGH-----------------------------TIFNDPFFIVETVCIVWFSFEFVVRCFAC-----------------PSQALFFKNIM--------------------NIIDIVSILPYFITLGTDLAQQ-----------------------------QG---------------------------------------------------GGNGQQQQAMS---------------------FAILRIIRLVRVFRIFKLS-RHSKGL--------------------QILGHTLRA--------SMRELGLLIFFL----FIG--VILFSSAVYFA--EA-------------------------------------------------------------------------DEPTT-----------------------------------------------------------------------HFQSIPDAFWWAVVTMTTVGYGDMKPITVGGKIVG-SLCAIAGVLTIALPVPVIVSNFNYFYHR--------------------------------
116430_284_583       --------------EFKKQIWLLFEYP-----------ESSDP----ARGIAI-VSVLVILISIVIFCLETLPEFRDDRDL-----------------------------VMALSA--------------------------------------------------------------------------GGHGGLL-----------------------------------------------------------------------------------------NDTSAPHLENSGH-----------------------------TIFNDPFFIVETVCIVWFSFEFVVRCFAC-----------------PSQALFFKNIM--------------------NIIDIVSILPYFITLGTDLAQQ-----------------------------QG---------------------------------------------------GGNGQQQQAMS---------------------FAILRIIRLVRVFRIFKLS-RHSKGL--------------------QILGHTLRA--------SMRELGLLIFFL----FIG--VILFSSAVYFA--EA-------------------------------------------------------------------------DEPTT-----------------------------------------------------------------------HFQSIPDAFWWAVVTMTTVGYGDMKPITVGGKIVG-VLCAIAGVLTIALPVPVIVSNFNYFYHR--------------------------------
21311753_274_573     --------------EFKKQIWLLFEYP-----------ESSSP----ARGIAI-VSVLVILISIVIFCLETLPEFRDDRDL-----------------------------VMALSA--------------------------------------------------------------------------GGHGGLL-----------------------------------------------------------------------------------------NDTLAPHPENSGH-----------------------------TIFNDPFFIVETVCIVWFSFEFVVRCFAC-----------------PSQALFFKNIM--------------------NIIDIVSILPYFITLGTDLAQQ-----------------------------QG---------------------------------------------------GGNGQQQQAMS---------------------FAILRIIRLVRVFRIFKLS-RHSKGL--------------------QILGHTLRA--------SMRELGLLIFFL----FIG--VILFSSAVYFA--EA-------------------------------------------------------------------------DEPTT-----------------------------------------------------------------------HFQSIPDAFWWAVVTMTTVGYGDMKPITVGGKIVG-SLCAIAGVLTTALPVPVIVSNFNYFYHR--------------------------------
9931499_293_592      --------------EFKKQIWLLFEYP-----------ESSSP----ARGIAI-VSVLVILISIVIFCLETLPEFRDDRDL-----------------------------IMALST--------------------------------------------------------------------------GGHGGLL-----------------------------------------------------------------------------------------NDTSAPHPENSGH-----------------------------TIFNDPFFIVETVCIVWFSFEFVVRCFAC-----------------PSQALFFKNIM--------------------NIIDIVSILPYFITLGTDLAQQ-----------------------------QG---------------------------------------------------GGNGQQQQAMS---------------------FAILRIIRLVRVFRIFKLS-RHSKGL--------------------QILGHTLRA--------SMRELGLLIFFL----FIG--VILFSSAVYFA--EA-------------------------------------------------------------------------DEPTT-----------------------------------------------------------------------HFQSIPDAFWWAVVTMTTVGYGDMKPITVGGKIVG-SLCAIAGVLTIALPVPVIVSNFNYFYHR--------------------------------
31543026_285_584     --------------EFKKQIWLLFEYP-----------ESSSP----ARGIAI-VSVLVILISIVIFCLETLPEFRDDRDL-----------------------------IMALSA--------------------------------------------------------------------------GGHSRLL-----------------------------------------------------------------------------------------NDTSAPHLENSGH-----------------------------TIFNDPFFIVETVCIVWFSFEFVVRCFAC-----------------TSQALFFKNIM--------------------NIIDIVSILPYFITLGTDLAQQ-----------------------------QG---------------------------------------------------GGNGQQQQAMS---------------------FAILRIIRLVRVFRIFKLS-RHSKGL--------------------QILGHTLRA--------SMRELGLLIFFL----FIG--VILFSSAVYFA--EA-------------------------------------------------------------------------DEPTT-----------------------------------------------------------------------HFQSIPDAFWWAVVTMTTVGYGDMKPITVGGKIVG-SLCAIAGVLTIALPVPVIVSNFNYFYHR--------------------------------
20911111_285_584     --------------EFKKQIWLLFEYP-----------ESSSP----ARGIAI-VSVLVILISIVIFCLETLPEFRDDRDL-----------------------------IMALSA--------------------------------------------------------------------------GGHSRLL-----------------------------------------------------------------------------------------NDTSAPHLENSGH-----------------------------TIFNDPFFIVETVCIVWFSFEFVVRCFAC-----------------PSQALFFKNIM--------------------NIIDIVSILPYFITLGTDLAQQ-----------------------------QG---------------------------------------------------GGNGQQQQAMS---------------------FAILRIIRLVRVFRIFKLS-RHSKGL--------------------QILGHTLRA--------SMRELGLLIFFL----FIG--VILFSSAVYFA--EA-------------------------------------------------------------------------DEPTT-----------------------------------------------------------------------HFQSIPDAFWWAVVTMTTVGYGDMKPITVGGKIVG-SLCAIAGVLTIALPVPVIVSNFNYFYHR--------------------------------
6981116_285_584      --------------EFKKQIWLLFEYP-----------ESSSP----ARAIAI-VSVLVILISIVIFCLETLPEFRDDRDL-----------------------------IMALSA--------------------------------------------------------------------------GGHSRLL-----------------------------------------------------------------------------------------NDTSAPHLENSGH-----------------------------TIFNDPFFIVETVCIVWFSFEFVVRCFAC-----------------PSQALFFKNIM--------------------NIIDIVSILPYFITLGTDLAQQ-----------------------------QG---------------------------------------------------GGNGQQQQAMS---------------------FAILRIIRLVRVFRIFKLS-RHSKGL--------------------QILGHTLRA--------SMRELGLLIFFL----FIG--VILFSSAVYFA--EA-------------------------------------------------------------------------DEPTT-----------------------------------------------------------------------HFQSIPDAFWWAVVTMTTVGYGDMKPITVGGKIVG-SLCAIAGVLTIALPVPVIVSNFNYFYHR--------------------------------
3023496_285_584      --------------EFKKQIWLLFEYP-----------ESSSP----ARGIAI-VSVLVILISIVIFCLETLPEFRDDRDL-----------------------------IMALSA--------------------------------------------------------------------------GGHSGLL-----------------------------------------------------------------------------------------NDTSAPHLENSGH-----------------------------TIFNDPFFIVETVCIVWFSFEFVVRCFAC-----------------PSQALFFKNIM--------------------NIIDIVSILPYFITLGTDLAQQ-----------------------------QG---------------------------------------------------GGNGQQQQAMS---------------------FAILRIIRLVRVFRIFKLS-RHSKGL--------------------QILGHTLRA--------SMRELGLLIFFL----FIG--VILFSSAVYFA--EA-------------------------------------------------------------------------DEPTT-----------------------------------------------------------------------HFQSIPDAFWWAVVTMTTVGYGDMKPITVGGKIVG-SLCAIAGVLTIALPVPVIVSNFNYFYHR--------------------------------
4204389_300_592      --------------EFKRQVWLLFEYP-----------ESSSP----ARGIAI-VSVLVILISIVIFCLETLPEFRDDKEF-----------------------------IMSLSS--------------------------------------------------------------------------G--KGIS-----------------------------------------------------------------------------------------NES--LHLDDGEH-----------------------------TIFNDPFFIVETVCIIWFSFEFTVRCFAC-----------------PSKAHFLKNIM--------------------NIIDIVSILPYFITLGTDLVQE-----------------------------QG----------------------------------------------------SNGQQ--AMS---------------------FAILRIIRLVRVFRIFKLS-RHSKGL--------------------QILGHTLRA--------SMRELGLLIFFL----FIG--VILFSSAVYFA--EA-------------------------------------------------------------------------DEPAT-----------------------------------------------------------------------HFQSIPDAFWWAVVTMTTVGYGDMKPITVGGKIVG-SLCAIAGVLTIALPVPVIVSNFNYFYHR--------------------------------
16903202_300_592     --------------EFKRQVWLLFEYP-----------ESSSP----ARGIAI-VSVLVILISIVIFCLETLPEFRDDKEF-----------------------------IMPLSL--------------------------------------------------------------------------G--KGLS-----------------------------------------------------------------------------------------NES--LHLDAGEH-----------------------------TIFNDPFFIVETVCIIWFSFEFTVRCFAC-----------------PSKAHFFKNIM--------------------NIIDIVSILPYFITLGTDLAQE-----------------------------QG----------------------------------------------------SNGQQ--AMS---------------------FAILRIIRLVRVFRIFKLS-RHSKGL--------------------QILGHTLRA--------SMRELGLLIFFL----FIG--VILFSSAVYFA--EA-------------------------------------------------------------------------DEPAT-----------------------------------------------------------------------HFQSIPDAFWWAVVTMTTVGYGDMKPITVGGKIVG-SLCAIAGVLTIALPVPVIVSNFNYFYHR--------------------------------
22252948_305_627     --------------EFKKQVWLLFEYP-----------ESSGA----ARGIAI-VSVLVILISTVIFCLETLPEFRDDKDNLLSPLG------------------------MGDDDG-------------------------------------------------------------------------AGEDGEG-----------------------------------------------------------------------------------------GAYNATFLSTDSG----------------------------HTAFNDPFFIVETVCIVWFSFEFAVRLFAC-----------------PSKPEFFKNIM--------------------NIIDIVSILPYFITLGTELGQQ--------------------------HPPQQQQ---------------------------HLALATGQQLP-----------QGTGQQQQAMS---------------------FAILRIIRLVRVFRIFKLS-RHSKGL--------------------QILGHTLRA--------SMRELGLLIFFL----FIG--VILFSSAVYFA--EA-------------------------------------------------------------------------DEPTT-----------------------------------------------------------------------HFQSIPDAFWWAVVTMTTVGYGDMKPITVGGKIVG-SLCAIAGVLTIALPVPVIVSNFNYFYHR--------------------------------
13021995_285_607     --------------EFKKQVWLLFEYP-----------ESSGA----ARGIAI-VSVLVILISIVIFCLETLPEFRDDKDNLLSPLG------------------------MGDDDG-------------------------------------------------------------------------AGEDGEG-----------------------------------------------------------------------------------------GAYNATFLSTDSG----------------------------HTAFNDPFFIVETVCIVWFSFEFAVRLFAC-----------------PSKPEFFKNIM--------------------NIIDIVSILPYFITLGTEPGQQ--------------------------HPPQQQQ---------------------------HLALVTGQQLP-----------QGTGQQQQAMS---------------------FAILRIIRLVRVFRIFKLS-RHSKGL--------------------QILGHTLRA--------SMRELGLLILFL----FIG--VILFSSAVYFA--EA-------------------------------------------------------------------------DEPTT-----------------------------------------------------------------------HFQSIPDAFWWAVVTMTTVGYGDMKPTTVGGKIVG-SLCAIAGVLTIALPVPVIVSNFNYFYHR--------------------------------
47230729             --------------EFKRQIWLLFEYP-----------ESSSP----ARGIAV-VSVLVIVISIVIFCLETLPEFRDE--------------------------------KEYLQP-----------------------------------------------------------------------------RGN---------------------------------------------------------------------------------------------S-SRADHG------------------------------FTPFNDPFFIVETVCIIWFSFEIIVRFFAS-----------------PSKPAFFKNIM--------------------NSIDIVSILPYFITLGTDLAQH-----------------------------QGNG-------------------------------------------------------QQAMS---------------------FAILRIIRLVRVFRIFKLS-RHSKGL--------------------QILGHTLRA--------SMRELALLIFFL----VIG--VILFSSAVYFA--EA-------------------------------------------------------------------------DEPTSQ-----------------------------------------------------------------------FTSIPDAFWWAVVTMTTVGYGDMKPITVGGKIVG-SLCAIAGVLTIALPVPVIVSNFNYFYHRETDNEDQAPVVESVPP----------------
206549_140_432       --------------EFQRQVWLLFEYP-----------ESSGP----ARIIAI-VSVMVILISIVSFCLETLPIFRDENE------------------------------DMHGGG--------------------------------------------------------------------------VTFHTYS-----------------------------------------------------------------------------------------NSTIGYQQS---------------------------------TSFTDPFFIVETLCIIWFSFEFLVRFFAC-----------------PSKAGFFTNIM--------------------NIIDIVAIIPYFITLGTELAEK-----------------------------PED----------------------------------------------------AQQGQQAMS---------------------LAILRVIRLVRVFRIFKLS-RHSKGL--------------------QILGQTLKA--------SMRELGLLIFFL----FIG--VILFSSAVYFA--EA-------------------------------------------------------------------------DERDS-----------------------------------------------------------------------QFPSIPDAFWWAVVSMTTVGYGDMVPTTIGGKIVG-SLCAIAGVLTIALPVPVIVFNFNYFYHR--------------------------------
6680514_140_432      --------------EFQRQVWLLFEYP-----------ESSGP----ARIIAI-VSVMVILISIVSFCLETLPIFRDENE------------------------------DMHGGG--------------------------------------------------------------------------VTFHTYS-----------------------------------------------------------------------------------------NSTIGYQQS---------------------------------TSFTDPFFIVETLCIIWFSFEFLVRFFAC-----------------PSKAGFFTNIM--------------------NIIDIVAIIPYFITLGTELAEK-----------------------------PED----------------------------------------------------AQQGQQAMS---------------------LAILRVIRLVRVFRIFKLS-RHSKGL--------------------QILGQTLKA--------SMRELGLLIFFL----FIG--VILFSSAVYFA--EA-------------------------------------------------------------------------DERDS-----------------------------------------------------------------------QFPSIPDAFWWAVVSMTTVGYGDMVPTTIGGKIVG-SLCAIAGVLTIALPVPVIVSNFNYFYHR--------------------------------
109013443            --------------EFQRQVWLLFEYP-----------ESSGP----ARIIAI-VSVMVILISIVSFCLETLPIFRDENE------------------------------DMHGGG--------------------------------------------------------------------------VTFHTYS-----------------------------------------------------------------------------------------NSTIGYQQS---------------------------------TSFTDPFFIVETLCIIWFSFEFLVRFFAC-----------------PSKAGFFTNIM--------------------NIIDIVAIIPYFITLGTELAEK-----------------------------PED----------------------------------------------------AQQGQQAMS---------------------LAILRVIRLVRVFRIFKLS-RHSKGL--------------------QILGQTLKA--------SMRELGLLIFFL----FIG--VILFSSAVYFA--EA-------------------------------------------------------------------------DERES-----------------------------------------------------------------------QFPSIPDAFWWAVVSMTTVGYGDMVPTTIGGKIVG-SLCAIAGVLTIALPVPVIVSNFNYFYHR--------------------------------
4826782_140_432      --------------EFQRQVWLLFEYP-----------ESSGP----ARIIAI-VSVMVILISIVSFCLETLPIFRDENE------------------------------DMHGSG--------------------------------------------------------------------------VTFHTYS-----------------------------------------------------------------------------------------NSTIGYQQS---------------------------------TSFTDPFFIVETLCIIWFSFEFLVRFFAC-----------------PSKAGFFTNIM--------------------NIIDIVAIIPYFITLGTELAEK-----------------------------PED----------------------------------------------------AQQGQQAMS---------------------LAILRVIRLVRVFRIFKLS-RHSKGL--------------------QILGQTLKA--------SMRELGLLIFFL----FIG--VILFSSAVYFA--EA-------------------------------------------------------------------------DERES-----------------------------------------------------------------------QFPSIPDAFWWAVVSMTTVGYGDMVPTTIGGKIVG-SLCAIAGVLTIALPVPVIVSNFNYFYHR--------------------------------
395207               --------------EFQRQVWLLFEYP-----------ESSGP----ARIIAI-VSVMVILISIVSFCLETLPIFRDENE------------------------------DMHGSG--------------------------------------------------------------------------VTFHTYS-----------------------------------------------------------------------------------------NSTIGYQQS---------------------------------TSFTDPFFIVETLCIIWFSFEFLVRFFAC-----------------PSKAGFFTNIM--------------------NIIDIVAIIPYFTTLGTELAEK-----------------------------PED----------------------------------------------------AQQGQQAMS---------------------LAILRVIRLVRVFRIFKLS-RHSKGL--------------------QILGQSLKA--------SMRELGLLIFFL----FIG--VILFSSAVYFA--EA-------------------------------------------------------------------------DERDS-----------------------------------------------------------------------QFPSIPDAFWWAVVSMTTVGYGDMVPTTIGGKIVG-SLCAIAGVLTIALPVPVIVSNFNYFYHR--------------------------------
9652317_140_432      --------------EFQRQVWLLFEYP-----------ESSGP----ARIIAI-VSVMVILISIVSFCLETLPIFRDENE------------------------------DMHGSG--------------------------------------------------------------------------MTFHTYS-----------------------------------------------------------------------------------------NSTAGYQQS---------------------------------TSFTDPFFIVETLCIIWFSFEFLVRFFAC-----------------PSKAGFFTNIM--------------------NIIDIVAIIPYFITLGTELAEK-----------------------------PED----------------------------------------------------AQQGQQAMS---------------------LAILRVIRLVRVFRIFKLS-RHSKGL--------------------QILGQTLKA--------SMRELGLLIFFL----FIG--VILFSSAVYFA--EA-------------------------------------------------------------------------DERDS-----------------------------------------------------------------------QFPSIPDAFWWAVVSMTTVGYGDMVPTTIGGKIVG-SLCAIAGVLTIALPVPVIVSNFNYFYHR--------------------------------
Kv_1221_144_417      --------------EFQRQVWLLFEYP-----------ESSGP----ARIIAI-VSVMVILISIVSFCLETLPIFRDENE------------------------------DMHGGG--------------------------------------------------------------------------VTFHTYS-----------------------------------------------------------------------------------------NSTIGYQQ---------------------------------STSFTDPFFIVETLCIIWFSFEFLVRFFAC-----------------PSKAGFFTNIM--------------------NIIDIVAIIPYYVTIFLTESNK-----------------------------S------------------------------------------------------VLQFQNV-----------------------RRVVQIFRIMRILRIFKLS-RHSKGL--------------------QILGQTLKA--------SMRELGLLIFFL----FIG--VILFSSAVYFA--EA-------------------------------------------------------------------------DERD-----------------------------------------------------------------------SQFPSIPDAFWWAVVSMTTVGYGDMVPTTIGGKIVG-SLCAIAGVLTIALPVPVIVSNFNYFYHRETEGEEQAQYLQVT------------------
544035_140_432       --------------EFQKQVWLLFEYP-----------ESSGP----ARIIAI-ISVTVILISIVSFCLETLPVFRDENE------------------------------DMHGSG--------------------------------------------------------------------------GNYYSYP-----------------------------------------------------------------------------------------NSTVRFQKS---------------------------------NTFTDPFFIVETLCIIWFSFEFLVRFLAC-----------------PSKAVFFTNLM--------------------NIIDIVAIIPYFITLGTELAEK-----------------------------TED----------------------------------------------------GQQGQQAMS---------------------LAILRVIRLVRVFRIFKLS-RHSKGL--------------------QILGQTLNA--------SMRELGLLIFFL----FIG--VILFSSAVFFA--EA-------------------------------------------------------------------------DERDS-----------------------------------------------------------------------QFPSIPDAFWWAVVSMTTVGYGDMVPTTIGGKIVG-SLCAIAGVLTIALPVPVIVSNFNYFYHR--------------------------------
104160_140_432       --------------EFQKQVWLLFEYP-----------ESSGP----ARIIAI-ISVTVILISIVSFCLETLPVFRDENE------------------------------NMHGSG--------------------------------------------------------------------------GNYYSYP-----------------------------------------------------------------------------------------NSTVRFQKS---------------------------------NTFTDPFFIVETLCMIWFSFEFLVLVLAC-----------------PSKAVFFTNLM--------------------NIIDIVAIIPYFITLGTELAEK-----------------------------TED----------------------------------------------------GQQGQQAMS---------------------LAILRVIRLVRVFRIFKLS-RHSKGL--------------------QILGQTLNA--------SMRELGLLIFFL----FIG--VILFSSAVFFA--EA-------------------------------------------------------------------------DERDS-----------------------------------------------------------------------QFPSIPDAFWWAVVSMTTVGYGDMVPTTIGGKIVG-SLCAIAGVLTIALPVPVIVSNFNYFYHR--------------------------------
13021992_140_432     --------------EFQKQVWLLFEYP-----------ESSGP----ARIIAI-ISVTVILISIVSFCLETLPVFRDENE------------------------------DMHGSG--------------------------------------------------------------------------GNYYSYP-----------------------------------------------------------------------------------------NSTVRFQKS---------------------------------NTFTDPFFIVETLCIIWFSFEFLVRFLAC-----------------PSKAVFFTNLM--------------------NIIDIVAIIPYFITLGTELAEK-----------------------------TED----------------------------------------------------GQQGQQAMS---------------------LAILRVIRLVRVFRIFKLS-RHSKGL--------------------QILGQTLKA--------SMRELGLLIFFL----FIG--VILFSSAVFFA--EA-------------------------------------------------------------------------DERDS-----------------------------------------------------------------------QFPSIPDAFWWAVVSMTTVGYGDMVPTTIGGKIVG-SLCAIAGVLTIALPVPVIVSNFNYFYHR--------------------------------
7800632_139_427      --------------EFQRQVWLLFEYP-----------ESSGP----ARIIAI-ISVMVILISIVSFCLETLPIFRNDDD------------------------------EPHS---------------------------------------------------------------------------VFDTNT-------------------------------------------------------------------------------------------NTTI-YFTS---------------------------------TYFTDPFFILETLCIIWFSFEFLVRLFAC-----------------PSKSGFFGNVM--------------------NIIDVVAIIPYFITLATELAEK-----------------------------PED----------------------------------------------------GQAGQQAMS---------------------LAILRVIRLVRVFRIFKLS-RHSKGL--------------------QILGQTLKA--------SMRELGLLIFFL----FIG--VILFSSAVYFA--EA-------------------------------------------------------------------------DEPES-----------------------------------------------------------------------QFESIPDAFWWAVVSMTTVGYGDMVPTTIGGKIVG-SLCAIAGVLTIALPVPVIVSNFNYFYHR--------------------------------
68401456             --------------EFQKQVWLLFEYP-----------ESSGP----ARIIAI-ISVMVILISIVSFCLETLPIFRSD------------------------------EEEMHK----------------------------------------------------------------------------VPVNV-------------------------------------------------------------------------------------------NNSTTSYTS---------------------------------TYFTDPFFILETLCIIWFSFEFLVRFFAC-----------------PSKASFFVNIM--------------------NMIDVVAIIPYFITLGTELAEK-----------------------------AEDGQ----------------------------------------------------QGQQAMS---------------------LAILRVIRLVRVFRIFKLS-RHSKGL--------------------QILGQTLKA--------SMRELGLLIFFL----FIG--VILFSSAVYFA--EA-------------------------------------------------------------------------DEPESQ-----------------------------------------------------------------------FYSIPDAFWWAVVSMTTVGYGDMVPTTIGGKIVG-SLCAIAGVLTIALPVPVIVSNFNYFYHRETEGEEQAQYLQVN------------------
4557685_144_430      --------------EYQRQVWLLFEYP-----------ESSGP----ARVIAI-VSVMVILISIVIFCLETLPELKDDKD-------------------------------FTGT-----------------------------------------------------------------------------VHRID-----------------------------------------------------------------------------------------NTTVIYNS----------------------------------NIFTDPFFIVETLCIIWFSFELVVRFFAC-----------------PSKTDFFKNIM--------------------NFIDIVAIIPYFITLGTEIAEQ-----------------------------EGN-----------------------------------------------------QKGEQATS---------------------LAILRVIRLVRVFRIFKLS-RHSKGL--------------------QILGQTLKA--------SMRELGLLIFFL----FIG--VILFSSAVYFA--EA-------------------------------------------------------------------------EEAES-----------------------------------------------------------------------HFSSIPDAFWWAVVSMTTVGYGDMYPVTIGGKIVG-SLCAIAGVLTIALPVPVIVSNFNYFYHR--------------------------------
119395752_144_430    --------------EYQRQVWLLFEYP-----------ESSGP----ARVIAI-VSVMVILISIVIFCLETLPELKDDKD-------------------------------FTGT-----------------------------------------------------------------------------IHRID-----------------------------------------------------------------------------------------NTTVIYTS----------------------------------NIFTDPFFIVETLCIIWFSFELVVRFFAC-----------------PSKTDFFKNIM--------------------NFIDIVAIIPYFITLGTEIAEQ-----------------------------EGN-----------------------------------------------------QKGEQATS---------------------LAILRVIRLVRVFRIFKLS-RHSKGL--------------------QILGQTLKA--------SMRELGLLIFFL----FIG--VILFSSAVYFA--EA-------------------------------------------------------------------------EEAES-----------------------------------------------------------------------HFSSIPDAFWWAVVSMTTVGYGDMYPVTIGGKIVG-SLCAIAGVLTIALPVPVIVSNFNYFYHR--------------------------------
148223679            --------------EFQRQVWLLFEFP-----------ESSGP----ARIIAI-ISVMVILISIVIFCLETLPELKDERI-------------------------------FS--------------------------------------------------------------------------------RRVN-----------------------------------------------------------------------------------------NSTVFYKS----------------------------------NIFTDPFFVVETLCIIWFSFELVVRFFAC-----------------PSKPEFFKNIM--------------------NFIDIVAIIPYFITLGTEMAEQ-----------------------------EGP-----------------------------------------------------QKGEQATS---------------------LAILRVIRLVRVFRIFKLS-RHSKGL--------------------QILGQTLKA--------SMRELGLLIFFL----FIG--VILFSSAVYFA--EA-------------------------------------------------------------------------EEDES-----------------------------------------------------------------------HFTSIPDAFWWAVVSMTTVGYGDMYPVTIGGKIVG-SLCAIAGVLTIALPVPVIVSNFNYFYHR--------------------------------
47224426             --------------EFQRQIWLLFEHP-----------ESSGT----ARGIAI-VSVMVILISIVIFCLETLPQLKDD---------------------------------PAGR-----------------------------------------------------------------------------IMIVG-----------------------------------------------------------------------------------------NTTIRYKA----------------------------------NILTDPFFITETLCIIWFSFELIVRFLAC-----------------PSKPAFFKNMM--------------------NMIDIVAIIPYFITLGTELAED-----------------------------PKN----------------------------------------------------EGVGEQATS---------------------LAILRVIRLVRVFRIFKLS-RHSKGL--------------------QILGQTLKA--------SMRELGLLIFFL----FIG--VILFSSAVYFA--EV-------------------------------------------------------------------------EEQGSY-----------------------------------------------------------------------FGSIPDAFWWAVVSMTTVGYGDMVPVTIGGKIVG-SLCAIAGVLTIALPVPVIVSNFNYFYHRETEGEEQAQL----------------------
14866941             --------------DFQRQVWLLFEYP-----------ESSGP----ARGIAI-VSVLVILISIVIFCLETLPEFRDE--------------------------------KDYPAS--------------------------------------------------------------------------PSQDVF--------------------------------------------------------------------------------------EAANNSTSGASSG--------------------------------ASSFSDPFFVVETLCIIWFSFELLVRFFAC-----------------PSKATFSRNIM--------------------NLIDIVAIIPYFITLGTELAER-----------------------------QGN-------------------------------------------------------GQQAMS---------------------LAILRVIRLVRVFRIFKLS-RHSKGL--------------------QILGQTLKA--------SMRELGLLIFFL----FIG--VILFSSAVYFA--EA-------------------------------------------------------------------------DDPSS-----------------------------------------------------------------------GFNSIPDAFWWAVVTMTTVGYGDMHPVTIGGKIVG-SLCAIAGVLTIALPVPVIVSNFNYFYHR--------------------------------
206635_161_452       --------------DFQRQVWLLFEYP-----------ESSRP----ARGIAI-VSVLVILISIVIFCLETLPEFRDE--------------------------------KDYPAS--------------------------------------------------------------------------PSQDVF--------------------------------------------------------------------------------------EAANNSTSGASSG--------------------------------ASSFSDPFFVVETLCIIWFSFELLVRFFAC-----------------PSKATFSRNIM--------------------NLIDIVAIIPYFITLGTELAER-----------------------------QGN-------------------------------------------------------GQQAMS---------------------LAILRVIRLVRVFRIFKLS-RHSKGL--------------------QILGQTLKA--------SMRELGLLIFFL----FIG--VILFSSAVYFA--EA-------------------------------------------------------------------------DDPSS-----------------------------------------------------------------------GFNSIPDAFWWAVVTMTTVGYGDMHPVTIGGKIVG-SLCAIAGVLTIALPVPVILSNFNYFYHR--------------------------------
20875187_75_366      --------------DFQRQVWLLFEYP-----------ESSGP----ARGIAI-VSVLVILISIVIFCLETLPEFRDE--------------------------------KDYPAS--------------------------------------------------------------------------PSQDVF--------------------------------------------------------------------------------------EAANNSTSGAPSG--------------------------------ASSFSDPFFVVETLCIIWFSFELLVRFFAC-----------------PSKATFSRNIM--------------------NLIDIVAIIPYFITLGTELAER-----------------------------QGN-------------------------------------------------------GQQAMS---------------------LAILRVIRLVRVFRIFKLS-RHSKGL--------------------QILGQTLKA--------SMRELGLLIFFL----FIG--VILFSSAVYFA--EA-------------------------------------------------------------------------DDPSS-----------------------------------------------------------------------GFNSIPDAFWWAVVTMTTVGYGDMHPVTIGGKIVG-SLCAIAGVLTIALPVPVIVSNFNYFYHR--------------------------------
6680516_164_455      --------------DFQRQVWLLFEYP-----------ESSGP----ARGIAI-VSVLVILISIVIFCLETLPEFRDE--------------------------------KDYPAS--------------------------------------------------------------------------PSQDVF--------------------------------------------------------------------------------------EAANNSTSGAPSG--------------------------------ASSFSDPFFVVETLCIIWFSFELLVRFFAC-----------------PSKATFSRNIM--------------------NLIDIVAIIPYFITLGTELAER-----------------------------QGN-------------------------------------------------------GQQAMS---------------------LAILRVIRLVRVFRIFKLS-RHSKGL--------------------QILGQTLKA--------SMRELGLLIFFL----FIG--VILFSSAAYFA--EA-------------------------------------------------------------------------DDPSS-----------------------------------------------------------------------GFNSIPDAFWWAVVTMTTVGYGDMHPVTIGGKIVG-SLCAIAGVLTIALPVPVIVSNFNYFYHR--------------------------------
385223_159_450       --------------DFQRQVWLLFEYP-----------ESSGP----ARGIAI-VSVLVILISIVIFCLETLPEFRDE--------------------------------KDYPAS--------------------------------------------------------------------------TSQDSF--------------------------------------------------------------------------------------EAAGNSTSGSRAG--------------------------------ASSFSDPFFVVETLCIIWFSFKLLVRFFAC-----------------PSKATFSRNIM--------------------NLIDIVAIIPYFITLGTELAER-----------------------------QGN-------------------------------------------------------GQQAMS---------------------LAILRVIRLVRVFRIFKLS-RHSKGL--------------------QILGQTLKA--------SMRELGLLIFFL----FIG--VILFSSAVYFA--EA-------------------------------------------------------------------------DDPTS-----------------------------------------------------------------------GFSSIPDAFWWAVVTMTTVGYGDMHPVTIGGKIVG-SLCAIAGVLTIALPVPVIVSNFNYFYHR--------------------------------
189673_159_450       --------------DFQRQVWLLFEYP-----------ESSGP----ARGIAI-VSVLVILISIVIFCLETLPEFRDE--------------------------------KDYPAS--------------------------------------------------------------------------TSQDSF--------------------------------------------------------------------------------------EAAGNSTSGSRAG--------------------------------ASSFSDPFFVVETLCIIWFSFELLVRFFAC-----------------PSKATFSRNIM--------------------NLIDIVAIIPYFITLGTELAER-----------------------------QGN-------------------------------------------------------GQQAMS---------------------LAILRVIRLVRVFRIFKLS-RHSKGL--------------------QILGQSLKA--------SMRELGLLIFFL----FIG--VILFSSAVYFA--EA-------------------------------------------------------------------------DDPTS-----------------------------------------------------------------------GFSSIPDAFWWAVVTMTTVGYGDMHPVTIGGKIVG-SLCAIAGVLSIALPVPVIVSNFNYFYHR--------------------------------
1168948_159_450      --------------DFQRQVWLLFEYP-----------ESSGP----ARGIAI-VSVLVILISIVIFCLETLPEFRDE--------------------------------KDYPAS--------------------------------------------------------------------------TSQDSF--------------------------------------------------------------------------------------EAAGNSTSGSRAG--------------------------------ASSFSDPFFVVETLCIIWFSFELLVRFFAC-----------------PSKATFSRNIM--------------------NLIDIVAIIPYFITLGTELAER-----------------------------QGN-------------------------------------------------------GQQAMS---------------------LAILRVIRLVRVFRIFKLS-RHSKGL--------------------QILGQTLKA--------SMRELGLLIFFL----FIG--VILFSSAVYFA--EA-------------------------------------------------------------------------DDPTS-----------------------------------------------------------------------GFSSIPDAFWWAVVTMTTVGYGDMHPVTIGGKIVG-SLCAIAGVLTIALPVPVIVSNFNYFYHR--------------------------------
3264841_152_443      --------------DFQRQVWLLFEYP-----------ESSGP----ARGIAI-VSVLVILISIVIFCLETLPEFRDE--------------------------------KDYPAA--------------------------------------------------------------------------PSQDVV--------------------------------------------------------------------------------------EAAGNGTSGAPAG--------------------------------ASSFSDPFFVVETLCIIWFSFELLVRFFAC-----------------PSKATFSRNIM--------------------NLIDIVAIIPYFITLGTELAER-----------------------------QGN-------------------------------------------------------GQQAMS---------------------LAILRVIRLVRVFRIFKLS-RHSKGL--------------------QILGQTLKA--------SMRDVGLLIFFL----FIG--VILFSSAVYFA--EA-------------------------------------------------------------------------DDPTS-----------------------------------------------------------------------GFSSIPDAFWWAVVTMTTVGYGDMHPVTIGGKIVG-SLCAIAGVLTIALPVPVIVSNFNYFYHR--------------------------------
15077851_141_425     --------------EFQRQVWLLFEYP-----------ESSGP----ARGIAI-VSVLVILISIVIFCLETLPEFRDD--------------------------------KEFSGA---------------------------------------------------------------------------S------------------------------------------------------------------------------------------VTPVNGTGRFIS----------------------------------SPFTDPFFVVETLCIIWFSFELLVRFFAC-----------------PSKAVFSKNIM--------------------NIIDIVAIIPYFITLGTELAEH-----------------------------QGN-------------------------------------------------------GQQAMS---------------------LAILRVIRLVRVFRIFKLS-RHSKGL--------------------QILGQTLKA--------SMRELGLLIFFL----FIG--VILFSSAVYFA--EA-------------------------------------------------------------------------DDPSS-----------------------------------------------------------------------GFNSIPDAFWWAVVTMTTVGYGDMHPVTIGGKIVG-SLCAIAGVLTIALPVPVIVSNFNYFYHR--------------------------------
94450651             --------------EFQRQVWLLFEYP-----------ESSGP----ARGIAI-VSVLVILISIVIFCLETLPEFRDD--------------------------------KDPVTV---------------------------------------------------------------------------API-------------------------------------------------------------------------------------------ANVTGSYGS----------------------------------SSFTDSFFVIETLCIIWFSFELLVRFFAC-----------------PSKATFSKNIM--------------------NIIDIVAIIPYFITLGTELAER-----------------------------QGNG-------------------------------------------------------QQAMS---------------------LAILRVIRLVRVFRIFKLS-RHSKGL--------------------QILGQTLKA--------SMRELGLLIFFL----FIG--VILFSSAVYFA--EA-------------------------------------------------------------------------DDPASS-----------------------------------------------------------------------FSSIPDAFWWAVVTMTTVGYGDMHPVTIGGKIVG-SLCAIAGVLTIALPVPVIVSNFNYFYHRETDGEEHAQYLHVGS-----------------
55637687             --------------EFQRQVWLIFEYP-----------ESSGS----ARAIAI-VSVLVILISIITFCLETLPEFRDERE-----------------------------LLRHPPA---------------------------------------------------------PHQPP-----------APAPGANG-------------------------------------------------------------------------------------SGVMAPPSGPTVAPLLP-----------------------------RTLADPFFIVETTCVIWFTFELLVRFFAC-----------------PSKAGFSRNIM--------------------NIIDVVAIFPYFITLGTELAEQ----------------------------QPGGG-------------------------------------------------GGGQNGQQAMS---------------------LAILRVIRLVRVFRIFKLS-RHSKGL--------------------QILGKTLQA--------SMRELGLLIFFL----FIG--VILFSSAVYFA--EA-------------------------------------------------------------------------DNQGT-----------------------------------------------------------------------HFSSIPDAFWWAVVTMTTVGYGDMRPITVGGKIVG-SLCAIAGVLTIALPVPVIVSNFNYFYHR--------------------------------
25952087_227_539     --------------EFQRQVWLIFEYP-----------ESSGS----ARAIAI-VSVLVILISIITFCLETLPEFRDERE-----------------------------LVRHPPA---------------------------------------------------------PHQPP-----------APAPGANG-------------------------------------------------------------------------------------SGVMAPPSGPTVAPLLP-----------------------------RTLADPFFIVETTCVIWFTFELLVRFFAC-----------------PSKAGFSRNIM--------------------NIIDVVAIFPYFITLGTELAEQ----------------------------QPGGG-------------------------------------------------GGGQNGQQAMS---------------------LAILRVIRLVRVFRIFKLS-RHSKGL--------------------QILGKTLQA--------SMRELGLLIFFL----FIG--VILFSSAVYFA--EA-------------------------------------------------------------------------DNQGT-----------------------------------------------------------------------HFSSIPDAFWWAVVTMTTVGYGDMRPITVGGKIVG-SLCAIAGVLTIALPVPVIVSNFNYFYHR--------------------------------
308765_225_537       --------------EFQRQVWLIFEYP-----------ESSGS----ARAIAI-VSVLVILISIITFCLETLPEFRDERE-----------------------------LLRHPPA---------------------------------------------------------PHQPP-----------APAPGANG-------------------------------------------------------------------------------------SGVMAPASGPTVAPLLP-----------------------------RTLADPFFIVETTCVIWFTFELLVRFFAC-----------------PSKAGFSRNIM--------------------NIIDVVAIFPYFITLGTELAEQ----------------------------QPGGG-------------------------------------------------GGGQNGQQAMS---------------------LAILRVIRLVRVFRIFKLS-RHSKGL--------------------QILGKTLQA--------SMRELGLLIFFL----FIG--VILFSSAVYFA--EA-------------------------------------------------------------------------DNQGT-----------------------------------------------------------------------HFSSIPDAFWWAVVTMTTVGYGDMRPITVGGKIVG-SLCAIAGVLTIALPVPVIVSNFNYFYHR--------------------------------
1705864_211_524      --------------EFQRQVWLIFEYP-----------ESSGS----ARAIAI-VSVLVILISIITFCLETLPEFKDERE-----------------------------LLRHPPV---------------------------------------------------------PHQPPA----------APALGANG-------------------------------------------------------------------------------------SGAVAPASGSTVAPLLP-----------------------------RTLADPFFIVETTCVIWFTFELLVRFFAC-----------------PSKAEFSRNIM--------------------NIIDIVAIFPYFITLGTELAEQ----------------------------QPGGG-------------------------------------------------GGGQNGQQAMS---------------------LAILRVIRLVRVFRIFKLS-RHSKGL--------------------QILGKTLQA--------SMRELGLLIFFL----FIG--VILFSSAVYFA--EA-------------------------------------------------------------------------DNQGT-----------------------------------------------------------------------HFSSIPDAFWWAVVTMTTVGYGDMRPITVGGKIVG-SLCAIAGVLTIALPVPVIVSNFNYFYHR--------------------------------
1363211_218_514      --------------EFQRQVWLIFEYP-----------ESSGS----ARAIAI-VSVSVILISIITFCLETLPEFRVDRE-----------------------------LLLHPPV---------------------------------------------------------PPQPP-----------APAPGTNA-------------------------------------------------------------------------------------SGSGVLSSGTTVAPLLP-----------------------------RTLADPFFIVETTCVIWFTFELLVRFFAC-----------------PSKAEFSRNIM--------------------NIIDIVAIFPYFITLGTELAEQ----------------------------QPG---------------------------------------------------GGGQNGQQAMS---------------------LAILRVIRLVRVFRIFKLS-RHSKGL--------------------QILGKTLQA--------SMRELGLLIFFL----FIG--VILFSSAVYFA--EA-------------------------------------------------------------------------DNQGS-----------------------------------------------------------------------QLSSIPDAFWWAVVTMTTVGYGDMRPITVGGKIVG-SLCAIAGVLTIALQQGLLLQDWG-------------------------------------
22122429_218_528     --------------EFQRQVWLIFEYP-----------ESSGS----ARAIAI-VSVSVILISIITFCLETLPEFRVDRE-----------------------------LLLHPPV---------------------------------------------------------PPQPP-----------APAPGTNA-------------------------------------------------------------------------------------SGSGVLSSGTTVAPLLP-----------------------------RTLADPFFIVETTCVIWFTFELLVRFFAC-----------------PSKAEFSRNIM--------------------NIIDIVAIFPYFITLGTELAEQ----------------------------QPG---------------------------------------------------GGGQNGQQAMS---------------------LAILRVIRLVRVFRIFKLS-RHSKGL--------------------QILGKTLQA--------SMRELGLLIFFL----FIG--VILFSSAVYFA--EA-------------------------------------------------------------------------DNQGS-----------------------------------------------------------------------QLSSIPDAFWWAVVTMTTVGYGDMRPITVGGKIVG-SLCAIAGVLTIALPVPVIVSNFNYFYHR--------------------------------
20832529_218_528     --------------EFQRQVWLIFEYP-----------ESSGS----ARAIAI-VSVLVILISIITFCLETLPEFRDERE-----------------------------LLRHPPV---------------------------------------------------------PPQPP-----------APAPGANG-------------------------------------------------------------------------------------SGSGVLSSGPTVAPLLP-----------------------------RTLADPFFIVETTCVIWFTFELLVRFFAC-----------------PSKAEFSRNIM--------------------NIIDIVAIFPYFITLGTELAEQ----------------------------QPG---------------------------------------------------GGGQNGQQAMS---------------------LAILRVIRLVRVFRIFKLS-RHSKGL--------------------QILGKTLQA--------SMRELGLLIFFL----FIG--VILFSSAVYFA--EA-------------------------------------------------------------------------DNQGS-----------------------------------------------------------------------HFSSIPDAFWWAVVTMTTVGYGDMRPITVGGKIVG-SLCAIAGVLTIALPVPVIVSNFNYFYHR--------------------------------
6981118_218_528      --------------EFQRQVWLIFEYP-----------ESSGS----ARAIAI-VSVLVILISIITFCLETLPEFRDERE-----------------------------LLRHPPV---------------------------------------------------------PPQPP-----------APAPGING-------------------------------------------------------------------------------------SVSGALSSGPTVAPLLP-----------------------------RTLADPFFIVETTCVIWFTFELLVRFFAC-----------------PSKAEFSRNIM--------------------NIIDVVAIFPYFITLGTELAEQ----------------------------QPG---------------------------------------------------GGGQNGQQAMS---------------------LAILRVIRLVRVFRIFKLS-RHSKGL--------------------QILGKTLQA--------SMRELGLLIFFL----FIG--VILFSSAVYFA--EA-------------------------------------------------------------------------DNHGS-----------------------------------------------------------------------HFSSIPDAFWWAVVTMTTVGYGDMRPITVGGKIVG-SLCAIAGVLTIALPVPVIVSNFNYFYHR--------------------------------
765314               --------------EFQRQVWLIFEYP-----------ESSGS----ARAIAI-VSVLVILISIITFCLETLPEFRDERE-----------------------------LLRHPPV---------------------------------------------------------PHQPP-----------GPHRGPNG-------------------------------------------------------------------------------------SG-AAAPSGPTVAPLLP-----------------------------RTLADPFFIVETTCVIWFTFELLVRFFAC-----------------PSKAEFSRNIM--------------------NIIDVVAIFPYFITLGTELVEQQ---------------------------QPGG--------------------------------------------------GGGQNGQQAMS---------------------LAILRVIRLVRVFRIFKLS-RHSKGL--------------------KILGKTLQA--------SMRELGLLIFFL----FIG--VILFSSAVYFA--EA-------------------------------------------------------------------------DNQEI-----------------------------------------------------------------------YFTSIPDAFWWAVVTMTTVGYGDMRPVTVGGKIVG-SLCAIAGVLTIALPVPVIVSNFNYFYHR--------------------------------
62751972             --------------EFQRQVWLIFEYP-----------ESSGS----ARAIAI-VSVLVILISIITFCLETLPEFRDERE-----------------------------LLRHPPV---------------------------------------------------------PHQPP-----------GPHRGPNG-------------------------------------------------------------------------------------SGAAAPPSGPTVAPLLP-----------------------------RTLADPFFIVETTCVIWFTFELLVRFFAC-----------------PSKAEFSRNIM--------------------NIIDVVAIFPYFITLGTELVEQQ---------------------------QPGGGG--------------------------------------------------GQNGQQAMS---------------------LAILRVIRLVRVFRIFKLS-RHSKGL--------------------QILGKTLQA--------SMRELGLLIFFL----FIG--VILFSSAVYFA--EA-------------------------------------------------------------------------DNQETH-----------------------------------------------------------------------FSSIPDAFWWAVVTMTTVGYGDMRPVTVGGKIVG-SLCAIAGVLTIALPVPVIVSNFNYFYHRETDHEEPAAVKEEQGS----------------
6685288_216_527      --------------EFQRQVWLIFEYP-----------ESSGS----ARGIAI-VSVLVILISIITFCLETLPEFRDERE-----------------------------LLRHPPV---------------------------------------------------------PHQPL-----------GPSRGANG-------------------------------------------------------------------------------------SGPLAPPSGPTVAPLLP-----------------------------RTLADPFFIVETTCVIWFTFELLVRFFAC-----------------PSKAEFSRNIM--------------------NIIDVVAIFPYFITLGTELAEQ-----------------------------PGGG-------------------------------------------------GGGQNGQQAMS---------------------LAILRVIRLVRVFRIFKLS-RHSKGL--------------------QILGKTLQA--------SMRELGLLIFFL----FIG--VILFSSAVYFA--EA-------------------------------------------------------------------------DNQET-----------------------------------------------------------------------HFSSIPDAFWWAVVTMTTVGYGDMRPVTVGGKIVG-SLCAIAGVLTIALPVPVIVSNFNYFYHR--------------------------------
603152_213_520       --------------EFQRQVWLIFEYP-----------ESSGS----ARAIAI-VSVLVILISIITFCLETLPEFRDERE-----------------------------LVRHPPG---------------------------------------------------------PPHPP-----------GPARGPTQ-------------------------------------------------------------------------------------RG-----RGPTVAPLLP-----------------------------RTLADPFFIVETTCVIWFTFELLVRFFAC-----------------PSKAEFSRNIM--------------------NIIDVVAIFPYFITLGTELAEQ----------------------------QPGGG-------------------------------------------------GGGQNGQQAMS---------------------LAILRVIRLVRVFRIFKLS-RHSKGL--------------------QILGKTLQA--------SMRELGLLIFFL----FIG--VILFSSAVYFA--EA-------------------------------------------------------------------------DNQET-----------------------------------------------------------------------HFSSIPDAFWWAVVTMTTVGYGDMRPVTVGGKIVG-SLCAIAGVLTIALPVPVIVSNFNYFYHR--------------------------------
62648178             --------------PFQRQVWLLFEYP-----------ESSGP----ARGIAI-VSVLVILISIVIFCLETLPQFRADGRGGSNEGSGTRMSPASR---------GSHEEEDEDEDS------------------------------------------------------------------------YAFPGSIP----------------------------------------------------------------------------------SGGLGTGGTSS-FSTLGG-------------------------------SFFTDPFFLVETLCIVWFTFELLVRFSAC-----------------PSKAAFFRNIM--------------------NIIDLVAIFPYFITLGTELVQR---------------------------HEQQPV-----------------SGGS------------------------------GQNGQQAMS---------------------LAILRVIRLVRVFRIFKLS-RHSKGL--------------------QILGKTLQA--------SMRELGLLIFFL----FIG--VILFSSAVYFA--EA-------------------------------------------------------------------------DDVDS-----------------------------------------------------------------------LFPSIPDAFWWAVVTMTTVGYGDMYPMTVGGKIVG-SLCAIAGVLTIALPVPVIVSNFNYFYHR--------------------------------
116435_151_481       --------------PFQRQVWLLFEYP-----------ESSGP----ARGIAI-VSVLVILISIVIFCLETLPQFRADGRGGSNEGSGTRMSPASR---------GSHEEEDEDEDS------------------------------------------------------------------------YAFPGSIP----------------------------------------------------------------------------------SGGLGTGGTSS-FSTLGG-------------------------------SFFTDPFFLVETLCIVWFTFELLVRFSAC-----------------PSKAAFFRNIM--------------------NIIDLVAIFPYFITLGTELVQR---------------------------HEQQPV-----------------SGGS------------------------------GQNRQQAMS---------------------LAILRVIRLVRVFRIFKLS-RHSKGL--------------------QILGKTLQA--------SMRELGLLIFFL----FIG--VILFSSAVYFA--EA-------------------------------------------------------------------------DDVDS-----------------------------------------------------------------------LFPSIPDAFWWAVVTMTTVGYGDMYPMTVGGKIVG-SLCAIAGVLTIALPVPVIVSNFNYFYHR--------------------------------
7305201_151_480      --------------PFQRQVWLLFEYP-----------ESSGP----ARGIAI-VSVLVILISIVIFCLETLPQFRADGRGGSNEGSGTRLSPASR----------SHEEEDEDEDS------------------------------------------------------------------------YAFPGSIP----------------------------------------------------------------------------------SGGLGTGGTSS-LSTLGG-------------------------------SFFTDPFFLVETLCIVWFTFELLVRFSAC-----------------PSKAAFFRNIM--------------------NIIDLVAIFPYFITLGTELVQR---------------------------HEQQSV-----------------SGGS------------------------------GQNGQQAMS---------------------LAILRVIRLVRVFRIFKLS-RHSKGL--------------------QILGKTLQA--------SMRELGLLIFFL----FIG--VILFSSAVYFA--EA-------------------------------------------------------------------------DDVDS-----------------------------------------------------------------------LFPSIPDAFWWAVVTMTTVGYGDMYPMTVGGKIVG-SLCAIAGVLTIALPVPVIVSNFNYFYHR--------------------------------
4504821_151_480      --------------PFQRQVWLLFEYP-----------ESSGP----ARGIAI-VSVLVILISIVIFCLETLPQFRVDGRGGNN-GGVSRVSPVSR---------GSQEEEEDEDDS------------------------------------------------------------------------YTFHHGIT----------------------------------------------------------------------------------PGEMGTGGSSS-LSTLGG-------------------------------SFFTDPFFLVETLCIVWFTFELLVRFSAC-----------------PSKPAFFRNIM--------------------NIIDLVAIFPYFITLGTELVQQ---------------------------QEQQPA-----------------SGG--G----------------------------GQNGQQAMS---------------------LAILRVIRLVRVFRIFKLS-RHSKGL--------------------QILGKTLQA--------SMRELGLLIFFL----FIG--VILFSSAVYFA--EA-------------------------------------------------------------------------DDDDS-----------------------------------------------------------------------LFPSIPDAFWWAVVTMTTVGYGDMYPMTVGGKIVG-SLCAIAGVLTIALPVPVIVSNFNYFYHR--------------------------------
114642947            --------------PFQRQVWLLFEYP-----------ESSGP----ARGIAI-VSVLVILISIVIFCLETLPQFRVDGRGGNN-GGVSRVSPVSR---------GSQEEEEEEDDS------------------------------------------------------------------------YTFHHGIT----------------------------------------------------------------------------------PGEMGTGGSSS-LSTLGG-------------------------------SFFTDPFFLVETLCIVWFTFELLVRFSAC-----------------PSKPAFFRNIM--------------------NIIDLVAIFHDFITLGTELVQQ---------------------------HEQQPA-----------------SGG--G----------------------------GQNGQQAMS---------------------LAILRVIRLVRVFRIFKLS-RHSKGL--------------------QILGKTLQA--------SMRELGLLIFFL----FIG--VILFSSAVYFA--EA-------------------------------------------------------------------------DDDDSL-----------------------------------------------------------------------FPSIPDAFWWAVVTMTTVGYGDMYPMTVGGKIVG-SLCAIAGVLTIALPVPVIVSNFNYFYHRETEQEEQGQYTHVT------------------
7800634_141_429      --------------EFQRQLWLLFEYP-----------ESSGP----ARMIAV-VSVSVIVISIVIFCLETLPQFR----------------------------------EDTSAN-------------------------------------------------------------------------LPLSNHHT---------------------------------------------------------------------------------------TNGTT--LHKK--------------------------------PNLFTDPFFMVETLCIVWFSFEFLVRFLSC-----------------PSKPAFFKNAM--------------------NSIDILAIAPYFITLGLELAEQ-----------------------------QEA------------------------------------------------------GSEQAMS---------------------LAILRVIRLVRVFRIFKLS-RHSKGL--------------------QILGQTLHA--------SISELGLLIFFL----LIG--VILFSSAVYFA--EA-------------------------------------------------------------------------DDPES-----------------------------------------------------------------------GFSSIPAAFWWAVVSMTTVGYGDMCPVTIGGKIVG-SMCAIAGVLTIALPVPVIVSNFNYFYHR--------------------------------
128485674_196_493    --------------AFARQLWLLFEFP-----------ESSQA----ARVLAV-VSVLVILVSIVVFCLETLPDFRDDRDDP------------------------GLAPVAAATG---------------------------------------------------------------------------SFLAR-----------------------------------------------------------------------------------------LNGSSPMPGAPP------------------------------RQPFNDPFFVVETLCICWFSFELLVHLVAC-----------------PSKAVFFKNVM--------------------NLIDFVAILPYFVALGTELARQ-----------------------------RGV-------------------------------------------------------GQPAMS---------------------LAILRVIRLVRVFRIFKLS-RHSKGL--------------------QILGQTLRA--------SMRELGLLIFFL----FIG--VVLFSSAVYFA--EV-------------------------------------------------------------------------DRVDT-----------------------------------------------------------------------HFTSIPESFWWAVVTMTTVGYGDMAPVTVGGKIVG-SLCAIAGVLTISLPVPVIVSNFSYFYHR--------------------------------
20828113_152_450     --------------AFARQLWLLFEFP-----------ESSQA----ARVLAV-VSVLVILVSIVVFCLETLPDFRDDRDDP------------------------GLAPVAAATG---------------------------------------------------------------------------PFLAR-----------------------------------------------------------------------------------------LNGSSPMPGAPP------------------------------RQPFNDPFFVVETLCICWFSFELLVRLVAC-----------------PSKAVFFKNVM--------------------NLIDFVAILPYFVALGTELARQ-----------------------------RGV-------------------------------------------------------GQPAMS---------------------LAILRVIRLVRVFRIFKLS-RHSKGL--------------------QILGQTLRA--------SMRELGLLIFFL----FIG--VVLFSSAVYFA--EV-------------------------------------------------------------------------DRVDT-----------------------------------------------------------------------HFTSIPESFWWAVVTMTTVGYGDMAPVTVGGKIVG-SLCAIAGVLTISLPVPVIVSNFSYFYHR--------------------------------
25952092_120_417     --------------AFARQLWLLFEFP-----------ESSQA----ARVLAV-VSVLVILVSIVVFCLETLPDFRDDRDGT------------------------GLA-AAAAAG---------------------------------------------------------------------------PFPAP-----------------------------------------------------------------------------------------LNGSSQMPGNPP------------------------------RLPFNDPFFVVETLCICWFSFELLVRLLVC-----------------PSKAIFFKNVM--------------------NLIDFVAILPYFVALGTELARQ-----------------------------RGV-------------------------------------------------------GQQAMS---------------------LAILRVIRLVRVFRIFKLS-RHSKGL--------------------QILGQTLRA--------SMRELGLLIFFL----FIG--VVLFSSAVYFA--EV-------------------------------------------------------------------------DRVDS-----------------------------------------------------------------------HFTSIPESFWWAVVTMTTVGYGDMAPVTVGGKIVG-SLCAIAGVLTISLPVPVIVSNFSYFYHR--------------------------------
13021998_143_430     --------------EFMKQVWLLFEHP-----------DSSSA----ARIIAI-ISVMVILISIVIFCLETLPEFRDDRDV------------------------------SLPVH---------------------------------------------------------------------------LHRAN-------------------------------------------------------------------------------------------STHHLKE---------------------------------KSPFEDPFFIVETICICWFSFELSVRFFAC-----------------PSKAAFFKDIM--------------------NMIDFVAIIPYFVALGTELARN-----------------------------KGV-------------------------------------------------------GQPAMS---------------------LAILRVIRLVRVFRIFKLS-RHSKGL--------------------QILGQTLKA--------SMRELGLLIFFL----FIG--VILFSSSVYFA--EA-------------------------------------------------------------------------DHADT-----------------------------------------------------------------------AFTSIPEAFWWAVVTMTTVGYGDMSPETVGGKLVG-SLCAIAGVLTISLPVPVIVSNFSYFYHR--------------------------------
125812539            --------------EFQRKIWMLFEHP-----------ESSGG----ARIIAI-ISVMVIVVSILIFCLETLPDFRVEKDFP-----------------------------SESSK--------------------------------------------------------------------MA---YEAHLAG------------------------------------------------------------------------------------------NNTSHISM---------------------------------SSPFHDPFFLVETMCICWFSFELIMRFTCS-----------------PNKMVFFKDVM--------------------NIIDFFAIIPYFVTLGTELAKS-----------------------------KG--------------------------------------------------------ATPSMS---------------------LAIVRVIRLVRVFRIFKLS-RHSKGL--------------------QILGQTLKA--------SLRELALLIFFL----FIG--VILFSSAAYFA--EV-------------------------------------------------------------------------DNPETA-----------------------------------------------------------------------FTSIPEAFWWAVVSMTTVGYGDMYPVTVGGKLVG-SMCAIAGVLTISLPVPVIVSNFSYFYHREMECEDNQEYTHVSTS----------------
4996280_173_483      --------------EPQRSIWLLFEYP-----------ESSAA----ARGIAI-MSVSVILLSIISFCLETLPAFERKNYFT-------------------------HLTATNASES-------------------------------------------------------------------------STIGNNPQ---------------------------------------------------------------------------------------GDITAVENSTAPIVLVT-------------------------YDMVNNPFWVIETICICWFSLEVTVRFLCC-----------------PSKCMFFKDMM--------------------NIIDFVAIAPYFITLATDLSKD-----------------------------NTS----------------------------------------------------DATSNKGMS---------------------LAILRVIRLVRVFRIFKLS-RHSKGL--------------------QILAQTLKA--------SMRELGLLIFFL----FIG--IILFSSAVYFA--EI-------------------------------------------------------------------------DEIETGR--------------------------------------------------------------------TQFTSIPEAFWWAVVTMTTVGYGDMKPITFGGKVVG-SLCAITGVLCIALPVPVIVSNFNYFYHR--------------------------------
3387822_187_452      --------------KTLKKIWLAMEYP-----------ETSAT----AYAVAF-ISVIMTTISIILFCVETLERFAKS-----------------------------------------------------------------------------------------------------------------------------------------------------------------------------------------------------------------HCVADEAP----------------------------------NFLDPFFIIETICTAWFTFEAIVRFISC-----------------PNKIMFWFGFQ--------------------NLIDVTAVVPYYVTLFNVVSTM-----------------------------SCS---------------------------------------------------------TAKS---------------------SASLAFLRVIRLVRVFKLT-KHSTGL--------------------QVLILTIKA--------SSEELGLFLVVL----LVC--MLVYSSAIYYA--EL-------------------------------------------------------------------------GVPKS-----------------------------------------------------------------------NIHSIPDAFWWAIITMTTVGYGDTFPVGPFGKVIG-AACAISGVLTLAMPVPILTGHFNKFYAH--------------------------------
3415132_1_247        ----------------------------------------STT----AYAVAF-ISVIMTTISIILFCVETLEKFSKS-----------------------------------------------------------------------------------------------------------------------------------------------------------------------------------------------------------------HCVADEAP----------------------------------NFLDPFFIIETICTAWFTFEAIVRFISC-----------------PNKIMFWFGFQ--------------------NLIDVTAVVPYYVTLFNVVSTM-----------------------------SCS---------------------------------------------------------TAKS---------------------SASLAFLRVIRLVRVFKVT-KHSTGL--------------------QVLILTIKA--------SSEELGLFLVVL----LVC--MLVYSSAIYYA--EL-------------------------------------------------------------------------GVPKS-----------------------------------------------------------------------DFHSIPDAFWWAIITMTTVGYGDTFPVGPFGKVIG-ATCAISGVLTLAMPVPILTGHFNKFYAH--------------------------------
32564068_176_455     --------------KTQRKIWELMEYP-----------DSSLS----ARIIAF-ISIAVIALSIISFCWETVPSDIEEKP------------------------------INNSAT---------------------------------------------------------------------------------------------------------------------------------------------------------------------------AELLDEMD---------------------------------EKHYSPFFWIELMCILWFTIELILRFISC-----------------PCKVTFATSVL--------------------NIIDFVAIAPFFVNFFFADTSK-----------------------------SNS----------------------------------------------------------SMS---------------------FAVLRVLRLVRVFRVFKLS-RHSVGL--------------------QILGKTFRS--------SVQEFCLLIFFM----AIA--LVLFASGMYFA--EQ-------------------------------------------------------------------------GEPNS-----------------------------------------------------------------------KFTSIPASFWFVLVTMTTVGYGDLVPLSPFGKVVG-GMCAMIGVLTLALPVPIIVANFKHFYRQ--------------------------------
987511_159_422       --------------PYLCKIWVLFEQP-----------SSSIY----AKIVAI-ISVLVILISIVVFCLETIPSLNPD----------------------------------KPEG--------------------------------------------------------------------------------------------------------------------------------------------------------------------------------------------------------------------SHMRVTWFIINTICNSWFTLEYLLRLIAS-----------------PNKLQFVKSTL--------------------NIVDLVSIIPYYVTATVGENDS-----------------------------------------------------------------------------------------GSI-----------------------AVLRVVRVIRVIRIFKLT-RHSRGL--------------------HILANTISA--------SLHELCMLVLFL----AIG--VVLFSSAAYYA--ES-------------------------------------------------------------------------ANSQT-----------------------------------------------------------------------GFKSIPHGFWWAVVTMTTVGYGDLFPSTYFGKMVG-ALCAISGVLVIALPVPVIVSNFEYYYKE--------------------------------
987509_151_421       --------------EPFKALWQFLENP-----------DSSNA----AKIFAI-LSVVIIVVSLIMFVIETLPVFAPKELL-----------------------------------------------------------------------------------------------------------------------------------------------------------------------------------------------------------TNNGTIYKTVY---------------------------------SEHATWMFTVNTAVICWFTIEFILRLICC-----------------PNKIKFFLNTG--------------------NIIDFLSILPYYLSLVLVSSSK-----------------------------------------------------------------------------------------GSF--------------------------SMLRVIRVLRVFKLS-RHSRGL--------------------QILGNTLKA--------SFNELMMLAFFL----FVM--ILIFGSCVYYA--EY-------------------------------------------------------------------------KEPGT-----------------------------------------------------------------------KFISIPSSFWWAIVTMTTVGYGDMHPVTFWGQIVG-SMAVVCGVLTIALPVPVVVSNFEYFYTK--------------------------------
103307_410_678       --------------EYQKYLWELLEKP-----------NTSFA----ARVIAV-ISILFIVLSTIALTLNTLPQLQ-HIDN-----------------------------------------------------------------------------------------------------------------------------------------------------------------------------------------------------------------------------------------------------GTPQDNPQLAMVEAVCITWFTLEYILRFSSS-----------------PDKWKFFKGGL--------------------NIIDLLAILPYFVSLFLLETNKNAT-----------------------------------------------------------------------------------DQFQDVR----------------------RVVQVFRIMRILRVLKLA-RHSTGL--------------------QSLGFTLRN--------SYKELGLLMLFL----AMG--VLIFSSLAYFA--EKDEK----------------------------------------------------------------------DTK--------------------------------------------------------------------------FVSIPEAFWWAGITMTTVGIRDICPTTALGKVIG-TVCCICGVLVVALPIPIIVNNFAEFYKN--------------------------------
158459_410_678       --------------EYQKYLWELLEKP-----------NTSFA----ARVIAV-ISILFIVLSTIALTLNTLPQLQ-HIDN-----------------------------------------------------------------------------------------------------------------------------------------------------------------------------------------------------------------------------------------------------GTPQDNPQLAMVEAVCITWFTLEYILRFSSS-----------------PDKWKFFKGGL--------------------NIIDLLAILPYFVSLFLLETNKNAT-----------------------------------------------------------------------------------DQFQDVR----------------------RVVQVFRIMRILRVLKLA-RHSTGL--------------------QSLGFTLRN--------SYKELGLLMLFL----AMG--VLIFSSLAYFA--EKDEK----------------------------------------------------------------------DTK--------------------------------------------------------------------------FVSIPEAFWWAGITMTTVGYGDICPTTALGKVIG-TVCCICGVLVVALPIPIIVNNFAEFYKN--------------------------------
24656289_410_678     --------------EYQKYLWELLEKP-----------NTSFA----ARVIAV-ISILFIVLSTIALTLNTLPQLQ-HIDN-----------------------------------------------------------------------------------------------------------------------------------------------------------------------------------------------------------------------------------------------------GTPQDNPQLAMVEAVCITWFTLEYILRFSAS-----------------PDKWKFFKGGL--------------------NIIDLLAILPYFVSLFLLETNKNAT-----------------------------------------------------------------------------------DQFQDVR----------------------RVVQVFRIMRILRVLKLA-RHSTGL--------------------QSLGFTLRN--------SYKELGLLMLFL----AMG--VLIFSSLAYFA--EKDEK----------------------------------------------------------------------DTK--------------------------------------------------------------------------FVSIPEAFWWAGITMTTVGYGDICPTTALGKVIG-TVCCICGVLVVALPIPIIVNNFAEFYKN--------------------------------
10727256_440_708     --------------EYQKYLWELLEKP-----------NTSFA----ARVIAV-ISILFIVLSTIALTLNTLPQLQ-HIDN-----------------------------------------------------------------------------------------------------------------------------------------------------------------------------------------------------------------------------------------------------GTPQDNPQLAMVEAVCITWFTLEYILRFSAS-----------------PDKWKFFKGGL--------------------NIIDLLAILPYFVSLFLLETNKNAT-----------------------------------------------------------------------------------DQFQDVR----------------------RVVQVFRIMRILRILKLA-RHSTGL--------------------QSLGFTLRN--------SYKELGLLMLFL----AMG--VLIFSSLAYFA--EKDEK----------------------------------------------------------------------DTK--------------------------------------------------------------------------FVSIPETFWWAGITMTTVGYGDIYPTTALGKVIG-TVCCICGVLVIALPIPIIVNNFAEFYKN--------------------------------
116126707_273_541    --------------QYQKYLWELLEKP-----------NTSFA----ARVIAV-ISILFIVLSTIALTLNTLPSLQ-SDHN-----------------------------------------------------------------------------------------------------------------------------------------------------------------------------------------------------------------------------------------------------GTSQDNPELAMVEAVCITWFTLEYVLRFSAS-----------------PDKWKFFKGGL--------------------NIIDLFAILPYFVSLFLLETNKNAT-----------------------------------------------------------------------------------DQFQDVR----------------------RVVQVFRIMRILRILKLA-RHSTGL--------------------QSLGFTLRN--------SYKELGLLMLFL----AMG--VLIFSSLAYFA--EKDEP----------------------------------------------------------------------QTK--------------------------------------------------------------------------FISIPETFWWAGITMTTVGYGDIYPTTPLGKVIG-TVCCICGVLVIALPIPIIVNNFAEFYKN--------------------------------
743110_290_559       --------------HWRQKVWDLLEKP-----------TTSMA----ARVVAI-ISILFIVLSTIALTLNTIPALQDRADP-----------------------------------------------------------------------------------------------------------------------------------------------------------------------------------------------------------------------------------------------------SLNQDNEDLAVVEAVCIGWFTLEYLARFWAS-----------------PNKWKFFKGPL--------------------NVIDLLAIMPYFISLGLTETNKSTT-----------------------------------------------------------------------------------EQFQNVR----------------------RVVQIFRIMRILRILKLA-RHSTGL--------------------QSLGYTLQR--------SYKELGMLLMFL----AIF--ILLFSSLAYFA--EKDEP----------------------------------------------------------------------ETK--------------------------------------------------------------------------YKSIPETFWWAAITMTTVGYGDIYPKTVLGKVVG-SVCCICGVLVIALPIPIIVNNFAEFYKD--------------------------------
2315214_356_624      --------------KWRQRVWDLLEKP-----------TTSMA----ARVLAI-VSILFVVLSTVALTLNTIPGLKGEGE------------------------------------------------------------------------------------------------------------------------------------------------------------------------------------------------------------------------------------------------------HEGADNPQLAIVEAVCIGWFTLEYLGRFWAS-----------------PNKWKFFKGPL--------------------NIIDLLAILPYFISLGLTETNKSTT-----------------------------------------------------------------------------------EQFQNVR----------------------RVVRVFRIMRVLRILKLA-RHSTGL--------------------QSLGYTLQR--------SYKELGLLMMFL----AIG--ILLFSSLAYFA--EKDEP----------------------------------------------------------------------GTK--------------------------------------------------------------------------YVSIPETFWWAAITMTTVGYGDICPTTILGKVVG-GVCCICGVLVVALPIPIIVNNFAEFYKD--------------------------------
15418950_168_436     --------------EKRKKLWDLLEKP-----------NSSVA----AKILAI-VSILFIVLSTIALSLNTLPELQETDEF-----------------------------------------------------------------------------------------------------------------------------------------------------------------------------------------------------------------------------------------------------GQPSDNPKLAHVEAVCIAWFTMEYLLRFLSS-----------------PNKWKFFKGPL--------------------NVIDLLAILPYYVTIFLTESNKSVL------------------------------------------------------------------------------------QFQNVR----------------------RVVQIFRIMRILRILKLA-RHSTGL--------------------QSLGFTLRR--------SYNELGLLILFL----AMG--IMIFSSLVFFA--EKDED----------------------------------------------------------------------ATK--------------------------------------------------------------------------FTSIPASFWWATITMTTVGYGDIYPKTLLGKIVG-GLCCIAGVLVIALPIPIIVNNFSEFYKE--------------------------------
16758912_168_436     --------------EKRKKLWDLLEKP-----------NSSVA----AKILAI-VSILFIVLSTIALSLNTLPELQENDEF-----------------------------------------------------------------------------------------------------------------------------------------------------------------------------------------------------------------------------------------------------GQPSDNRKLAHVEAVCIAWFTMEYLLRFLSS-----------------PNKWKFFKGPL--------------------NVIDLLAILPYYVTIFLTESNKSVL------------------------------------------------------------------------------------QFQNVR----------------------RVVQIFRIMRILRILKLA-RHSTGL--------------------QSLGFTLRR--------SYNELGLLILFL----AMG--IMIFSSLVFFA--EKDED----------------------------------------------------------------------ATK--------------------------------------------------------------------------FTSIPASFWWATITMTTVGYGDIYPKTLLGKIVG-GLCCIAGVLVIALPIPIIVNNFSEFYKE--------------------------------
27436974_168_436     --------------DKRKKLWDLLEKP-----------NSSVA----AKILAI-VSILFIVLSTIALSLNTLPELQETDEF-----------------------------------------------------------------------------------------------------------------------------------------------------------------------------------------------------------------------------------------------------GQLNDNRQLAHVEAVCIAWFTMEYLLRFLSS-----------------PNKWKFFKGPL--------------------NVIDLLAILPYYVTIFLTESNKSVL------------------------------------------------------------------------------------QFQNVR----------------------RVVQIFRIMRILRILKLA-RHSTGL--------------------QSLGFTLRR--------SYNELGLLILFL----AMG--IMIFSSLVFFA--EKDED----------------------------------------------------------------------ATK--------------------------------------------------------------------------FTSIPASFWWATITMTTVGYGDIYPKTLLGKIVG-GLCCIAGVLVIALPIPIIVNNFSEFYKE--------------------------------
17225492_1_241       ---------------------------------------------------AI-VSILFIVLSTIALSLNTLPELQEMDEF-----------------------------------------------------------------------------------------------------------------------------------------------------------------------------------------------------------------------------------------------------GQPNDNPQLAHVEAVCIAWFTMEYLLRFLSS-----------------PNKWKFFKGPL--------------------NVIDLLAILPYYVTIFLTESNKSVL------------------------------------------------------------------------------------QFQNVR----------------------RVVQIFRIMRILRILKLA-RHSTGL--------------------QSLGFTLRR--------SYNELGLLILFL----AMG--IMIFSSLVFFA--EKDED----------------------------------------------------------------------ATK--------------------------------------------------------------------------FTSIPASFWWATITMTTVGYGDIYPKTLLGKIVG-GLCCIAGVLVIALPIPIIVNNFSEFYKE--------------------------------
24418856_168_436     --------------EKRKKSRDLLEKP-----------NSSVA----AKILAI-VSNLFIVLSTIALSLNTLPELQEMDEF-----------------------------------------------------------------------------------------------------------------------------------------------------------------------------------------------------------------------------------------------------GQPNDNPQLAHVEAVCNAWFTMEYLLRFLSS-----------------PNKWKFFKGPL--------------------NVIDLLAILPYYVTIFLTESNKSVL------------------------------------------------------------------------------------QFQNVR----------------------RVVQIFRIMRILRILKLA-RHSTGL--------------------QSLGFTLRR--------SYNELGLLILFL----AMG--IMIFSSLVFFA--EKDAD----------------------------------------------------------------------ATK--------------------------------------------------------------------------FTSIPASFWWATITMTTVGYGDIYPKTLLGKSVG-GLCCIAGVLVIALPIPIIVPPFSEFYKE--------------------------------
1163141_168_436      --------------EKTKKLWDLLEKP-----------NSSVA----AKVLAI-ISILFIVLSTIALSLNTLPDLQEIDEF-----------------------------------------------------------------------------------------------------------------------------------------------------------------------------------------------------------------------------------------------------GQPNDNPQLAHVEAVCIAWFTMEYLLRFLSS-----------------PNKWKFFKGPL--------------------NIIDLLAILPYYVTIFLTASNKSVL------------------------------------------------------------------------------------QFQNVR----------------------RVVQIFRIMRILRILKLA-RHSTGL--------------------QSLGFTLRR--------SYNELGLLILFL----AMG--IMIFSSLVFFA--EKDED----------------------------------------------------------------------ATK--------------------------------------------------------------------------FTSIPASFWWATITMTTVGYGDIYPKTLLGKIIG-GLCCIAGVLVIALPIPIIVNNFSEFYKE--------------------------------
13195252_169_437     --------------DKRKKLWDLLEKP-----------SSSVA----AKILAI-ISILFIILSTIALSLNTLPELQVIDEF-----------------------------------------------------------------------------------------------------------------------------------------------------------------------------------------------------------------------------------------------------GQANDNPQLAHIEAVCIAWFTMEYLLRFLSS-----------------PNKWKFFKGPL--------------------NVIDLLAILPYYVTIFLTESNKSVL------------------------------------------------------------------------------------QFQNVR----------------------RVVQIFRIMRILRILKLA-RHSTGL--------------------QSLGFTLRR--------SYNELGLLILFL----AMG--IMIFSSLVFFA--EKDED----------------------------------------------------------------------ATK--------------------------------------------------------------------------FTSIPASFWWATITMTTVGYGDIYPQTLLGKIVG-GLCCIAGVLVIALPIPIIVNNFSEFYKE--------------------------------
6981120              --------------EKRKKLWDLLEKP-----------NSSVA----AKILAI-ISIMFIVLSTIALSLNTLPELQSL---------------------------------------------------------------------------------------------------------------------------------------------------------------------------------------------------------------------------------------------D--------EFGQSTDNPQLAHVEAVCIAWFTMEYLLRFLSS-----------------PKKWKFFKGPL--------------------NAIDLLAILPYYVTIFLTESNKS------------------------------------------------------------------------------------VLQFQNVR----------------------RVVQIFRIMRILRILKLA-RHSTGL--------------------QSLGFTLRR--------SYNELGLLILFL----AMG--IMIFSSLVFFA--EKDED----------------------------------------------------------------------DTK--------------------------------------------------------------------------FKSIPASFWWATITMTTVGYGDIYPKTLLGKIVG-GLCCIAGVLVIALPIPIIVNNFSEFYKEQKRQEKAIKRREALER----------------
47523520             --------------EKRKKLWDLLEKP-----------NSSVA----AKILAI-ISIMFIVLSTIALSLNTLPELQSL---------------------------------------------------------------------------------------------------------------------------------------------------------------------------------------------------------------------------------------------D--------EFGQTTDNPQLAHVEAVCIAWFTMEYLLRFLSS-----------------PKKWKFFKGPL--------------------NAIDLLAILPYYVTIFLTESNKS------------------------------------------------------------------------------------VLQFQNVR----------------------RVVQIFRIMRILRILKLA-RHSTGL--------------------QSLGFTLRR--------SYNELGLLILFL----AMG--IMIFSSLVFFA--EKDED----------------------------------------------------------------------DTK--------------------------------------------------------------------------FKSIPASFWWATITMTTVGYGDIYPKTLLGKIVG-GLCCIAGVLVIALPIPIIVNNFSEFYKEQKRQEKAIKRREALER----------------
1163143_164_432      --------------EKTKKLWDLLEKP-----------NSSVA----AKILAI-ISILFIVLSTIALSLNTLPDLQAIDEF-----------------------------------------------------------------------------------------------------------------------------------------------------------------------------------------------------------------------------------------------------GQTTDNAQLAHVEAVCIAWFTMEYLLRFLSS-----------------PNKWKFFKGPL--------------------NVIDLLAILPYYVTIFLTESNKSVL------------------------------------------------------------------------------------QFQNVR----------------------RVVQIFRIMRILRILKLA-RHSTGL--------------------QSLGFTLRR--------SYNELGLLILFL----AMG--IMIFSSLVFFA--EKDED----------------------------------------------------------------------DTK--------------------------------------------------------------------------FKSIPASFWWATITMTTVGYGDIYPKTLLGKIVG-GLCCIAGVLVIALPIPIIVNNFSEFYKE--------------------------------
4519932_203_467      --------------QKRKKLWDLLEKP-----------GTSLA----AKVLAI-ISILFIVLSTVALSLNTLPDLRGEN---------------------------------------------------------------------------------------------------------------------------------------------------------------------------------------------------------------------------------------------------------DKDNEDLAHVESVCIAWFTMEYLLRLVSA-----------------PNKWKFFKGPL--------------------NIIDLLAVLPYYVTVFLTESNSQIL------------------------------------------------------------------------------------QFQNVR----------------------RVVQIFRIMRIMRILKLA-RHSTGL--------------------QSLGYTLRR--------SYNELGLLMLFL----AMG--IMVFSSLAYFA--EKMEN------------------------------------------------------------------------------------------------------------------------------------------------AEMFNSIPASFWWATITMTTVGYGDIYPKTVLGKLVG-ATCCITGVLVIALPIPIIVNNFSEFYKE--------------------------------
20070166_157_425     --------------EKTKKLWDLLEKP-----------ESSCP----ARVVAV-LSFLLILVSSVVMCMGTIPELQVLDAE-----------------------------------------------------------------------------------------------------------------------------------------------------------------------------------------------------------------------------------------------------GNRVEHPTLENVETACIGWFTLEYLLRLFSS-----------------PNKLHFALSFM--------------------NIVDVLAILPFYVSLTLTHLGARMM------------------------------------------------------------------------------------ELTNVQ----------------------QAVQALRIMRIARIFKLA-RHSSGL--------------------QTLTYALKR--------SFKELGLLLMYL----AVG--IFVFSALGYTM--EQSHP----------------------------------------------------------------------ETL--------------------------------------------------------------------------FKSIPQSFWWAIITMTTVGYGDIYPKTTLGKLNA-AISFLCGVIAIALPIHPIINNFVRYYNK--------------------------------
7513252_157_425      -----------------------LEKP-----------ESSCP----ARVVAE-LSFLLILVSSVVMCMDTIPELQVLDAE-----------------------------------------------------------------------------------------------------------------------------------------------------------------------------------------------------------------------------------------------------GNRVEHPTLENVETACIGWFTLEYLLRLFSS-----------------PNKLHFALSFM--------------------NIVDVLAILPFYVSLTLTHLGARMM------------------------------------------------------------------------------------ELTNVQ----------------------QAVQALRIMRIARIFKLA-RHSSGL--------------------QTLTYALKR--------SFKELGLLLMYL----AVG--IFVFSALGYTM--EQSHP----------------------------------------------------------------------ETL--------------------------------------------------------------------------FKNIPQSFWWAIITMTTVGYGDIYPKTTLSKLNA-AISFLCGVIAIALPIHPIINNFVRYYNK--------------------------------
20845975_157_425     --------------RCQKCVWKFLEKP-----------ESSCP----ARVVAV-LSFLLILVSSVVMCMGTIPELQVVDSE-----------------------------------------------------------------------------------------------------------------------------------------------------------------------------------------------------------------------------------------------------GNRVEHPTLENVETACIGWFTLEYLLRLFSS-----------------PNKLHFALSFM--------------------NIVDVLAILPFYVSLTLTHLGARMM------------------------------------------------------------------------------------ELTNVQ----------------------QAVQALRIMRIARIFKLA-RHSSGL--------------------QTLTYALKR--------SFKELGLLLMYL----AVG--IFVFSALGYTM--EQSHP----------------------------------------------------------------------ETL--------------------------------------------------------------------------FKSIPQSFWWAIITMTTVGYGDIYPKTTLGKLNA-AISFLCGVIAIALPIHPIINNFVRYYNK--------------------------------
13027418_163_429     --------------NFRRQLWLALDNP-----------GYSVL----SRVFSV-LSILVVLGSIITMCLNSLPDFQIPDSQ-------------------------------------------------------------------------------------------------------------------------------------------------------------------------------------------------------------------------------------------------GNPGE----DPRFEIVEHFGIAWFTFELVARFAVA-----------------PDFLKFFKNAL--------------------NLIDLMSIVPFYITLVVNLVVESSP------------------------------------------------------------------------------------TLANLG----------------------RVAQVLRLMRIFRILKLA-RHSTGL--------------------RSLGATLKY--------SYKEVGLLLLYL----SVG--ISIFSVVAYTI--EKEEN-----------------------------------------------------------------------EG--------------------------------------------------------------------------LATIPACWWWATVSMTTVGYGDVVPGTTAGKLTA-SACILAGILVVVLPITLIFNKFSHFYRR--------------------------------
6329973_175_441      --------------NFRRQLWLALDNP-----------GYSVL----SRVFSI-LSILVVMGSIITMCLNSLPDFQIPDSQ-------------------------------------------------------------------------------------------------------------------------------------------------------------------------------------------------------------------------------------------------GNPGE----DPRFEIVEHFGIAWFTFELVARFAVA-----------------PDFLKFFKNAL--------------------NLIDLMSIVPFYITLVVNLVVESTP------------------------------------------------------------------------------------TLANLG----------------------RVAQVLRLMRIFRILKLA-RHSTGL--------------------RSLGATLKY--------SYKEVGLLLLYL----SVG--ISIFSVVAYTI--EKEEN-----------------------------------------------------------------------EG--------------------------------------------------------------------------LATIPACWWWATVSMTTVGYGDVVPGTTAGKLTA-SACILAGILVVVLPITLIFNKFSHFYRR--------------------------------
7514118_165_447      --------------RLRRRLWLTMENP-----------GYSLP----SKLFSC-VSIGVVLASIAAMCIHSLPEYQAREAAAA-----------------------------------------------------------------------------------------------------------------------------------------------------------------------------------------------------------------------------------------VAAVAAGRSAEDVRDDPVLRRLEYFCIAWFSFEVSSRLLLA-----------------PSTRNFFCHPL--------------------NLIDIVSVLPFYLTLLAGAALGDRRGAS-------------------------------------------------------------------------------GEELGDLG----------------------KVVQVFRLMRIFRVLKLA-RHSTGL--------------------RSLGATLKH--------SYREVGILLLYL----AVG--VSVFSGVAYTA--EERN------------------------------------------------------------------------VG--------------------------------------------------------------------------FDTIPACWWWGTVSMTTVGYGDVVPETVAGKLAA-SGCILGGILVVALPITIIFNKFSHFYRR--------------------------------
16758838_165_447     --------------RLRRRLWLTMENP-----------GYSLP----SKLFSC-VSIGVVLASIAAMCIHSLPEYQAREAAAA-----------------------------------------------------------------------------------------------------------------------------------------------------------------------------------------------------------------------------------------VAAVAAGRSAEDVRDDPVLRRLEYFCIAWFSFEVSSRLLLA-----------------PSTRNFFCHPL--------------------NLIDIVSVLPFYLTLLAGAALGDRRGAS-------------------------------------------------------------------------------GEELGDLG----------------------KVVQVFRLMRIFRVLKLA-RHSTGL--------------------RSLGATLKH--------SYREVGILLLYL----AVG--VSVFSGVAYTA--EEKN------------------------------------------------------------------------VG--------------------------------------------------------------------------FDTIPACWWWGTVSMTTVGYGDVVPETVAGKLAA-SGCILGGILVVALPITIIFNKFSHFYRR--------------------------------
112821679_165_447    --------------RLRRRLWLTMENP-----------GYSLP----SKLFSC-VSIGVVLASIAAMCIHSLPEYQAREAAAA-----------------------------------------------------------------------------------------------------------------------------------------------------------------------------------------------------------------------------------------VAAVAAGRSAEEVRDDPVLRRLEYFCIAWFSFEVSSRLLLA-----------------PSTRNFFCHPL--------------------NLIDIVSVLPFYLTLLAGAALGDQRGAS-------------------------------------------------------------------------------GEELGDLG----------------------KVVQVFRLMRIFRVLKLA-RHSTGL--------------------RSLGATLKH--------SYREVGILLLYL----AVG--VSVFSGVAYTA--EEEN------------------------------------------------------------------------EG--------------------------------------------------------------------------FHTIPACWWWGTVSMTTVGYGDVVPETVGGKLAA-SGCILGGILVVALPITIIFNKFSHFYRR--------------------------------
13492973_196_476     --------------RLRRRLWLTMENP-----------GYSLP----SKLFSC-VSISVVLASIAAMCIHSLPEYQAREAAAA-----------------------------------------------------------------------------------------------------------------------------------------------------------------------------------------------------------------------------------------VAAVAAGRSPEGVRDDPVLRRLEYFCIAWFSFEVSSRLLLA-----------------PSTRNFFCHPL--------------------NLIDIVSVLPFYLTLLAGVALGDQG----------------------------------------------------------------------------------GKEFGHLG----------------------KVVQVFRLMRIFRVLKLA-RHSTGL--------------------RSLGATLKH--------SYREVGILLLYL----AVG--VSVFSGVAYTA--EKEED-----------------------------------------------------------------------VG--------------------------------------------------------------------------FNTIPACWWWGTVSMTTVGYGDVVPVTVAGKLAA-SGCILGGILVVALPITIIFNKFSHFYRR--------------------------------
13278747_161_425     --------------QLRKKIWIRMENP-----------AYCLS----AKLIAI-SSLSVVLASIVAMCVHSMSEFQNEDGE--------------------------------------------------------------------------------------------------------------------------------------------------------------------------------------------------------------------------------------------------------VDDPVLEGLEIACIAWFTGELAVRLAAA-----------------PCQKKFWKNPL--------------------NIIDFVSIIPFYATLAVDTKEEESE------------------------------------------------------------------------------------DIENMG----------------------KVVQILRLMRIFRILKLA-RHSVGL--------------------RSLGATLRH--------SYHEVGLLLLFL----SVG--ISIFSVLIYSV--EKDDH-----------------------------------------------------------------------TSS-------------------------------------------------------------------------LTSIPICWWWATISMTTVGYGDTHPVTLAGKLIA-STCIICGILVVALPITIIFNKFSKYYQK--------------------------------
25952108_161_425     --------------QLRKKIWIRMENP-----------AYCLS----AKLIAI-SSLSVVLASIVAMCVHSMSEFQNEDGE--------------------------------------------------------------------------------------------------------------------------------------------------------------------------------------------------------------------------------------------------------VDDPVLEGVEIACIAWFTGELAVRLAAA-----------------PCQKKFWKNPL--------------------NIIDFVSIIPFYATLAVDTKEEESE------------------------------------------------------------------------------------DIENMG----------------------KVVQILRLMRIFRILKLA-RHSVGL--------------------RSLGATLRH--------SYHEVGLLLLFL----SVG--ISIFSVLIYSV--EKDDH-----------------------------------------------------------------------TSS-------------------------------------------------------------------------LTSIPICWWWATISMTTVGYGDTHPVTLAGKLIA-STCIICGILVVALPITIIFNKFSKYYQK--------------------------------
6653657_161_425      --------------QLRKKIWIRMENP-----------AYCLS----AKLIAI-SSLSVVLASIVAMCVHSMSEFQNEDGE--------------------------------------------------------------------------------------------------------------------------------------------------------------------------------------------------------------------------------------------------------VDDPVLEGVEIACIAWFTGELAVRLVAA-----------------PCQKKFWKNPL--------------------NIIDFVSIIPFYATLAVDTKEEESE------------------------------------------------------------------------------------DIENMG----------------------KVVQILRLMRIFRILKLA-RHSVGL--------------------RSLGATLRH--------SYHEVGLLLLFL----SVG--ISIFSVLIYSV--EKDDH-----------------------------------------------------------------------TSS-------------------------------------------------------------------------LTSIPICWWWATISMTTVGYGDTHPVTLAGKLIA-STCIICGILVVALPITIIFNKFSKYYQK--------------------------------
3559986_161_425      --------------QLRKKIWIRMENP-----------AYCLS----AKLIAI-SSLSVVLASIVAMCVHSMSEFQNEDGE--------------------------------------------------------------------------------------------------------------------------------------------------------------------------------------------------------------------------------------------------------VDDPVLEGVEIACIAWFTGELAIRLVAA-----------------PSQKKFWKNPL--------------------NIIDFVSIIPFYATLAVDTKEEESE------------------------------------------------------------------------------------DIENMG----------------------KVVQILRLMRIFRILKLA-RHSVGL--------------------RSLGATLRH--------SYHEVGLLLLFL----SVG--ISIFSVLIYSV--EKDEL-----------------------------------------------------------------------ASS-------------------------------------------------------------------------LTSIPICWWWATISMTTVGYGDTHPVTLAGKIIA-STCIICGILVVALPITIIFNKFSKYYQK--------------------------------
20845705_254_518     --------------QLRKKIWIRMENP-----------AYCLS----AKLIAI-SSLSVVLASIVAMCVHSMSEFQNEDGE--------------------------------------------------------------------------------------------------------------------------------------------------------------------------------------------------------------------------------------------------------VDDPVLEGVEIACIAWFTGELAIRLVAA-----------------PSQKKFWKNPL--------------------NIIDFVSIIPFYATLAVDTKEEESE------------------------------------------------------------------------------------DIENMG----------------------KVVQILRLMRIFRILKLA-RHSVGL--------------------RSLGATLRH--------SYHEVGLLLLFL----SVG--ISIFSVLIYSV--EKDEH-----------------------------------------------------------------------KSS-------------------------------------------------------------------------LTSIPICWWWATISMTTVGYGDTHPVTLAGKIIA-STCIICGILVVALPITIIFNKFSKYYQK--------------------------------
19424136_235_512     --------------PQRRRLWNLMEKP-----------FSSVA----AKAIGV-ASSTFVLVSVVALALNTVEEMQQHSGQ---------------------------------------------------------------------------------------------------------------------------------------------------------------------------------------------------------------------------------------------------GEGGPDLRPILEHVEMLCMGFFTLEYLLRLAST-----------------PDLRRFARSAL--------------------NLVDLVAILPLYLQLLLECFTGEGHQRG--------------------------------------------------------------------------------QTVGSVGKVG-------------------QVLRVMRLMRIFRILKLA-RHSTGL--------------------RAFGFTLRQ--------CYQQVGCLLLFI----AMG--IFTFSAAVYSV--EHDVP----------------------------------------------------------------------STN--------------------------------------------------------------------------FTTIPHSWWWAAVSISTVGYGDMYPETHLGRFFA-FLCIAFGIILNGMPISILYNKFSDYYSK--------------------------------
20887749_243_529     --------------PQRQRLWNLMEKP-----------FSSVA----AKAMGV-ATNLFVLISVVALALNTVEEMQHQAEQ---------------------------------------------------------------------------------------------------------------------------------------------------------------------------------------------------------------------------------------------------GTGGGDPRPILEHVEMLCVAFFTLEFLLRLAST-----------------PNLQRFARSAL--------------------NLVDLVAILPFYLQLLLECFTSEDQRHNK-----------------------------------------------------------------------DSPREHDLETVGRVGKVG-------------------QVLRIMRLMRIFRILKLA-RHSTGL--------------------RAFGFTLRQ--------CYQQVGCLMLFI----TMG--IFSFSAAVYSV--EHDVP----------------------------------------------------------------------GTN--------------------------------------------------------------------------FTSILHAWWWAAVSISTVGYGDMYPETHLGRLFA-FLCIAFGIILNGMPISILYNKFSDYYSK--------------------------------
11067433_189_450     --------------TVRQKLWDILEKP-----------GSSTA----ARIFGV-ISIIFVAVSIVNMALMS-AELSWLNLQ------------------------------------------------------------------------------------------------------------------------------------------------------------------------------------------------------------------------------------------------------------LLEILEYVCISWFTGEFILRFLCV-----------------KDRCHFLRKVP--------------------NIIDLLAILPFYITLLVESLSGSHT----------------------------------------------------------------------------------TQELENVG----------------------RLVQVLRLLRALRMLKLG-RHSTGL--------------------RSLGMTITQ--------CYEEVGLLLLFL----SVG--ISIFSTIEYFA--EQSIP----------------------------------------------------------------------DTT--------------------------------------------------------------------------FTSVPCAWWWATTSMTTVGYGDIRPDTTTGKIVA-FMCILSGILVLALPIAIINDRFSACYFT--------------------------------
20902063_189_450     --------------TVRQKLWDILEKP-----------GSSTA----ARIFGV-ISIIFVAVSIVNMALMS-AELSWLNLQ------------------------------------------------------------------------------------------------------------------------------------------------------------------------------------------------------------------------------------------------------------LLEILEYVCISWFTGEFVLRFLCV-----------------KDRCHFLRKVP--------------------NIIDLLAILPFYITLLVESLSGSHT----------------------------------------------------------------------------------TQELENVG----------------------RLVQVLRLLRALRMLKLG-RHSTGL--------------------RSLGMTITQ--------CYEEVGLLLLFL----SVG--ISIFSTIEYFA--EQSIP----------------------------------------------------------------------DTT--------------------------------------------------------------------------FTSVPCAWWWATTSMTTVGYGDIRPDTTTGKIVA-FMCILSGILVLALPIAIINDRFSACYFT--------------------------------
1438971_190_451      --------------TVRQKLWDILEKP-----------GSSTA----ARIFGV-ISIIFVAVSIVNMALMS-AELSWLNLQ------------------------------------------------------------------------------------------------------------------------------------------------------------------------------------------------------------------------------------------------------------LLEILEYVCISWFTGEFILRFLCV-----------------KDRCRFLRKVP--------------------NIIDLLAILPFYITLLVESLSGSHT----------------------------------------------------------------------------------TQELENVG----------------------RLVQVLRLLRALRMLKLG-RHSTGL--------------------RSLGMTITQ--------CYEEVGLLLLFL----SVG--ISIFSTIEYFA--EQSIP----------------------------------------------------------------------DTT--------------------------------------------------------------------------FTSVPCAWWWATTSMTTVGYGDIRPDTTTGKIVA-FMCILSGILVLALPIAIINDRFSACYFT--------------------------------
20381121_186_447     --------------TVRQKLWNILEKP-----------GSSTA----ARIFGV-ISIIFVVVSIINMALMS-AELSWLDLQ------------------------------------------------------------------------------------------------------------------------------------------------------------------------------------------------------------------------------------------------------------LLEILEYVCISWFTGEFVLRFLCV-----------------RDRCRFLRKVP--------------------NIIDLLAILPFYITLLVESLSGSQT----------------------------------------------------------------------------------TQELENVG----------------------RIVQVLRLLRALRMLKLG-RHSTGL--------------------RSLGMTITQ--------CYEEVGLLLLFL----SVG--ISIFSTVEYFA--EQSIP----------------------------------------------------------------------DTT--------------------------------------------------------------------------FTSVPCAWWWATTSMTTVGYGDIRPDTTTGKIVA-FMCILSGILVLALPIAIINDRFSACYFT--------------------------------
17559262_220_512     --------------KLRRRMWTFLERP-----------GSSMQ----AKAFEL-SSTLFVAISVMGLSFGTIPDFQVT------HLMPPHNETVVL---------------PNG----------------------------------------------------------------------------------------------------------------------------------------------------------------------------TVTVVQK------------------------VEQMR-------VEHPAFVFTERICIAFFTVEYCLRFFAA-----------------PRKLRFALKPL--------------------NLVDLLAIVPFYLELLLTLCGVDDRK---------------------------------------------------------------------------------LRDLRWAF----------------------LVVRILRVLRVIRIIKLG-RFSSGL--------------------QTFGMTLQR--------SQKQLQMMTIVL----LTG--VVFFSTMIYFL--EKDEE----------------------------------------------------------------------GTP--------------------------------------------------------------------------FTSIPAAYWWCIVTMTTVGYGDAVPATTMGKIIA-SAAIMCGVLVLALPITIIVDNFIKVAQD--------------------------------
16604197_191_516     --------------NIRRRLWLILEGH-----------SQSKW----WKAFEI-ISTAFVVLSISALILGSIPEFQVPQKSEDGQLVYATATYPTY---------------PNGGGMT----------------------------------------------------------------------------------------------------------------------------------------------------------------------IESRTVVREGSDNSTGDLMAEE----------TVPTPRNTGKIEMTEHPVFTWVENVCVIYFSIEYAMRFIVA-----------------PRKLAFARQIL--------------------NVIDLLSIAPFYFELLLWICGISGEN---------------------------------------------------------------------------------VRKVRWAF----------------------LTVRLLRVLRVIRIAKLG-RFSPGL--------------------ANFALTIRK--------SKKQMQMVGVVM----MTV--IIFFSTLIYFL--ERDEP----------------------------------------------------------------------GTK--------------------------------------------------------------------------FTSIPATFWWCVVTMATVGYGDLVPVTVAGKLVG-SGAIVCGVMVLALPITIMVNNFMQVVKL--------------------------------
32564679_192_472     --------------EIRRCVWNIIEEP-----------ASSGK----AQAFAV-CSVVFVLISISGLVLGSLPELQVA---------------TKQ---------------RN-----------------------------------------------------------------------------------------------------------------------------------------------------------------------------NLTGEEF------------------------TEMEP---------MPILGYIEYVCIVWFTMEYGLKMLVS-----------------AERSKTFRQLL--------------------NIIDLLAILPFIIEMLLLIFGISTEQ---------------------------------------------------------------------------------LRDLKGAF----------------------LVIRILRVLRVIRVLKLG-RYSSGL--------------------QMFGKTLKA--------SFRQLGMMAMVV----MTG--VIFFSTLVYFL--EKDEP----------------------------------------------------------------------ASK--------------------------------------------------------------------------FHSIPAACWWCIVTMTTVGYGDLTPVTVPGKLVA-TGAIACGVLVLALPITIIVDNFMKVAET--------------------------------
25147276_203_482     -------------RSMRSRIHQFCEG------------DGSLF----STLFTF-GSITFVLISVIGLVLGSIHEFQVP---------------IYK---------------KGGKSER----------------------------------------------------------------------------------------------------------------------------------------------------------------------ISNWSIVENEN----------------------IIWEP---------HPIFGYVETVCIIWFTVEFIVRLAVT-----------------PNRFQFLVGIM--------------------NIVDMIAIIPFYLELGLALFGIDVAS---------------------------------------------------------------------------------LSDIKGAL----------------------LVVRVLRVLRVVRILKLG-RYSSGM--------------------RTFALTLKS--------SARQLGMMGMVL----STG--VVFFSTLLYFV--EKDEK----------------------------------------------------------------------DTP--------------------------------------------------------------------------FTSIPAAFWWAIVTMTTVGYGDYVPVTVPGKLIA-SGAIISGVLVLALPITIIVDNFMKVSGK--------------------------------
71985535_157_465     --------------GPRLAMWKFLEDP-----------QSSVF----AAVFAL-LSVFFVFASVVGLILGSMPEFQADSSN---AATYHVMHVRSR---------------PNDYGNK---------------------------------------------------------------------------------------------------------------------------------------------------------------------FGNDDDVAPNELLK-------------------DFVYKPTD-----SPNLPLTILEYICIGWFTFEYLVRFTIY-----------------PRKRQFVKKTL--------------------NIIDLSTILPFYLEICLPLFGVESR----------------------------------------------------------------------------------LKEFTGAM----------------------LVVRVLRVLRMARVFKLA-RYSTSL--------------------QTFGHTLQS--------SITELSMLSMFL----ITG--IVFFSTIMYYL--EKDEP----------------------------------------------------------------------HTD--------------------------------------------------------------------------FYSIPAGCWWCVVTMATVGYGDAKPVTTLGKLVA-TSTSICGIIVLAFPISMIVEKFATAQQR--------------------------------
22164082_146_417     --------------RWLERMRRTFEEP-----------TSSLA----AQILAS-VSVVFVIVSMVVLCASTLPDWRNAAADNRSLDDRSR------------------------------------------------------------------------------------------------------------------------------------------------------------------------------------------------------------------------------------------------------IIEAICIGWFTAECIVRFIVS-----------------KNKCEFVKRPL--------------------NIIDLLAITPYYISVLMTVFTGENS------------------------------------------------------------------------------------QLQRAG----------------------VTLRVLRMMRIFWVIKLA-RHFIGL--------------------QTLGLTLKR--------CYREMVMLLVFI----CVA--MAIFSALSQLL--EHGLDLE--------------------------------------------------------------------TSNKD------------------------------------------------------------------------FTSIPAACWWVIISMTTVGYGDMYPITVPGRILG-GVCVVSGIVLLALPITFIYHSFVQCYHE--------------------------------
19071574_146_428     --------------RWLERMRRTFEEP-----------TSSLA----AQILAS-VSVVFVIVSMVVLCASTLPDWRNAAADNRSLDDRSRYSAG-------------------------------------------------------------------------------------------------------------------------------------------------------------------------------------------------------------------------------------------PGREPSGIIEAICIGWFTAECIVRFIVS-----------------KNKCEFVKRPL--------------------NIIDLLAITPYYISVLMTVFTGENS------------------------------------------------------------------------------------QLQRAG----------------------VTLRVLRMMRIFWVIKLA-RHFIGL--------------------QTLGLTLKR--------CYREMVMLLVFI----CVA--MAIFSALSQLL--EHGLDLE--------------------------------------------------------------------TSNKD------------------------------------------------------------------------FTSIPAACWWVIISMTTVGYGDMYPITVPGRILG-GVCVVSGIVLLALPITFIYHSFVQCYHE--------------------------------
22164088_143_425     --------------RWLERMRRTFEEP-----------TSSLA----AQILAS-VSVVFVIVSMVVLCASTLPDWRAAAADNRSLDDRSRYSAS-------------------------------------------------------------------------------------------------------------------------------------------------------------------------------------------------------------------------------------------PGREPSGIIEAICIGWFTAECIVRFIVS-----------------KNKCEFVKRPL--------------------NIIDLLAITPYYISVLMTVFTGENS------------------------------------------------------------------------------------QLQRAG----------------------VTLRVLRMMRIFWVIKLA-RHFIGL--------------------QTLGLTLKR--------CYREMVMLLVFI----CVA--MAIFSALSQLL--EHGLDLE--------------------------------------------------------------------TSNKD------------------------------------------------------------------------FASIPAACWWVIISMTTVGYGDMYPITVPGRILG-GVCVVSGIVLLALPITFIYHSFVQCYHE--------------------------------
22164086_143_414     --------------RWLERMRRTFEEP-----------TSSLA----AQILAS-VSVVFVIVSMVVLCASTLPDWRAAAADNRSLDDRSR------------------------------------------------------------------------------------------------------------------------------------------------------------------------------------------------------------------------------------------------------IIEAICIGWFTAECIVRFIVS-----------------KNKCEFVKRPL--------------------NIIDLLAITPYYISVLMTVFTGENS------------------------------------------------------------------------------------QLQRAG----------------------VTLRVLRMMRIFWVIKLA-RHFIGL--------------------QTLGLTLKR--------CYREMVMLLVFI----CVA--MAIFSALSQLL--EHGLDLE--------------------------------------------------------------------TSNKD------------------------------------------------------------------------FASIPAACWWVIISMTTVGYGDMYPITVPGRILG-GVCVVSGIVLLALPITFIYHSFVQCYHE--------------------------------
22164090_143_414     --------------RWLERMRRTFEEP-----------TSSLA----AQILAS-VSVVFVIVSMVVLCASTLPDWRAAVADNRSLDDRSR------------------------------------------------------------------------------------------------------------------------------------------------------------------------------------------------------------------------------------------------------IIEAICIGWFTAECIVRFIVS-----------------KNKCEFVKRPL--------------------NIIDLLAITPYYISVLMTVFTGENS------------------------------------------------------------------------------------QLQRAG----------------------VTLRVLRMMRIFWVIKLA-RHFIGL--------------------QTLGLTLKR--------CYREMAMLLVFI----CVA--MAIFSALSQLL--EHGLDLE--------------------------------------------------------------------TSNKD------------------------------------------------------------------------FASIPAACWWVIISMTTVGYGDMYPITVPGRILG-GVCVVSGIVLLALPITFIYHSFVQCYHE--------------------------------
23595565_143_425     --------------RWLERMRRTFEEP-----------TSSLA----AQILAS-VSVVFVIVSMVVLCASTLPDWRAAVADNRSLDDRSRYSAS-------------------------------------------------------------------------------------------------------------------------------------------------------------------------------------------------------------------------------------------PGREPSGIIEAICIGWFTAECIVRFIVS-----------------KNKCEFVKRPL--------------------NIIDLLAITPYYISVLMTVFTGENS------------------------------------------------------------------------------------QLQRAG----------------------VTLRVLRMMRIFWVIKLA-RHFIGL--------------------QTLGLTLKR--------CYREMAMLLVFI----CVA--MAIFSALSQLL--EHGLDLE--------------------------------------------------------------------TSNKD------------------------------------------------------------------------FASIPAACWWVIISMTTVGYGDMYPITVPGRILG-GVCVVSGIVLLALPITFIYHSFVQCYHE--------------------------------
6006605_166_437      --------------RGRRRLRDVVENP-----------HSGLA----GKLFAY-VSVAFVAVTAVGLCLSTMPDVR-AEEERGECSTKCRNLF---------------------------------------------------------------------------------------------------------------------------------------------------------------------------------------------------------------------------------------------------VLETVCVAWFSFEFLLRSLQA-----------------ESKCAFLRTPL--------------------AIIDILAILPFYVSLLAGLAAGPTG----------------------------------------------------------------------------------SKMLERAG----------------------LVLRLLRALRVLYVMRLA-RHSLGL--------------------RSLGLTVRR--------CAREFGLLLLFL----CVA--MALFAPLVHLA--EREL-----------------------------------------------------------------------GAHRD------------------------------------------------------------------------FSSVPASYWWAVISMTTVGYGDMVPRSLPGQVVA-LSSILSGILLMAFPVTSIFHTFSRSYSE--------------------------------
82997534_172_443     --------------RGRQRLRDVVENP-----------HSGLA----GRLFAY-VSVAFVAVTAVGLCLSTMPDVR-AEEERGECSTKCRNLF---------------------------------------------------------------------------------------------------------------------------------------------------------------------------------------------------------------------------------------------------VLETVCVAWFSFEFLLRSLQA-----------------ESKCAFLRTPL--------------------AIIDILAILPFYVSLLAGLAAGPTG----------------------------------------------------------------------------------SKMLERAG----------------------LVLRLLRALRVLYVMRLA-RHSLGL--------------------RSLGLTVRR--------CAREFGLLLLFL----CVA--MALFAPLVHLA--EREL-----------------------------------------------------------------------GAHRD------------------------------------------------------------------------FSSVPASYWWAVISMTTVGYGDMVPRSLPGQVVA-LSSILSGILLMAFPVTSIFHTFSRSYSE--------------------------------
6912444_152_424      --------------RGRRRLRDVVDNP-----------HSGLA----GKLFAC-VSVSFVAVTAVGLCLSTMPDIR-AEEERGECSPKCRSLF---------------------------------------------------------------------------------------------------------------------------------------------------------------------------------------------------------------------------------------------------VLETVCVAWFSFEFLLRSLQA-----------------ESKCAFLRAPL--------------------NIIDILALLPFYVSLLLGLAAGPGG----------------------------------------------------------------------------------TKLLERAG----------------------LVLRLLRALRVLYVMRLA-RHSLGL--------------------RSLGLTMRR--------CAREFGLLLLFL----CVA--MALFAPLVHLA--EREL-----------------------------------------------------------------------GARRD------------------------------------------------------------------------FSSVPASYWWAVISMTTVGYGDMVPRSLPGQVVA-LSSILSGILLMAFPVTSIFHTFSRSYSE--------------------------------
2979611_192_472      --------------LCMRNLRDMVENP-----------HSGIP----GKIFAC-ISISFVAITAVSLCISTMPDVR-EEEDRGECSQKCYDIF---------------------------------------------------------------------------------------------------------------------------------------------------------------------------------------------------------------------------------------------------VLETVCVAWFSFEFLLRSIQA-----------------ENKCAFLKTPL--------------------NIIDILAILPFYISLIVDMASTKNSSKPG--------------------------------------------------------------------------GGAGNKYLERVG----------------------LVLRFLRALRILYVMRLA-RHSLGL--------------------QTLGLTVRR--------CTREFGLLLLFL----CVA--MALFSPLVYLA--ESEL-----------------------------------------------------------------------GAKQE------------------------------------------------------------------------FTSIPTSYWWAVISMTTVGYGDMVPRSIPGQVVA-LSSILSGILLMAFPVTSIFHTFSRSYSE--------------------------------
20887067_1_270       ----------------MNQLREMVEDP-----------QSGLP----GKVFAC-LSVLFVATTAVSLCVSTMPDFR-AEEGKGECTRKCYYIF---------------------------------------------------------------------------------------------------------------------------------------------------------------------------------------------------------------------------------------------------VVESICVAWFSLEFCLRFVQA-----------------PNKCQFFRGPL--------------------NVIDILAISPYYVSLAVSDESPEAGE-----------------------------------------------------------------------------RPSSSSYLEKVG----------------------LVLRVLRALRILYVMRLA-RHSLGL--------------------QTLGLTVRR--------CAREFGLLMLFL----AVA--VTLFSPLVYVA--ENES-----------------------------------------------------------------------GRVLE------------------------------------------------------------------------FTSIPASYWWAIISMTTVGYGDMVPRSVPGQMVA-LSSILSGILIMAFPATSIFHTFSHSYLE--------------------------------
22067069_196_473     --------------LCMNRLREMVENP-----------QSGLP----GKVFAC-LSILFVATTAVSLCVSTMPDLR-AEEDQGECSRKCYYIF---------------------------------------------------------------------------------------------------------------------------------------------------------------------------------------------------------------------------------------------------IVETICVAWFSLEFCLRFVQA-----------------QDKCQFFQGPL--------------------NIIDILAISPYYVSLAVSEEPPEDGE-----------------------------------------------------------------------------RPSGSSYLEKVG----------------------LVLRVLRALRILYVMRLA-RHSLGL--------------------QTLGLTVRR--------CTREFGLLLLFL----AVA--ITLFSPLVYVA--EKES-----------------------------------------------------------------------GRVLE------------------------------------------------------------------------FTSIPASYWWAIISMTTVGYGDMVPRSVPGQMVA-LSSILSGILIMAFPATSIFHTFSHSYLE--------------------------------
5679601_202_479      --------------RCMRRLRDMVERP-----------HSGLP----GKVFAC-LSVLFVTVTAVNLSVSTLPSLR-EEEEQGHCSQMCHNVF---------------------------------------------------------------------------------------------------------------------------------------------------------------------------------------------------------------------------------------------------IVESVCVGWFSLEFLLRLIQA-----------------PSKFAFLRSPL--------------------TLIDLVAILPYYITLLVDGAAAGRRK-----------------------------------------------------------------------------PGAGNSYLDKVG----------------------LVLRVLRALRILYVMRLA-RHSLGL--------------------QTLGLTARR--------CTREFGLLLLFL----CVA--IALFAPLLYVI--ENEM-----------------------------------------------------------------------ADSPE------------------------------------------------------------------------FTSIPACYWWAVITMTTVGYGDMVPRSTPGQVVA-LSSILSGILLMAFPVTSIFHTFSRSYLE--------------------------------
4504829_202_479      --------------RCMRRLRDMVERP-----------HSGLP----GKVFAC-LSVLFVTVTAVNLSVSTLPSLR-EEEEQGHCSQMCHNVF---------------------------------------------------------------------------------------------------------------------------------------------------------------------------------------------------------------------------------------------------IVESVCVGWFSLEFLLRLIQA-----------------PSKFAFLRSPL--------------------TLIDLVAILPYYITLLVDGAAAGRRK-----------------------------------------------------------------------------PGAGNSYLDKVG----------------------LVLRVLRALRILYVMRLA-RHSLGL--------------------QTLGLTARR--------CTREFGLLLLFL----CVA--IALFAPLLYVI--ENEM-----------------------------------------------------------------------ADSPE------------------------------------------------------------------------FTSIPACYWWAVITMTTVDYGDMVPRSTPGQVVA-LSSILSGILLMAFPVTSIFHTFSPSYLE--------------------------------
688438_191_481       --------------KLQWRVWLVLEYP-----------RTSRL----AMIYGV-TSFLFVVTSIAGFCLETLPELRPKFLN--------------------------------------------------------------------------------------------------------------VTTECEG----------------------------------------------------------------------------------------GHSRELG---------------------------------RVMTSHEALNTLDIICTVFFTLELILRFIFA-----------------PDKRKFIFSLM--------------------NVIDLLALVPLYVQVIFTKSDNMQ-----------------------------------------------------------------------------------ACYLNERFV--------------------IEIMFILRIIRMFRIFHLV-KHYQAL--------------------KVLVYAIKA--------SIQELLMLFIFL----LIG--MLIFSTMIYYA--ERKN-----------------------------------------------------------------------TTEGG----------------------------------------------------------------------DMFSTIPMGFWWSIITMTTVGYGDVYPTTPVGYIVG-TACSVSGVLLVALTIPVISNNFTLFYRH--------------------------------
6166005_105_367      --------------HVQGRVYNFLERP------------TGW-----KCFVYHFAVFLIVLVCLIFSVLSTIEQYAALATG------------------------------------------------------------------------------------------------------------------------------------------------------------------------------------------------------------------------------------------------------------TLFWMEIVLVVFFGTEYVVRLWSA--------GCRSKYVGLWGRLRFARKPI--------------------SIIDLIVVVASMVVLCVGSKGQVFAT---------------------------------------------------------------------------------------------------------------SAIRGIRFLQILRMLHVD-RQGGTW--------------------RLLGSVVFI--------HRQELITTLYIG------FLGLIFSSYFVYLA--EKDAVNESG--------------------------------------------------------------------------------------------------------------------------------------------RVEFGSYADALWWGVVTVTTIGYGDKVPQTWVGKTIA-SCFSVFAISFFALPAGILGSGFALKVQQ--------------------------------
5042385_105_367      --------------HVQGRVYNFLERP------------TGW-----KCFVYHFAVFLIVLVCLIFSVLSTIEQYAALATG------------------------------------------------------------------------------------------------------------------------------------------------------------------------------------------------------------------------------------------------------------TLFWMEIVLVVFFXTEYVVRLWSA--------GCRSKYVGLWGRLRFARKPI--------------------SIIDLIVVVASMVVLCVGSKGQVFAT---------------------------------------------------------------------------------------------------------------SAIRGIRFLQILRMLHVD-RQGGTW--------------------RLLGSVVFI--------HRQELITTLYIG------FLGLIFSSYFVYLA--EKDAVNESG--------------------------------------------------------------------------------------------------------------------------------------------RVEFGSYADALWWGVVTVTTIGYGDKVPQTWVGKTIA-SCFSVFAISFFALPAGILGSGFALKVQQ--------------------------------
2961249_1_240        ------------------------------------------------------MDFLIVLVCLIFSVLSTIEQYAALATG------------------------------------------------------------------------------------------------------------------------------------------------------------------------------------------------------------------------------------------------------------TLFWMEIVLVVFFGTEYVVRLWSA--------GCRSKYVGLWGRLRFARKPI--------------------SIIDLIVVVASMVVLCVGSKGQVFAT---------------------------------------------------------------------------------------------------------------SAIRGIRFLQILRMLHVD-RQGGTW--------------------RLLGSVVFI--------HRQELITTLYIG------FLGLIFSSYFVYLA--EKDAVNESG--------------------------------------------------------------------------------------------------------------------------------------------RVEFGSYADALWWGVVTVTTIGYGDKVPQTWVGKTIA-SCFSVFAISFFALPAGILGSGFALKVQQ--------------------------------
5042386_1_240        ------------------------------------------------------MDFLIVLVCLIFSVLSTIEQYAALATG------------------------------------------------------------------------------------------------------------------------------------------------------------------------------------------------------------------------------------------------------------TLFWMEIVLVVFFXTEYVVRLWSA--------GCRSKYVGLWGRLRFARKPI--------------------SIIDLIVVVASMVVLCVGSKGQVFAT---------------------------------------------------------------------------------------------------------------SAIRGIRFLQILRMLHVD-RQGGTW--------------------RLLGSVVFI--------HRQELITTLYIG------FLGLIFSSYFVYLA--EKDAVNESG--------------------------------------------------------------------------------------------------------------------------------------------RVEFGSYADALWWGVVTVTTIGYGDKVPQTWVGKTIA-SCFSVFAISFFALPAGILGSGFALKVQQ--------------------------------
20344303_40_302      --------------HIQGRVYNFLERP------------TGW-----KCFVYHFTVFLIVLVCLIFSVLSTIEQYAALATG------------------------------------------------------------------------------------------------------------------------------------------------------------------------------------------------------------------------------------------------------------TLFWMEIVLVVFFGTEYVVRLWSA--------GCRSKYVGIWGRLRFARKPI--------------------SIIDLIVVVASMVVLCVGSKGQVFAT---------------------------------------------------------------------------------------------------------------SAIRGIRFLQILRMLHVD-RQGGTW--------------------RLLGSVVFI--------HRQELITTLYIG------FLGLIFSSYFVYLA--EKDAVNESG--------------------------------------------------------------------------------------------------------------------------------------------RIEFGSYADALWWGVVTVTTIGYGDKVPQTWVGKTIA-SCFSVFAISFFALPAGILGSGFALKVQQ--------------------------------
14285397_71_333      --------------NIQGRVYNFLERP------------TGW-----KCFVYHFTVFLIVLICLIFSVLSTIQQYNNLATE------------------------------------------------------------------------------------------------------------------------------------------------------------------------------------------------------------------------------------------------------------TLFWMEIVLVVFFGAEYVVRLWSA--------GCRSKYVGVWGRLRFARKPI--------------------SVIDLIVVVASVIVLCVGSNGQVFAT---------------------------------------------------------------------------------------------------------------SAIRGIRFLQILRMLHVD-RQGGTW--------------------RLLGSVVFI--------HRQELITTLYIG------FLGLIFSSYFVYLA--EKDAIDSSG--------------------------------------------------------------------------------------------------------------------------------------------EYQFGSYADALWWGVVTVTTIGYGDKVPQTWIGKTIA-SCFSVFAISFFALPAGILGSGFALKVQQ--------------------------------
14285391_98_360      --------------NIQGRTYNFLERP------------TGW-----KCFIYHFTVFLIVLVCLIFSVMSTIEQYHYFANR------------------------------------------------------------------------------------------------------------------------------------------------------------------------------------------------------------------------------------------------------------ALVWMEIVLVVFFGTEYIVRLWSA--------GCRSKYVGFWGRLRFARKPI--------------------SIIDLIVVVASVIVLCVGSNGQVFAT---------------------------------------------------------------------------------------------------------------SAIRGIRFLQILRMLHVD-RQGGTW--------------------RLLGSVVFI--------HRQELITTLYIG------FLGLIFSSYFVYLA--EKDAVDDSG--------------------------------------------------------------------------------------------------------------------------------------------SQQFGSYADALWWGVVTVTTIGYGDKVPQTWIGRTIA-SCFSVFAISFFALPAGILGSGFALKVQQ--------------------------------
116131747_3_265      --------------------------------------------------------FLLVLVCLIFSVLSTIDEYSKFANE------------------------------------------------------------------------------------------------------------------------------------------------------------------------------------------------------------------------------------------------------------TLFWMEICLVVFFGVEYLVRLWSA--------GCRSKYMGFCGRLRFIRKPI--------------------CIIDLIVVVASMVVLSVGSNGQVFAT---------------------------------------------------------------------------------------------------------------SAIRGIRFLQILRMLHVD-RQGGTW--------------------RLLGSVVFI--------HRQELITTLYIG------FLGLIFSSYFVYLA--EKDVLGPDG--------------------------------------------------------------------------------------------------------------------------------------------KADFASYADALWWGVITVTTIGYGDTVPQTWMGKIVA-SCFSVFAISFFALPAGILGSGFALKVQQ--------------------------------
1928985_1_219        ------------------------------------------------------------LVCLIFSVLSTIEQYAALATG------------------------------------------------------------------------------------------------------------------------------------------------------------------------------------------------------------------------------------------------------------TLFWMEIVLVVFFGTKYVVRLWSA--------GCRTKYLGIWGRLRFARKPI--------------------SIIDLIVVVASMVVLCVGSKGQVFAT---------------------------------------------------------------------------------------------------------------SAIRGIRFLQILRMLHVD-RQGGTW--------------------RLLGSVFFI--------HRQELITTLYIG------FLGLIFSSYFVYLA--EKDAVNESG--------------------------------------------------------------------------------------------------------------------------------------------RIEFGSYADALWWGVVTVTTIGYGDKVPRHGLGRPSP-PVSLSSPYPSLHSQRAYLALGLH-------------------------------------
9651967_110_366      --------------RVQNYLYNVLERP------------RGW------AFIYHAFVFLLVFGCLILSVFSTIPEHTKLASS------------------------------------------------------------------------------------------------------------------------------------------------------------------------------------------------------------------------------------------------------------CLLILEFVMIVVFGLEFIIRIWSA--------GCCCRYRGWQGRLRFARKPF--------------------CVIDTIVLIASIAVVSAKTQGNIFAT---------------------------------------------------------------------------------------------------------------SALRSLRFLQILRMVRMD-RRGGTW--------------------KLLGSVVYA--------HSKELITAWYIG------FLVLIFSSFLVYLV--EKDA-------------------------------------------------------------------------------------------------------------------------------------------------NKEFSTYADALWWGTITLTTIGYGDKTPLTWLGRLLS-AGFALLGISFFALPAGILGSGFALKVQE--------------------------------
68534949_24_280      --------------RVQNYLYNVLERP------------RGW------AFVYHAFVFLLVFGCLILSVFSTIPEHTKLASS------------------------------------------------------------------------------------------------------------------------------------------------------------------------------------------------------------------------------------------------------------CLLILEFVMIVVFGLEFIIRIWSA--------GCCCRYRGWQGRLRFARKPF--------------------CVIDTIVLIASIAVVSAKTQGNIFAT---------------------------------------------------------------------------------------------------------------SALRSLRFLQILRMVRMD-RRGGTW--------------------KLLGSVVYA--------HSKELITAWYIG------FLVLIFSSFLVYLV--EKDA-------------------------------------------------------------------------------------------------------------------------------------------------NKEFSTYADALWWGTITLTTIGYGDKTPLTWLGRLLS-AGFALLGISFFALPAGILGSGFALKVQE--------------------------------
8132997_39_295       --------------RVQNYLYNVLERP------------RGW------AFIHHAFVFLLVFGCLILSVFSTIPEHTKLASS------------------------------------------------------------------------------------------------------------------------------------------------------------------------------------------------------------------------------------------------------------CLLILEFVMIVVFGLEFIIRIWSA--------GCCCRYRGWQGRLRFARKPF--------------------CVIDTIVLIASIAVVSAKTQGNIFAT---------------------------------------------------------------------------------------------------------------SALRSLRFLQILRMVRMD-RRGGTW--------------------KLLGSVVYA--------HSKELITAWYIG------FLVLIFSSFLVYLV--EKDA-------------------------------------------------------------------------------------------------------------------------------------------------NKEFSTYADALWWGTITLTTIGYGDKTPLTWLGRLLS-AGFALLGISFFALPAGILGSGFALKVQE--------------------------------
122065304_56_312     --------------RVQNYLYNVLERP------------RGW------AFVYHAFVFLLVFGCLILSVFSTIPEHTKLASS------------------------------------------------------------------------------------------------------------------------------------------------------------------------------------------------------------------------------------------------------------CLLILEFVMIVVFGLEFIIRIWSA--------GCCCRYRGWQGRLRFARKPF--------------------CVIDTIVLIASIAVVSAKTQGNIFAT---------------------------------------------------------------------------------------------------------------SALRSLRFLQILRMVRMD-RRGGTW--------------------KLLGSVVYA--------HSKELITAWYIG------FXVLIFSSFLVYLV--EKDA-------------------------------------------------------------------------------------------------------------------------------------------------NKEFSTYADALWWGTITLTTIGYGDKTPLTWLGRLLS-AGFALLGISFFALXAGIXGSGFALKVQE--------------------------------
26638653_82_338      --------------RLQNWVYNVLERP------------RGW------AFVYHVFIFLLVFSCLVLSVLSTIQEHQELANE------------------------------------------------------------------------------------------------------------------------------------------------------------------------------------------------------------------------------------------------------------CLLILEFVMIVVFGLEYIVRVWSA--------GCCCRYRGWQGRFRFARKPF--------------------CVIDFIVFVASVAVIAAGTQGNIFAT---------------------------------------------------------------------------------------------------------------SALRSMRFLQILRMVRMD-RRGGTW--------------------KLLGSVVYA--------HSKELITAWYIG------FLVLIFASFLVYLA--EKDA-------------------------------------------------------------------------------------------------------------------------------------------------NSDFSSYADSLWWGTITLTTIGYGDKTPHTWLGRVLA-AGFALLGISFFALPAGILGSGFALKVQE--------------------------------
2801452_76_332       --------------KLQNFLYNVLERP------------RGW------AFIYHAYVFLLVFSCLVLSVFSTIKEYEKSSEG------------------------------------------------------------------------------------------------------------------------------------------------------------------------------------------------------------------------------------------------------------ALYILEIVTIVVFGVEYFVRIWAA--------GCCCRYRGWRGRLKFARKPF--------------------CVIDIMVLIASIAVLAAGSQGNVFAT---------------------------------------------------------------------------------------------------------------SALRSLRFLQILRMIRMD-RRGGTW--------------------KLLGSVVYA--------HSKELVTAWYIG------FLCLILASFLVYLA--EKGE-------------------------------------------------------------------------------------------------------------------------------------------------NDHFDTYADALWWGLITLTTIGYGDKYPQTWNGRLLA-ATFTLIGVSFFALPAGILGSGFALKVQE--------------------------------
4176404_76_332       --------------KLQNFLYNVLERP------------RGW------AFIYHAYVFLLVFSCLVLSVFSTIKEYEKSSEG------------------------------------------------------------------------------------------------------------------------------------------------------------------------------------------------------------------------------------------------------------PLYILEIVTIVVFGVEYFVRIWAA--------GCCCRYRGWRGRLKFARKPF--------------------CVIDIMVLIASIAVLAAGSQGNVFAT---------------------------------------------------------------------------------------------------------------SALRSLRFLQILRMIRMD-RRGGTW--------------------KLLGSVVYA--------HSKELVTAWYIG------FLCLILASFLVYLA--EKGE-------------------------------------------------------------------------------------------------------------------------------------------------NDHFDTYADALWWGLITLTTIGYGDKYPQTWNGRLLA-ATFTLIGVSFFALPAGILGSGFALKVQE--------------------------------
6754438_76_322       --------------KLQNFLYNVLERP------------RGW------AFIYHAYVFLLVFSCLVLSVFSTIKEYEKSSEG------------------------------------------------------------------------------------------------------------------------------------------------------------------------------------------------------------------------------------------------------------ALYILEIVTIVVFGVEYFVRIWAA--------GCCCRYRGWRGRLKFARKPF--------------------CVIDIMVLIASIAVLAAGSQGNVFAT---------------------------------------------------------------------------------------------------------------SALRSLRFLQILRMIRMD-RRGGTW--------------------KLLGSVVYA--------HSKELVTAWYIG------FLCLILASFLVYLA--EKGE-------------------------------------------------------------------------------------------------------------------------------------------------NDHFDTYADALWWGLITLTTIGYGDKYPQTWNGRLLA-ATFTLIGVSFFALPA----------VSP--------------------------------
4758630_106_371      --------------RIQTLIYDALERP------------RGW------ALLYHALVFLIVLGCLILAVLTTFKEYETVSGD------------------------------------------------------------------------------------------------------------------------------------------------------------------------------------------------------------------------------------------------------------WLLLLETFAIFIFGAEFALRIWAA--------GCCCRYKGWRGRLKFARKPL--------------------CMLDIFVLIASVPVVAVGNQGNVLAT----------------------------------------------------------------------------------------------------------------SLRSLRFLQILRMLRMD-RRGGTW--------------------KLLGSAICA--------HSKELITAWYIG------FLTLILSSFLVYLV--EKDVPEVDAQGE---------------------------------------------------------------------------------------------------------------------------------------EMKEEFETYADALWWGLITLATIGYGDKTPKTWEGRLIA-ATFSLIGVSFFALPAGILGSGLALKVQE--------------------------------
14285393_106_371     --------------RIQTLIYDALERP------------RGW------ALLYHALVFLIVLGCLILAVLTTFREYETVSGD------------------------------------------------------------------------------------------------------------------------------------------------------------------------------------------------------------------------------------------------------------WLLLLETFAIFIFGAEFALRIWAA--------GCCCRYKGWRGRLKFARKPL--------------------CMLDIFVLIASVPVVAVGNQGNVLAT----------------------------------------------------------------------------------------------------------------SLRSLRFLQILRMLRMD-RRGGTW--------------------KLLGSAICA--------HSKELITAWYIG------FLTLILSSFLVYLV--EKDVPEVDAQGE---------------------------------------------------------------------------------------------------------------------------------------EMKEEFETYADALWWGLITLATIGYGDKTPKTWEGRLIA-ATFSLIGVSFFALPAGILGSGLALKVQE--------------------------------
23592726_107_372     --------------RIQTLIYDALERP------------RGW------ALLYHALVFLIVLGCLILAVLTTFKEYETVSGD------------------------------------------------------------------------------------------------------------------------------------------------------------------------------------------------------------------------------------------------------------WLLLLETFAIFIFGAEFALRIWAA--------GCCCRYKGWRGRLKFARKPL--------------------CMLDIFVLIASVPVVAVGNQGNVLAT----------------------------------------------------------------------------------------------------------------SLRSLRFLQILRMLRMD-RRGGTW--------------------KLLGSAICA--------HSKELITAWYIG------FLTLILSSFLVYLV--EKDVPEMDAQGE---------------------------------------------------------------------------------------------------------------------------------------EMKEEFETYADALWWGLITLATIGYGDKTPKTWEGRLIA-ATFSLIGVSFFALPAGILGSGLALKVQE--------------------------------
55239106_30_288      --------------RLQSRVYNFLERP------------RGF-----KAIFYHVCVFLMVFTCLALSVFSTIQEYEDQAIA------------------------------------------------------------------------------------------------------------------------------------------------------------------------------------------------------------------------------------------------------------ILFVMEIIVVIWFSIEFFLRLWSS--------GCRSRYQGAVGRLKFLKRPF--------------------CIIDIVTIAASIVVLVMGTSGQVFAT---------------------------------------------------------------------------------------------------------------SALRGLRFFQILRMVRMD-RRGGTW--------------------KLLGSVVYA--------HRQELITTLYIG------FLGLIFASFLVYLM--EKDVRG------------------------------------------------------------------------------------------------------------------------------------------------TKFSNFAQALWWGVITLCTVGYGDMVPETWQGKIIA-SFCALLGISFFALPAGILGSGFALKVQQ--------------------------------
23240346_56_312      --------------RLQSRLYNFLERP------------RGL-----HAIFYHVMVFLMVFTCLALSVFSTIKEYEEDAVY------------------------------------------------------------------------------------------------------------------------------------------------------------------------------------------------------------------------------------------------------------ILFRMEILVVIWFTMEFGARLWSS--------GCRSRYQGCLGRLKFVKRPF--------------------CIIDIVTILASIVVLGMGTSGQVFAT---------------------------------------------------------------------------------------------------------------SALRGLRFFQILRMVRMD-RRGGTW--------------------KLLGSVVYA--------HRQELITTMYIG------FLGLIFASFLVYMW--EKDVN-------------------------------------------------------------------------------------------------------------------------------------------------DKFSNFAQALWWG-ITLCTVGYGDMVPITWQGKLIA-SCCALLGISFFALPAGILGSGFALKVQQ--------------------------------
7511689_107_364      --------------RMQNKMHNFLERP------------RGW-----KAATYHLAVLFMVLMCLALSVFSTMPDFEVNATI------------------------------------------------------------------------------------------------------------------------------------------------------------------------------------------------------------------------------------------------------------VLYYLEIVFVIWLATEYICRVWSA--------GCRSRYRGISGRIRFATSAY--------------------CVIDIIVILASITVLCIGATGQVFAA---------------------------------------------------------------------------------------------------------------SAIRGLRFFQILRMLRID-RRAGTW--------------------KLLGSVVWA--------HRQELLTTVYIG------FLGLIFSSFLVYLC--EKNTN-------------------------------------------------------------------------------------------------------------------------------------------------DKYQTFADALWWGVITLSTVGYGDKTPETWPGKIIA-AFCALLGISFFALPAGILGSGFALKVQQ--------------------------------
45945867_95_684      --------------GADVLPEYKLQAP----------RIHRWTILH-YSPFKAVWDWLILLLVIYTAVFTPYSAAFLLKETEE--------------------------------------------------------------------------------------------------------------------------------------------------------------------------------------------------------------GPPATE--CGYACQ------------------------------PLAVVDLIVDIMFIVDILINFRTTYVNANE---EVVSH-PGRIAVHYFKGW---------------------FLIDMVAAIPFDLLIFGSGSE------------------------------------------------------------------------------------------ELI--------------------------GLLKTARLLRLVRVA-RKLDRY--------------------SXYGAAVLF--------LLMCTFALIAH---------WLACIWYAIGNM--EQPHMD---------------------------------------------------------------SRIGWLHNLGDQIGKPYNSSG--------------------------------------------------------LGGPSIKDKYVTALYFTFSSLTSVGFGNVSPNTNSEKIFS-ICVMLIGSLMYASIFGNVSAIIQRLYSGTARYHTQMLRVREFIRFHQ-------------
3452413_185_814      --------------GADVLPEYKLQAP----------RIHRWTILH-YSPFKAVWDWLILLLVIYTAVFTPYSAAFLLKETEE--------------------------------------------------------------------------------------------------------------------------------------------------------------------------------------------------------------GPPATE--CGYACQ------------------------------PLAVVDLIVDIMFIVDILINFRTTYVNANE---EVVSH-PGRIAVHYFKGW---------------------FLIDMVAAIPFDLLIFGSGSE------------------------------------------------------------------------------------------ELI--------------------------GLLKTARLLRLVRVA-RKLDRY--------------------SEYGAAVLF--------LLMCTFALIAH---------WLACIWYAIGNM--EQPHMD---------------------------------------------------------------SRIGWLHNLGDQIGKPYNSSG--------------------------------------------------------LGGPSIKDKYVTALYFTFSSLTSVGFGNVSPNTNSEKIFS-ICVMLIGSLMYASIFGNVSAIIQRLYSGTARYHTQMLRVREFIRFHQ-------------
4557729_211_840      --------------GADVLPEYKLQAP----------RIHRWTILH-YSPFKAVWDWLILLLVIYTAVFTPYSAAFLLKETEE--------------------------------------------------------------------------------------------------------------------------------------------------------------------------------------------------------------GPPAPD--CGYACQ------------------------------PLAVVDFIVDIMFIVDILINFRTTYVNANE---EVVSH-PGRIAVHYFKGW---------------------FLIDMVAAIPFDLLIFGSGSE------------------------------------------------------------------------------------------ELI--------------------------GLLKTARLLRLVRVA-RKLDRY--------------------SEYGAAVLF--------LLMCTFALIAH---------WLACIWYAIGNM--EQPHMD---------------------------------------------------------------SRIGWLHNLGDQIGKPYNSSG--------------------------------------------------------LGGPSIKDKYVTALYFTFSSLTSVGFGNVSPNTNSEKIFS-ICVMLIGSLMYASIFGNVSAIIQRLYSGTARYHTQMLRVREFIRFHQ-------------
6687230_211_839      --------------GADVLPEYKLQAP----------RIHRWTILH-YSPFKAVWDWLILLLVIYTAVFTPYSAAFLLKETEE--------------------------------------------------------------------------------------------------------------------------------------------------------------------------------------------------------------GPPAPE--CGYACQ------------------------------PLAVVDLIVDIMFIVDILINFRTTYVNANE---EVVSH-PGRIAVHYFKGW---------------------FLIDMVAAIPFDLLIFGSGSE------------------------------------------------------------------------------------------ELI--------------------------GLLKTARLLRLVRVA-RKLDRY--------------------SEYGAAVLL--------LLMCTFALIAH---------WLACIWYAIGNM--EQPHMD---------------------------------------------------------------SRIGWLHNLGDQMGKPYNSSG--------------------------------------------------------LGGPSIKDKYVTGLYFTFSSLTSVGFGNVSPNTNSEKIFS-ICVMLIGSLMYASIFGNVSAIIQRLYSGTARYHTQMLRVREFIRFHQ-------------
7305203_211_842      --------------GADVLPEYKLQAP----------RIHRWTILH-YSPFKAVWDWLILLLVIYTAVFTPYSAAFLLKETED--------------------------------------------------------------------------------------------------------------------------------------------------------------------------------------------------------------GSQAPD--CGYACQ------------------------------PLAVVDLIVDIMFIVDILINFRTTYVNANE---EVVSH-PGRIAVHYFKGW---------------------FLIDMVAAIPFDLLIFGSGSE------------------------------------------------------------------------------------------ELI--------------------------GLLKTARLLRLVRAA-RKLDRY--------------------SEYGAAVLF--------LLMCTFALIAH---------WLACIWYAIGNM--EQPHMD---------------------------------------------------------------SHIGWLHNLGDQIGKPYNSSG--------------------------------------------------------LGGPSIKDKYVTALYFTFSSLTSVGFGNVSPNTNSEKIFS-ICVMLIGSLMYASIFGNVSAIIQRLYSGTARYHTQMLRVREFIRFHQ-------------
2351698_194_825      --------------GADVLPEYKLQAP----------RIHRWTILH-YSPFKAVWDWLILLLVIYTAVFTPYSAAFLLKETED--------------------------------------------------------------------------------------------------------------------------------------------------------------------------------------------------------------GSQAPD--CGYACQ------------------------------PLAVVDLIVDIMFIVDILINFRTTYVNANE---EVVSH-PGRIAVHYFKGW---------------------FLIDMVAAIPFDLLIFGSGSE------------------------------------------------------------------------------------------ELI--------------------------GLLKTARLLRLVRVA-RKLDRY--------------------SEYGAAVLF--------LLMCTFALIAH---------WLACIWYAIGNM--EQPHMD---------------------------------------------------------------SHIGWLHNLGDQIGKPYNSSG--------------------------------------------------------LGGPSIKDKYVTALYFTFSSLTSVGFGNVSPNTNSEKIFS-ICVMLIGSLMYASIFGNVSAIIQRLYSGTARYHTQMLRVREFIRFHQ-------------
21717341_211_842     --------------GADVLPEYKLQAP----------RIHRWTILH-YSPFKAVWDWLILLLVIYTAVFTPYSAAFLLKETED--------------------------------------------------------------------------------------------------------------------------------------------------------------------------------------------------------------GSQAPD--CGYACQ------------------------------PLTVVDLIVDIMFIVDILINFRTTYVNANE---EVVSH-PGRIAVHYFKGW---------------------FLIDMVAAIPFDLLIFGSGSE------------------------------------------------------------------------------------------ELI--------------------------GLLKTARLLRLVRVA-RKLDRY--------------------SEYGAAVLF--------LLMCTFALIAH---------WLACIWYAIGNM--EQPHMD---------------------------------------------------------------SHIGWLHNLGDQIGKPYNSSG--------------------------------------------------------LGGPSIKDKYVTALYFTFSSLTSVGFGNVSPNTNSEKIFS-ICVMLIGSLMYASIFGNVSAIIQRLYSGTARYHTQMLRVREFIRFHQ-------------
2582017_152_783      --------------GADVLPEYKLQAP----------RIHRWTILH-YSPFKAVWDWLILLLVIYTAVFTPYSAAFLLKETED--------------------------------------------------------------------------------------------------------------------------------------------------------------------------------------------------------------GSQAPD--CGYACQ------------------------------PLAVVDLLVDIMFIVDILINFRTTYVNANE---EVVSH-PGRIAVHYFKGW---------------------FLIDMVAAIPFDLLIFGSGSE------------------------------------------------------------------------------------------ELI--------------------------GLLKTARLLRLVRVA-RKLDRY--------------------SEYGAAVLF--------LLMCTFALIAH---------WLACIWYAIGNM--EQPHMD---------------------------------------------------------------SHIGWLHNLGDQIGKPYNSSG--------------------------------------------------------LGGPSIKDKYVTALYFTFSSLTSVGFGNVSPNTNSEKIFS-ICVMLIGSLMYASIFGNVSAIIQRLYSGTARYHTQMLRVREFIRFHQ-------------
6706732_174_692      --------------GADVLPEYKLQAP----------RIHRWTILH-YSPFKAVWDWLILLLVIYTAVFTPYSAAFLLSDQD---------------------------------------------------------------------------------------------------------------------------------------------------------------------------------------------------------------ESRRGA--CSYTCS------------------------------PLTVVDLIVDIMFVVDIVINFRTTYVNTND---EVVSH-PRRIAVHYFKGW---------------------FLIDMVAAIPFDLLIFRTGSD------------------------------------------------------------------------------------------ETT-----------------------TLIGLLKTARLLRLVRVA-RKLDRY--------------------SEYGAAVLF--------LLMCTFALIAH---------WLACIWYAIGNV--ERPYLE---------------------------------------------------------------HKIGWLDSLGVQLGKRYNGSDP-------------------------------------------------------ASGPSVQDKYVTALYFTFSSLTSVGFGNVSPNTNSEKVFS-ICVMLIGSLMYASIFGNVSAIIQRLYSGTARYHTQMLRVKEFIRFHQ-------------
13540549_174_692     --------------GADVLPEYKLQAP----------RIHRWTILH-YSPFKAVWDWLILLLVIYTAVFTPYSAAFLLSDQD---------------------------------------------------------------------------------------------------------------------------------------------------------------------------------------------------------------ESRRGA--CSYTCS------------------------------PLTVVDLIVDIMFVVDIVINFRTTYVNTND---EVVSH-PRRIAVHYFKGW---------------------FLIDMVAAIPFDLLIFRTGSD------------------------------------------------------------------------------------------ETT-----------------------TLIGLLKTARLLRLVRVA-RKLDRY--------------------SEYGAAVLF--------LLMCTFALIAH---------WLACIC-----------------------------------------------------------------------------------------------------------------------------------------------------------------------------SLTSVGFGNVSPNTNSEKVFS-ICVMLIGSLMYASIFGNVSAIIQRLYSGTARYHTQMLRVKEFIRFHQ-------------
16758818_16_526      --------------GADVLPEYKLQAP----------RIHRGTILH-YSPFKAVWDWLILLLVIYTAVFTPYSAAFLLSDQD---------------------------------------------------------------------------------------------------------------------------------------------------------------------------------------------------------------ESQRGT--CGYTCS------------------------------PLTVVDLIVDIMFVVDIVINFRTTYVNTND---EVVSH-PRRIAVHYFKGW---------------------FLIDMVAAIPFDLLIFRTGSD------------------------------------------------------------------------------------------ETT-----------------------TLIGLLKTARLLRLVRVA-RKLDRY--------------------SEYGAAVLF--------LLMCTFALIAH---------WLACIWYAIGNV--ERPYLE---------------------------------------------------------------PKIGWLDSLGAQLGKQYNGSDP-------------------------------------------------------ASGPSVQDKYVTALYFTFSSLTSVGFGNVSPNTNSEKVFS-ICVMLIGSLMYASIFGNVSAIIQRLYSGTARYHTQMLRVKEFIRFHQ-------------
26006812_210_812     --------------GADVLPEYKLQAP----------RIHRWTILH-YSPFKAVWDWLILLLVIYTAVFTPYSAAFLLNEEQG--------------------------------------------------------------------------------------------------------------------------------------------------------------------------------------------------------------EEKHWN--CSYSCD------------------------------PLNIIDLIVDIMFIVDIVINFRTTYVNIND---EVVSH-PGKIAIHYFKGW---------------------FLIDMVAAIPFDLLIFRSGSD------------------------------------------------------------------------------------------ETT-----------------------TLIGLLKTARLLRLVRVA-RKLDRY--------------------SEYGAAVLF--------LLMCTFALIAH---------WLACIWYAIGNV--ERPYME---------------------------------------------------------------HKIGWLDNLGDQIGKRYNDSDL-------------------------------------------------------SSGPSIKDKYVTALYFTFSSLTSVGFGNVSPNTNSEKIFS-ICVMLIGSLMYASIFGNVSAIIQRLYSGTARYHTQMLRVKEFIRFHQ-------------
149730657_379_846    --------------GADVLPEYKLQTP----------RINKFTILH-YSPFKAVWDWLILLLVIYTAIFTPYSAAFLLNDRE---------------------------------------------------------------------------------------------------------------------------------------------------------------------------------------------------------------EQKRRE--CGYSCS------------------------------PLNVVDLIVDIMFIIDILINFRTTYVNQNE---EVVSD-PAKIAIHYFKGW---------------------FLIDMVAAIPFDLLIFGSGSD------------------------------------------------------------------------------------------ETT-----------------------TLIGLLKTARLLRLVRVA-RKLDRY--------------------SEYGAAVLM--------LLMCIFALIAH---------WLACIWYAIGNV--ERPYLT---------------------------------------------------------------DKIGWLDSLGQQIGKRYNDSDS-------------------------------------------------------SSGPSIKDKYVTALYFTFSSLTSVGFGNVSPNTNSEKIFS-ICVMLIGSLMYASIFGNVSAIIQRLYSGTARYHMQMLRVKEFIRFHQ-------------
18777774_213_843     --------------GADVLPEYKLQTP----------RINKFTILH-YSPFKAVWDWLILLLVIYTAIFTPYSAAFLLNDRE---------------------------------------------------------------------------------------------------------------------------------------------------------------------------------------------------------------EQKRRE--CGYSCS------------------------------PLNVVDLIVDIMFIIDILINFRTTYVNQNE---EVVSD-PAKIAIHYFKGW---------------------FLIDMVAAIPFDLLIFGSGSD------------------------------------------------------------------------------------------ETT-----------------------TLIGLLKTARLLRLVRVA-RKLDRY--------------------SEYGAAVLM--------LLMCIFALIAH---------WLACIWYAIGNV--ERPYLT---------------------------------------------------------------DKIGWLDSLGTQIGKRYNDSDS-------------------------------------------------------SSGPSIKDKYVTALYFTFSSLTSVGFGNVSPNTNSEKIFS-ICVMLIGSLMYASIFGNVSAIIQRLYSGTARYHMQMLRVKEFIRFHQ-------------
26006809_213_843     --------------GADVLPEYKLQTP----------RINKFTILH-YSPFKAVWDWLILLLVIYTAIFTPYSAAFLLNDRE---------------------------------------------------------------------------------------------------------------------------------------------------------------------------------------------------------------EQKRRE--CGYSCS------------------------------PLNVVDLIVDIMFIIDILINFRTTYVNQNE---EVVSD-PAKIAIHYFKGW---------------------FLIDMVAAIPFDLLVFGSGSD------------------------------------------------------------------------------------------ETT-----------------------TLIGLLKTARLLRLVRVA-RKLDRY--------------------SEYGAAVLM--------LLMCIFALIAH---------WLACIWYAIGNV--ERPYLT---------------------------------------------------------------DKIGWLDSLGTQIGKRYNDSDS-------------------------------------------------------SSGPSIKDKYVTALYFTFSSLTSVGFGNVSPNTNSEKIFS-ICVMLIGSLMYASIFGNVSAIIQRLYSGTARYHMQMLRVKEFIRFHQ-------------
11121258_213_843     --------------GADVLPEYKLQTP----------RINKFTILH-YSPFKAVWDWLILLLVIYTAIFTPYSAAFLLNDRE---------------------------------------------------------------------------------------------------------------------------------------------------------------------------------------------------------------EQKRRE--CGYSCS------------------------------PLNVVDLIVDIMFIIDILINFRTTYVNQNE---EVVSD-PAKIAIHYFKGW---------------------FLIDMVAAIPFDLLIFGSGSD------------------------------------------------------------------------------------------ETT-----------------------TLIGLLKTARLLRLVRVA-RKLDRY--------------------SEYGAAVLM--------LSMCIFALNAH---------WLACIWYAIGNV--ERPYLT---------------------------------------------------------------DKIGWLDSLGQQIGKRYNDSDS-------------------------------------------------------SSGPSIKDKYVTALYFTFSSLTSVGFGNVSPNTNSEKIFS-ICVMLIGSLMYASIFGNVSAIIQRLYSGTARYHMQMLRVKEFIRFHQ-------------
47086359             --------------GADVLPEYKLQAP----------RIHKWTILH-YSPFKAVWDWIILLLVLYTAVFTPYSAAFLLNEQE---------------------------------------------------------------------------------------------------------------------------------------------------------------------------------------------------------------DERRRT--CGYTCN------------------------------PLNVVDLVVDVMFIIDILINFRTTYVNHND---EVVSN-PARIAQHYFKGW---------------------FLIDIVAAIPFDLLIFRSGSD------------------------------------------------------------------------------------------EPQ---------------------TTTLIGLLKTARLLRLVRVA-RKLDRY--------------------SEYGAAVLF--------LLMCTFALIAH---------WLACIWYAIGNM--ERTSSARIG-----------------------------------------------------------GMKIGWLDNLADQIGKQYNDSNS-------------------------------------------------------FSGPSIKDKYVTALYFTFSSLTSVGFGNVSPNTNPEKIFS-ICVMLIGSLMYASIFGNVSAIIQRLYSGTARYHTQMLRVKEFIRFHQ-------------
21750348_174_639     --------------GADVLPEYKLQAP----------RIHKWTILH-YSPFKAVWDWIILLLVMYTAIFTPYVAAFLLSEPD---------------------------------------------------------------------------------------------------------------------------------------------------------------------------------------------------------------YNQRKN--RKYADD------------------------------PIVIIDLIVDVTFVVDILINFRTTFVNGQD---EVVSH-PGRIAVHYLSGW---------------------FLIDLVAAIPFDLLLVGSDTD------------------------------------------------------------------------------------------ETT-----------------------TLIGLLKTARLLRLVRVA-RKIDRY--------------------SEYGAAVLV--------LLMATFALIAH---------WLACIWYAIGNA--ERPLLK---------------------------------------------------------------AKIGWLDALAQDTQEYYFPNN-------------------------------------------------------TGGGPSVKSRYVTALYFTFTSLTSVGFGNVAPNTDAEKIFT-ICVMLVGSLMYASIFGNVSAIIQRLYSGTARYHTQMLRVREFIRFHQ-------------
17136454             --------------GGNILLDQKLQNN----------YYHKWTLLH-YSPFKAVWDWIILILVMYTAIFTPYVAAFLLGEQD----------------------------------------------------------------------------------------------------------------------------------------------------------------------------------------------------------------YQRRN--SKYINSD-----------------------------PIVIIDLIVDVTFIVDIIINFRTTFVNSQD---EVVSH-PGRIAVHYLSGW---------------------FLIDLVAAVPFDLLLVGSDTD------------------------------------------------------------------------------------------ETT-----------------------TLIGLLKTARLLRLVRVA-RKIDRY--------------------SEYGAAVLI--------LLMATFILIAH---------WLACIWYAIGNA--EKSIAS---------------------------------------------------------------KNIGWLNSLAYDIQEPYFDNR-------------------------------------------------------TGG-PSIKSRYITALYFTFTSLTSVGFGNVAPNTDAEKAFT-ICVMLVGSLMYASIFGNVSAIIQRLYSGTARYHTQMLRVREFIRFHQ-------------
17555416             --------------GADVLPEYKLQPT----------RIHHCTIVH-YSPFKAVWDWIILLLVIYTAVFTPYVAAFLLRELQD---------------------------------------------------------------------------------------------------------------------------------------------------------------------------------------------------------------TAKK---SRFTE-------------------------------PLEIVDLIVDIMFIVDIIINFRTTYVNENDEACQVVSD-PGKIATHYFKGW---------------------FIIDMVAAVPFDLLLVSTNSD------------------------------------------------------------------------------------------ETT-----------------------TLIGLLKTARLLRLVRVA-RKLDRY--------------------SEYGAAVLL--------LLMATFALIAH---------WLACIWYAIGSA--ELSHKE-----------------------------------------------------------------YTWLHQLSKQLAQPYTSTNGTI----------------------------------------------------PTGGPTLKSRYVTSLYFTLSTITSIGFGNVSATTDSEKIFT-IIMMILGSLMYASVFGNVSAIIQRLYSGTARYHTEMSRLREFIRFHQ-------------
149027427            --------------DKPAFPEYKVSDA----------KKSKFILLH-FSTFKAGWDWLILLATFYVAVTVPYNVCFIGNED----------------------------------------------------------------------------------------------------------------------------------------------------------------------------------------------------------------LSTTR---------------------------------------STTVSDIAVEILFIIDIILNFRTTYVSKSG---QVIFE-ARSICIHYVTTW---------------------FIIDLIAALPFDLLYAFNVTV------------------------------------------------------------------------------------------VSL-------------------------VHLLKTVRLLRLLRLL-QKLDRY--------------------SQHSTIVLT--------LLMSMFALLAH---------WMACIWYVIGKM--EREDNSLL------------------------------------------------------------KWEVGWLHELGKRLESPYYGNN-------------------------------------------------------TLGGPSIRSAYIAALYFTLSSLTSVGFGNVSANTDAEKIFS-ICTMLIGALMHALVFGNVTAIIQRMYSRWSLYHTRTKDLKDFIRVHH-------------
114585665            --------------DKPAFPEYKVSDA----------KKSKFILLH-FSTFKAGWDWLILLATFYVAVTVPYNVCFIGNDD----------------------------------------------------------------------------------------------------------------------------------------------------------------------------------------------------------------LSTTR---------------------------------------STTVSDIAVEILFIIDIILNFRTTYVSKSG---QVIFE-ARSICIHYVTTW---------------------FIIDLIAALPFDLLYAFNVTV------------------------------------------------------------------------------------------VSL-------------------------VHLLKTVRLLRLLRLL-QKLDRY--------------------SQHSTIVLT--------LLMSMFALLAH---------WMACIWYVIGKM--EREDNSLL------------------------------------------------------------KWEVGWLHELGKRLESPYYGNN-------------------------------------------------------TLGGPSIRSAYIAALYFTLSSLTSVGFGNVSANTDAEKIFS-ICTMLIGALMHALVFGNVTAIIQRMYSRWSLYHTRTKDLKDFIRVHH-------------
6912446              --------------PKPSVPEYKVASV----------GGSRCLLLH-YSVSKAIWDGLILLATFYVAVTVPYNVCFSGDDDT---------------------------------------------------------------------------------------------------------------------------------------------------------------------------------------------------------------PITSR---------------------------------------HTLVSDIAVEMLFILDIILNFRTTYVSQSG---QVISA-PRSIGLHYLATW---------------------FFIDLIAALPFDLLYIFNITV------------------------------------------------------------------------------------------TSL-------------------------VHLLKTVRLLRLLRLL-QKLERY--------------------SQCSAVVLT--------LLMSVFALLAH---------WMACIWYVIGRR--EMEANDPL------------------------------------------------------------LWDIGWLHELGKRLEVPYVNG--------------------------------------------------------SVGGPSRRSAYIAALYFTLSSLTSVGFGNVCANTDAEKIFS-ICTMLIGALMHAVVFGNVTAIIQRMYSRRSLYHSRMKDLKDFIRVHR-------------
148670587            --------------PKPSVPEYKVASV----------GGSRCLLLH-YSIPKAVWDGLILLATFYVAVTVPYNVCFAGDDDT---------------------------------------------------------------------------------------------------------------------------------------------------------------------------------------------------------------PITSR---------------------------------------HTLVSDIAVEMLFILDIILNFRTTYVSQSG---QVVSA-PRSIGLHYLATW---------------------FFVDLIAALPFDLLYVFNITV------------------------------------------------------------------------------------------TSL-------------------------VHLLKTVRLLRLLRLL-QKLERY--------------------SQCSAVVLT--------LLMSVFALLAH---------WMACVWYVIGRR--EMEANDPL------------------------------------------------------------LWDIGWLHELGKRLEEPYVNG--------------------------------------------------------SAGGPSRRSAYIAALYFTLSSLTSVGFGNVCANTDAEKIFS-ICTMLIGALMHAVVFGNVTAIIQRMYSRRSLYHSRMKDLKDFIRVHR-------------
149032086            --------------EKPNLPEYKVAAI----------RKSPFILLH-CGALRATWDGFILLATLYVAVTVPYSVCVSTAREP----------------------------------------------------------------------------------------------------------------------------------------------------------------------------------------------------------------SAARG--------------------------------------PPSVCDLAVEVLFILDIVLNFRTTFVSKSG---QVVFA-PKSICLHYVTTW---------------------FLLDVIAALPFDLLHAFKVNV-----------------------------------------------------------------------------------------YVG--------------------------AHLLKTVRLLRLLRLL-PRLDRY--------------------SQYSAVVLT--------LLMAVFALLAH---------WVACVWFYIGQQ--EIENSESE------------------------------------------------------------LPEIGWLQELARRLETPYYLVSRSPDGGNSSGQSENCSSSGGG-------------------------SEANGTGLELLGGPSLRSAYITSLYFALSSLTSVGFGNVSANTDTEKIFS-ICTMLIGALMHAVVFGNVTAIIQRMYARRFLYHSRTRDLRDYIRIHR-------------
119578478            --------------EKPNLPEYKVAAI----------RKSPFILLH-CGALRATWDGFILLATLYVAVTVPYSVCVSTAREP----------------------------------------------------------------------------------------------------------------------------------------------------------------------------------------------------------------SAARG--------------------------------------PPSVCDLAVEVLFILDIVLNFRTTFVSKSG---QVVFA-PKSICLHYVTTW---------------------FLLDVIAALPFDLLHAFKVNV-----------------------------------------------------------------------------------------YFG--------------------------AHLLKTVRLLRLLRLL-PRLDRY--------------------SQYSAVVLT--------LLMAVFALLAH---------WVACVWFYIGQR--EIESSESE------------------------------------------------------------LPEIGWLQELARRLETPYYLVGRRPAGGNSSGQSDNCSSS----------------------------SEANGTGLELLGGPSLRSAYITSLYFALSSLTSVGFGNVSANTDTEKIFS-ICTMLIGALMHAVVFGNVTAIIQRMYARRFLYHSRTRDLRDYIRIHR-------------
119113968            --------------SEAPLPEYKTQAL----------KKSRFILSH-YGMFKGCWDWMILVATFYVAIAVPYNAAFV---------------------------------------------------------------------------------------------------------------------------------------------------------------------------------------------------------------------KTDR---------------------------------------LTMVSDVVVEALFIVDILVNFRTTYVSRKG---EVVSD-SKSIALNYLRSW---------------------FVVDLLAALPFDHLYASNVIS-----------------------------------------------------------------------------------------GEE------------------------SHIHLVKLTRLLRLARLL-QKMDRY--------------------SQYTAMILT--------LLMLCFSLVAH---------WLACVWFVIAEK--ERLMNDA-------------------------------------------------------------DWDIGWIHTLAERLKIP-------------------------------------------------------------VSNVTHGEAYITALYFTFTSLTSVGFGNVSATTLSEKIFS-IIMMLIGALMHAVVFGNVTAIIQRMYSRRSLYQSKWRDLKDFIALHQ-------------
17136946             --------------TEAPFPEYKTQSI----------KKSRLILPH-YGVFKGIWDWVILVATFYVALMVPYNAAFA---------------------------------------------------------------------------------------------------------------------------------------------------------------------------------------------------------------------KADR---------------------------------------QTKVSDVIVEALFIVDILLNFRTTFVSRKG---EVVSN-SKQIAINYLRGW---------------------FALDLLAALPFDHLYASDLYD-----------------------------------------------------------------------------------------GED------------------------SHIHLVKLTRLLRLARLL-QKIDRY--------------------SQHTAMILT--------LLMFSFTLAAH---------WLACIWYVIAVK--EYEWFP--------------------------------------------------------------ESNIGWLQLLAERKNAS-------------------------------------------------------------VAILTTAETYSTALYFTFTSLTSVGFGNVSANTTAEKVFT-IIMMLIGALMHAVVFGNVTAIIQRMYSRRSLYESKWRDLKDFVALHN-------------
109018388            --------------GSDILPQYKQEAP----------KTPPHIILH-YCVFKTTWDWIILILTFYTAILVPYNVSFKT--------------------------------------------------------------------------------------------------------------------------------------------------------------------------------------------------------------------RQNN-----V---------------------------------AWLVVDSIVDVIFLVDIVLNFHTTFVGPAG---EVISD-PKLIRMNYLKTW---------------------FVIDLLSCLPYDVINAFENVD------------------------------------------------------------------------------------------EGI----------------------SSLFSSLKVVRLLRLGRVA-RKLDHY--------------------IEYGAAVLV--------LLVCVFGLAAH---------WMACIWYSIGDY--EIFDEDTKT-----------------------------------------------------------IRNNSWLYQLAMDIGTPYQFNGSGSGK--------------------------------------------------WEGGPSKNSVYISSLYFTMTSLTSVGFGNIAPSTDIEKIFA-VAIMMIGSLLYATIFGNVTTIFQQMYANTNRYHEMLNSVRDFLKLYQ-------------
80478033             --------------GSDILPQYKQEAP----------KTPPHIILH-YCVFKTTWDWIILILTFYTAILVPYNVSFKT--------------------------------------------------------------------------------------------------------------------------------------------------------------------------------------------------------------------RQNN-----V---------------------------------AWLVVDSIVDVIFLVDIVLNFHTTFVGPAG---EVISD-PKLIRMNYLKTW---------------------FVIDLLSCLPYDVINAFENVD------------------------------------------------------------------------------------------EGI----------------------SSLFSSLKVVRLLRLGRVA-RKLDHY--------------------IEYGAAVLV--------LLVCVFGLAAH---------WMACIWYSIGDY--EIFDEDTKT-----------------------------------------------------------IRNNSWLYQLALDIGTPYQFNGSGSGK--------------------------------------------------WEGGPSKNSVYISSLYFTMTSLTSVGFGNIAPSTDIEKIFA-VAIMMIGSLLYATIFGNVTTIFQQMYANTNRYHEMLNSVRDFLKLYQ-------------
149708578            --------------GSDILPQYKQEAP----------KTPPHIILH-YCVFKTTWDWIILILTFYTAILVPYNVSFKT--------------------------------------------------------------------------------------------------------------------------------------------------------------------------------------------------------------------RQNN-----V---------------------------------AWLVVDSIVDVIFLVDIVLNFHTTFVGPAG---EVISD-PKLIRMNYLKTW---------------------FVIDLLSCLPYDVINAFENVD---------------------------------------------------------------EVSAFMGDPGKIGFADQIPPPLEGRESQGI----------------------SSLFSSLKVVRLLRLGRVA-RKLDHY--------------------IEYGAAVLV--------LLVCVFGLAAH---------WMACIWYSIGDY--EIFDEDTKT-----------------------------------------------------------IRNNSWLYQLAMDIGTPYQFNGSGSGK--------------------------------------------------WEGGPSKNSVYISSLYFTMTSLTSVGFGNIAPSTDIEKIFA-VAIMMIGSLLYATIFGNVTTIFQQMYANTNRYHEMLNSVRDFLKLYQ-------------
149041059            --------------GSDILPQYKQEAP----------KTPPHIILH-YCVFKTTWDWIILILTFYTAILVPYNVSFKT--------------------------------------------------------------------------------------------------------------------------------------------------------------------------------------------------------------------RQNN-----V---------------------------------AWLVVDSIVDVIFLVDIVLNFHTTFVGPAG---EVISD-PKLIRMNYLKTW---------------------FVIDLLSCLPYDVINAFENVD---------------------------------------------------------------EVSAFMGDPGKIGFADQIPPPLEGRESQGI----------------------SSLFSSLKVVRLLRLGRVA-RKLDHY--------------------IEYGAAVLV--------LLVCVFGLAAH---------WMACIWYSIGDY--EIFDEDTKT-----------------------------------------------------------IRNNSWLYQLALDIGTPYQFNGSGSGK--------------------------------------------------WEGGPSKNSVYISSLYFTMTSLTSVGFGNIAPSTDIEKIFA-VAIMMIGSLLYATIFGNVTTIFQQMYANTNRYHEMLNSVRDFLKLYQ-------------
6625694              --------------GSDILPQYKQEAP----------KTPPHIILH-YCAFKTTWDWVILILTFYTAIMVPYNVSFKT--------------------------------------------------------------------------------------------------------------------------------------------------------------------------------------------------------------------KQNN-----I---------------------------------AWLVLDSVVDVIFLVDIVLNFHTTFVGPGG---EVISD-PKLIRMNYLKTW---------------------FVIDLLSCLPYDIINAFENVD------------------------------------------------------------------------------------------EGI----------------------SSLFSSLKVVRLLRLGRVA-RKLDHY--------------------LEYGAAVLV--------LLVCVFGLVAH---------WLACIWYSIGDY--EVIDEVTNT-----------------------------------------------------------IQIDSWLYQLALSIRTPYRYN-TSAGI--------------------------------------------------WEGGPSKDSLYVSSLYFTMTSLTTIGFGNIAPTTDVEKMFS-VAMMMVGSLLYATIFGNVTTIFQQMYANTNRYHEMLNNVRDFLKLYQ-------------
148704544            --------------GSDILPQYKQEAP----------KTPPHIILH-YCAFKTTWDWVILILTFYTAIMVPYNVSFKT--------------------------------------------------------------------------------------------------------------------------------------------------------------------------------------------------------------------KQNN-----I---------------------------------AWLVLDSVVDVIFLVDIVLNFHTTFVGPGG---EVISD-PKLIRMNYLKTW---------------------FVIDLLSCLPYDIINAFENVD------------------------------------------------------------------------------------------EGI----------------------SSLFSSLKVVRLLRLGRVA-RKLDHY--------------------LEYGAAVLV--------LLVCVFGLVAH---------WLACIWYSIGDY--EVIDEVTNT-----------------------------------------------------------IQIDSWLYQLALSIGTPYRYN-TSAGI--------------------------------------------------WEGGPSKDSLYVSSLYFTMTSLTTIGFGNIAPTTDVEKMFS-VAMMMVGSLLYATIFGNVTTIFQQMYANTNRYHEMLNNVRDFLKLYQ-------------
157312               --------------SADIMPQYRQEAP----------KTPPHILLH-YCAFKAIWDWVILCLTFYTAIMVPYNVAFKN-------------------------------------------------------------------------------------------------------------------------------------------------------------------------------------------------------------------KTSED-----V---------------------------------SLLVVDSIVDVIFFIDIVLNFHTTFVGPGG---EVVSD-PKVIRMNYLKSW---------------------FIIDLLSCLPYDVFNAFDRDE------------------------------------------------------------------------------------------DGI----------------------GSLFSALKVVRLLRLGRVV-RKLDRY--------------------LEYGAAMLI--------LLLCFYMLVAH---------WLACIWYSIGRS------DADN------------------------------------------------------------GIQYSWLWKLANVTQSPYSYIWSNDT-GP-----------------------------------------------ELVNGPSRKSMYVTALYFTMTCMTSVGFGNVAAETDNEKVFT-ICMMIIAALLYATIFGHVTTIIQQMTSATAKYHDMLNNVREFMKLHE-------------
108884339            --------------SADVMPQYRQEAP----------KTPPHILLH-YCAFKAIWDWVILCLTFYTAIMVPYNVAFKN-------------------------------------------------------------------------------------------------------------------------------------------------------------------------------------------------------------------KTSED-----V---------------------------------SLLVVDSIVDVIFFIDIVLNFHTTFVGPGG---EVVSD-PKVIRMNYLKSW---------------------FIIDLLSCLPYDVFNAFDHDE------------------------------------------------------------------------------------------DGI----------------------GSLFSALKVVRLLRLGRVV-RKLDRY--------------------LEYGAAMLI--------LLLCFYMLVAH---------WLACIWYSIGRS------DADN------------------------------------------------------------GVQYSWLWKLANVTQSPYSYIWSNESNAP-----------------------------------------------ELVNGPSRKTMYVTALYFTMTCMTSVGFGNVAAETDNEKVFT-ICMMIIAALLYATIFGHVTTIIQQMTSATAKYHDMLNNVREFMKLHE-------------
71989969             --------------GGDMLPQYRQETP----------KTSPHIILH-YSSFKTIWDWSILALTFYTAFMVPFNIAFKN----------------------------------------------------------------------------------------------------------------------------------------------------------------------LRPFYLIS-------------------------------------SSRENPGG-GID--------------------------------SVALMDSIVDVIFFADILLNFHTTFVGPGG---EVVIE-PSVIRQNYFKSW---------------------FLIDLLSCLPYDIFYMFKRDD------------------------------------------------------------------------------------------ERI----------------------GSLFSALKVVRLLRLGRVA-RKLDNY--------------------LEYGAATLL--------LLLCAYVIVAH---------WLACVWFWIGDS--EVRLKMDNL-----------------------------------------------------------ALPDGWLWKLSNDLRQHYNIPLSNKT--------------------------------------------------TLVGGPSRTSAYISSLYYTMSCMSTVGFGNIASNTDNEKIFG-VCMMIISALLYAAIFGHMTTIIQQMTSSTVRYHEMISNVREFIKLQE-------------
4731355              --------------GGDMLPQYRQETP----------KTSPHIILH-YSSFKTIWDWSILALTFYTAFMVPFNIAFKN---------------------------------------------------------------------------------------------------------------------------------------------------------------------SLRPFYL--------------------------------------ISSRENPGG-GID--------------------------------SVALMDSIVDVIFFADILLNFHTTFVGPGG---EVVIE-PSVIRQNYFKSW---------------------FLIDLLSCLPYDIFYMFKRDD------------------------------------------------------------------------------------------ERI----------------------GSLFSALKVVRLLRLGRVA-RKLDNY--------------------LEYGAATLL--------LLLCAYVIVAH---------WLACVWFWIGDS--EVRLKMDNL-----------------------------------------------------------ALPDGWLWKLSNDLRQHYNIPLSNKT--------------------------------------------------TLVGGPSRTSAYISSLYYTMSCMSTVGFGNIASNTDNEKIFG-VCMMIISALLYAAIFGHMTTIIQQMTSSTVRYHEMISNVREFIKLQE-------------
108875949            --------------GSKKALVKERIRQ----------KTSGHWVIHPCSSFRFYWDLCMLLLLVANLIILPVAISFFN-------------------------------------------------------------------------------------------------------------------------------------------------------------------------------------------------------------------DDLST---------------------------------------RWIAFNCLSDTIFLVDIVVNFRTG-IMQQD-NAEQVILDPKLIAKHYLKTW---------------------FFLDLISSIPLDYIFLIFN--------------------------------------------------------------------------------------------QDFSD----------SFQLLHAGRALRILRLAKLLSLVRLLRLS-RLVRYV----------SQWEEVY--FLNMASVFMRIF------NLICMMLLIGH---------WSGCLQFLVPMLQ-GFPSN-------------------------------------------------------------------SWVAIN-------------------------------------------------------------------ELQESYWLEQYSWALFKAMSHMLCIGYGRFPPQSLTDMWLT-MLSMISGATCYALFLGHATNLIQSLDSSRRQYREKVKQVEEYMAYRK-------------
5326833              --------------GSRKALVKERIRQ----------KTSGHWVIHPCSSFRFYWDLCMLLLLVANLIILPVAISFFN-------------------------------------------------------------------------------------------------------------------------------------------------------------------------------------------------------------------DDLST---------------------------------------RWIAFNCLSDTIFLIDIVVNFRTG-IMQQD-NAEQVILDPKLIAKHYLRTW---------------------FFLDLISSIPLDYIFLIFNQIMKL---------------------------------------------------------------------------------------QDFSD----------SFQILHAGRALRILRLAKLLSLVRLLRLS-RLVRYV----------SQWEEVY--FLNMASVFMRIF------NLICMMLLIGH---------WSGCLQFLVPMLQ-GFPSN-------------------------------------------------------------------SWVSIN-------------------------------------------------------------------ELQESYWLEQYSWALFKAMSHMLCIGYGRFPPQSLTDMWLT-MLSMISGATCYALFLGHATNLIQSLDSSRRQYREKVKQVEEYMAYRK-------------
3970750              --------------GSKKALMKERIRQ----------KAAGHWVIHPCSSFRFYWDLCMLLLLVANLIILPVAISFFN-------------------------------------------------------------------------------------------------------------------------------------------------------------------------------------------------------------------DDLST---------------------------------------RWIAFNCLSDTIFLIDIVVNFRTG-IMQQD-NAEQVILDPKLIAKHYLRTW---------------------FFLDLISSIPLDYIFLIFN--------------------------------------------------------------------------------------------QDFSE----------SFQILHAGRALRILRLAKLLSLVRLLRLS-RLVRYV----------SQWEEVY--FLNMASVFMRIF------NLICMMLLIGH---------WSGCLQFLVPMLQ-GFPPN-------------------------------------------------------------------SWVAIN-------------------------------------------------------------------ELQEAYWLEQYSWALFKAMSHMLCIGYGRFPPQSLTDMWLT-MLSMISGATCYALFLGHATNLIQSLDSSRRQYREKVKQVEEYMAYRK-------------
47551101             --------------GSKKGLQKEKYRL----------RKAGVLIIHPCSHFRFYWDLLMLCLIMANVILLPVVITFFHN------------------------------------------------------------------------------------------------------------------------------------------------------------------------------------------------------------------KDMST---------------------------------------GWLIFNCFSDTFFILDLICNFRTG-IMNPK-SAEQVILNPRQIAYHYLRSW---------------------FIIDLVSSIPMDYIFLLAGG-------------------------------------------------------------------------------------------QNRH--------------FLEVSRALKILRFAKLLSLLRLLRLS-RLMRFV----------SQWEQA----FNVANAVIRIC------NLVCMMLLIGH---------WNGCLQYLVPMLQ-EYPDQ-------------------------------------------------------------------SWVAIN-------------------------------------------------------------------GLEHAHWWEQYTWALFKALSHMLCIGYGKFPPQSITDVWLT-IVSMVSGATCFALFIGHATNLIQSMDSSSRQYREKLKQVEEYMQYRK-------------
16758502             --------------GSHKAVEIEQERV----------KSAGAWIIHPYSDFRFYWDLIMLLLMVGNLIVLPVGITFFK-------------------------------------------------------------------------------------------------------------------------------------------------------------------------------------------------------------------EENSP---------------------------------------PWIVFNVLSDTFFLLDLVLNFRTG-IVVEE-GAE-ILLAPRAIRTRYLRTW---------------------FLVDLISSIPVDYIFLVVELE------------------------------------------------------------------------------------------PRL------------DAEVYKTARALRIVRFTKILSLLRLLRLS-RLIRYM----------HQWEEIFHMTYDLASAVVRIF------NLIGMMLLLCH---------WDGCLQFLVPMLQ-DFPSD-------------------------------------------------------------------CWVSMN-------------------------------------------------------------------RMVNHSWGRQYSHALFKAMSHMLCIGYGQQAPVGMPDVWLT-MLSMIVGATCYAMFIGHATALIQSLDSSRRQYQEKYKQVEQYMSFHK-------------
6680191              --------------GSHKAVEIEQERV----------KSAGAWIIHPYSDFRFYWDLIMLLLMVGNLIVLPVGITFFK-------------------------------------------------------------------------------------------------------------------------------------------------------------------------------------------------------------------EENSP---------------------------------------PWIVFNVLSDTFFLLDLVLNFRTG-IVVEE-GAE-ILLAPRAIRTRYLRTW---------------------FLVDLISSIPVDYIFLVVELE------------------------------------------------------------------------------------------PRL------------DAEVYKTARALRIVRFTKILSLLRLLRLS-RLIRYI----------HQWEEIFHMTYDLASAVVRIF------NLIGMMLLLCH---------WDGCLQFLVPMLQ-DFPSD-------------------------------------------------------------------CWVSMN-------------------------------------------------------------------RMVNHSWGRQYSHALFKAMSHMLCIGYGQQAPVGMPDVWLT-MLSMIVGATCYAMFIGHATALIQSLDSSRRQYQEKYKQVEQYMSFHK-------------
149751569            --------------GSHKAVEIEQERV----------KSAGAWIIHPYSDFRFYWDLIMLLLMVGNLIVLPVGITFFK-------------------------------------------------------------------------------------------------------------------------------------------------------------------------------------------------------------------EENSP---------------------------------------PWIVFNVLSDTFFLLDLVLNFRTG-IVVEE-GAE-ILLAPRAIRTRYLRTW---------------------FLVDLISSIPVDYIFLVVELE------------------------------------------------------------------------------------------PRL------------DAEVYKTARALRIVRFTKILSLLRLLRLS-RLIRYI----------HQWEEIFHMTYDLASAVVRIF------NLIGMMLLLCH---------WDGCLQFLVPMLQ-DFPPD-------------------------------------------------------------------CWVSIN-------------------------------------------------------------------HMVNHSWGRQYSHALFKAMSHMLCIGYGQQAPVGMPDVWLT-MLSMIVGATCYAMFIGHATALIQSLDSSRRQYQEKYKQVEQYMSFHK-------------
124487125            --------------GSQKAVEREQERV----------KSAGFWIIHPYSDFRFYWDLTMLLLMVGNLIIIPVGITFFK-------------------------------------------------------------------------------------------------------------------------------------------------------------------------------------------------------------------DENTT---------------------------------------PWIVFNVVSDTFFLIDLVLNFRTG-IVVED-NTE-IILDPQRIKMKYLKSW---------------------FVVDFISSIPVDYIFLIVETR------------------------------------------------------------------------------------------IDS--------------EVYKTARALRIVRFTKILSLLRLLRLS-RLIRYI----------HQWEEIFHMTYDLASAVVRIV------NLIGMMLLLCH---------WDGCLQFLVPMLQ-DFPHD-------------------------------------------------------------------CWVSIN-------------------------------------------------------------------GMVNNSWGKQYSYALFKAMSHMLCIGYGRQAPVGMSDVWLT-MLSMIVGATCYAMFIGHATALIQSLDSSRRQYQEKYKQVEQYMSFHK-------------
29840776             --------------GSQKAVEREQERV----------KSAGFWIIHPYSDFRFYWDLTMLLLMVGNLIIIPVGITFFK-------------------------------------------------------------------------------------------------------------------------------------------------------------------------------------------------------------------DENTT---------------------------------------PWIVFNVVSDTFFLIDLVLNFRTG-IVVED-NTE-IILDPQRIKMKYLKSW---------------------FVVDFISSIPVEYIFLIVETR------------------------------------------------------------------------------------------IDS--------------EVYKTARAVRIVRFTKILSLLRLLRLS-RLIRYI----------HQWEEIFHMTYDLASAVVRIV------NLIGMMLLLCH---------WDGCLQFLVPMLQ-DFPHD-------------------------------------------------------------------CWVSIN-------------------------------------------------------------------GMVNNSWGKQYSYALFKAMSHMLCIGYGRQAPVGMSDVWLT-MLSMIVGATCYAMFIGHATALIQSLDSSRRQYQEKYKQVEQYMSFHK-------------
5734516              --------------GSQKAVEREQERV----------KSAGFWIIHPYSDFRFYWDLTMLLLMVGNLIIIPVGITFFK-------------------------------------------------------------------------------------------------------------------------------------------------------------------------------------------------------------------DENTT---------------------------------------PWIVFNVVSDTFFLIDLVLNFRTG-IVVED-NTE-IILDPQRIKMKYLKSW---------------------FMVDFISSIPVDYIFLIVETR------------------------------------------------------------------------------------------IDS--------------EVYKTARALRIVRFTKILSLLRLLRLS-RLIRYI----------HQWEEIFHMTYDLASAVVRIV------NLIGMMLLLCH---------WDGCLQFLVPMLQ-DFPDD-------------------------------------------------------------------CWVSIN-------------------------------------------------------------------NMVNNSWGKQYSYALFKAMSHMLCIGYGRQAPVGMSDVWLT-MLSMIVGATCYAMFIGHATALIQSLDSSRRQYQEKYKQVEQYMSFHK-------------
130502156            --------------GSQKAVEREQERV----------KSAGFWIIHPYSDFRFYWDLTMLLLMVGNLIIIPVGITFFK-------------------------------------------------------------------------------------------------------------------------------------------------------------------------------------------------------------------DENTT---------------------------------------PWIVFNVVSDTFFLIDLVLNFRTG-IVVED-NTD-IILDPRRIKMKYLKSW---------------------FVVDFVSSIPVDYIFLIVETR------------------------------------------------------------------------------------------IDS--------------EVYKTARALRIVRFTKILSLLRLLRLS-RLIRYI----------HQWEEIFHMTYDLASAVVRIV------NLIGMMLLLCH---------WDGCLQFLVPMLQ-DFPDD-------------------------------------------------------------------CWVSLN-------------------------------------------------------------------NMVNNSWGKQYSYALFKAMSHMLCIGYGRQAPMGMSDVWLT-MLSMIVGATCYAMFIGHATALIQSLDSSRRQYQEKYKQVEQYMSFHK-------------
50878267             --------------GSQKAVEREQERV----------KSAGAWIIHPYSDFRFYWDFTMLLFMVGNLIIIPVGITFFK-------------------------------------------------------------------------------------------------------------------------------------------------------------------------------------------------------------------DETTA---------------------------------------PWIVFNVVSDTFFLMDLVLNFRTG-IVIED-NTE-IILDPEKIKKKYLRTW---------------------FVVDFVSSIPVDYIFLIVEKG------------------------------------------------------------------------------------------IDS--------------EVYKTARALRIVRFTKILSLLRLLRLS-RLIRYI----------HQWEEIFHMTYDLASAVMRIC------NLISMMLLLCH---------WDGCLQFLVPMLQ-DFPSD-------------------------------------------------------------------CWVSIN-------------------------------------------------------------------NMVNHSWSELYSFALFKAMSHMLCIGYGRQAPESMTDIWLT-MLSMIVGATCYAMFIGHATALIQSLDSSRRQYQEKYKQVEQYMSFHK-------------
7407647              --------------GSQKAVEREQERV----------KSAGAWIIHPYXDFRFYWDFTMLLFMVGNLIIIPVGITFFK-------------------------------------------------------------------------------------------------------------------------------------------------------------------------------------------------------------------DETTA---------------------------------------PWIVFNVVSDTFFLMDLVLNFRTG-IVIED-NTE-IILDPEKIKKKYLRTW---------------------FVVDFVSSIPVDYIFLIVEKG------------------------------------------------------------------------------------------IDS--------------EVYKTARALRIVRFTKILSLLRLLRLS-RLIRYI----------HQWEEIFHMTYDLASAVMRIC------NLISMMLLLCH---------WDGCLQFLVPMLQ-DFPSD-------------------------------------------------------------------CWVSIN-------------------------------------------------------------------NMVNHSWSELYSFALFKAMSHMLCIGYGRQAPESMTDIWLT-MLSMIVGATCYAMFIGHATALIQSLDSSRRQYQEKYKQVEQYMSFHK-------------
108935843_146_584    --------------GSQKAVEREQERV----------KSAGAWIIHPYSDFRFYWDFTMLLFMVGNLIIIPVGITFFK-------------------------------------------------------------------------------------------------------------------------------------------------------------------------------------------------------------------DETTA---------------------------------------PWIVFNVVSDTFFLMDLVLNFRTG-IVIED-NTE-IILDPEKIKKKYLRTW---------------------FVVVFVSSIPVDYIFLIVEKG------------------------------------------------------------------------------------------IDS--------------EVYKTARALRIVRFTKILSLLRLLRLS-RLIRYI----------HQWEEIFHMTYDLASAVMRIC------NLISMMLLLCH---------WDGCLQFLVPMLQ-DFPRN-------------------------------------------------------------------CWVSIN-------------------------------------------------------------------GMVNHSWSELYSFALFKAMSHMLCIGYGRQAPESMTDIWLT-MLSMIVGATCYAMFIGHATALIQSLDSSRRQYQEKYKQVEQYMSFHK-------------
6754168              --------------GSQKAVEKEQERV----------KTAGFWIIHPYSDFRFYWDLIMLIMMVGNLVIIPVGITFFT-------------------------------------------------------------------------------------------------------------------------------------------------------------------------------------------------------------------EQTTT---------------------------------------PWIIFNVASDTVFLLDLIMNFRTG-TVNED-SSE-IILDPKVIKMNYLKSW---------------------FVVDFISSIPVDYIFLIVEKG------------------------------------------------------------------------------------------MDS--------------EVYKTARALRIVRFTKILSLLRLLRLS-RLIRYI----------HQWEEIFHMTYDLASAVVRIF------NLIGMMLLLCH---------WDGCLQFLVPLLQ-DFPPD-------------------------------------------------------------------CWVSLN-------------------------------------------------------------------EMVNDSWGKQYSYALFKAMSHMLCIGYGAQAPVSMSDLWIT-MLSMIVGATCYAMFVGHATALIQSLDSSRRQYQEKYKQVEQYMSFHK-------------
3810676              ------------GSGGKFTVINGIR--D---------ISRGSIVHPD-NRWYKAWTMFILIWALYSSFFTPLEFGFFR--------------------------------------------------------------------------------------------------------------------------------------------------------------------------------------------------------------------GLPE---------------------------------------NLFILDIAGQIAFLVDIVLTFFVAYRDSRT---YRMIYKRSSIALRYLKST---------------------FIIDLLACMPWDIIYKAAGEK------------------------------------------------------------------------------------------EEV-----------------------RYLLLIRLYRVHRVILFFHKMEKD-------------------IRINYLFTRIV--------KLIFVELYCTH------------TAACIFYYLATTLPASQE---------------------------------------------------------------GYTWIGSLKLGDYS-----------------------------------------------------------YSKFREIDLWTRYTTSMYFAVVTMATVGYGDIHAVNMREMIFA-MVYISFDMILGAYLIGNMTALIVKGS-KTERFRDKMADIMRYMNRNK-------------
10443349             ------------QRKLSRYVLDGIR--G---------FSKDFIIHPEKNRWYQAWTKFILLWAIYSSFFTPMEFGFFR--------------------------------------------------------------------------------------------------------------------------------------------------------------------------------------------------------------------GLDK---------------------------------------DLFILDIIGQVAFLVDIVVQFCLAYRDSQT---YRMIYKPSSIALRYLKSS---------------------FIFDLLGCMPWDIIYKASGRK------------------------------------------------------------------------------------------EGV-----------------------RYLLLIRLYRVRKVIEFFQRMEKD-------------------IRINYMFTRIV--------KLLVVELYCTH------------TAACIFYYLATTLPASQE---------------------------------------------------------------GYTWIGSLKLGDYS-----------------------------------------------------------YMNFRDIDLWKRYTTSLYFAIVTMATVGYGDIHAVNMREMIFI-MIYVSFDMVLGAYLIGNMTALIVKGS-KTEKFRDRMTDLLKYMNRNR-------------
9955728              --------------GIVFRDGSGLGQSS---------VSDAYIIRPD-SWRYTVWVHFILIWAVYSSFFTPLEFGFFR--------------------------------------------------------------------------------------------------------------------------------------------------------------------------------------------------------------------GLPE---------------------------------------NLFLLDIAGQIAFLIDIVVHFFVAYRATHS---YRLVCRHKLIAIRYLKSR---------------------FLVDFLGCLPWDAIFKVSGRK------------------------------------------------------------------------------------------EAV-----------------------RYMLWIRLSRAKRVSEFFERLEKD-------------------IRINYLFTRIV--------KLLVVELYCTH------------TAACIFYYLATTMPPSQE---------------------------------------------------------------GYTWIGSLQMGDYH-----------------------------------------------------------YTHFREIDLWKRYITSLYFAIVTMATVGYGEIHAVNVREMIFV-MVYVSFDMILGAYLLGNMTALIVKGS-KTEKFRDRMTDLIKYMNRNN-------------
145497983            --------------AHANNPLWWRFWCF---------IKSDETVLLPADKFLFLWDLLMMLVTIVNIFYVPLQLSFNLNEDN-----------------------------------------------------------------------------------------------------------------------------------------------------------------------------------------------------------------MGS---------------------------------------VFTLFSTLPSCIFLIDLILTFFKG-YYDRG----ILQRNKAKIFWHYIKGD---------------------FALDLAIVLPFILSWMGYSAA------------------------------------------------------------------------------------------NYL--------------------------MLIRMTRVRRTMIVI-EEISNF--------------------KEKSAIIYQLF------CLIYSLLLISH------------FCACLFHYFALYEVDQGY---------------------------------------------------------------THTWLHQQN-------------------------------------------------------------------IFDEDLYTKYFNSLYWITITSMTVGYGDIVPVTTPEKILV-TVITFLVTGVFGYALGMIQSIFYKMAEQTNLNNSRLRLVSNHIKQRG-------------
17030694             --------------AHASNPLWWRFWCF---------LKSDETVLLPADKFLFVWDLLMMLVTIANIFYVPLQLSFNLNEDD-----------------------------------------------------------------------------------------------------------------------------------------------------------------------------------------------------------------MGS---------------------------------------IFTLFSTLPSCIFLIDLILTFFKG-YYDRG----ILQRNKAKIFRHYIKGD---------------------FLLDLAIVLPFILSWMGYSAA------------------------------------------------------------------------------------------NYL--------------------------MLIRMTRVRRTMIVI-EEISNF--------------------KEKSAIIYQLF------CLIYSLLLISH------------FCACLFHYFALYEVDQGY---------------------------------------------------------------THTWLHQQN-------------------------------------------------------------------IFDEDLYTKYFNSLYWITITSMTVGYGDIVPVTTPEKILV-TIITFLVTGVFGYALGMIQSIFYEMAEQTNINNSRLRLVSNHIKQRG-------------
14601099_20_264      ------------MARFRRGLSDLG-------------GRVRNIGDVMEHPLVELGVSYAALLSVIVVVVEYTMQLS------------------------------------------------------------------------------------------------------------------------------------------------------------------------------------------------------------------------------------------------------------GEYLVRLYLVDLILVIILWADYAYRAYKS-----------------GDPAGYVKK----------------------TLYEIPALVPAGLLALIEGHLAGL-----------------------------------------------------------------------------------------------------------------GLFRLVRLLRFLRILLIISRGS-----------------------KFLSAIAD---------AADKIRFYHLFGA----VMLTVLYGAFAIYIVEYPDPN--------------------------------------------------------------------------------------------------------------------------------------------------SSIKSVFDALWWAVVTATTVGYGDVVPATPIGKVIG-IAVMLTGISALTLLIGTVSNMFQKILVG--------------------------------
33863295             ----------GSIMGFRLKLHEQ-------------VINQSTGG---NTSIFNKLSGATIAVSIFFAVLST----ENQIDFQ--------------------------------------------------------------------------------------------------------------------------------------------------------------------------------------------------------------------------------------------------------FGGQIDAIDWIIGCLFCIEYFCRVWTA-------PLEGKYGEGIEGTIRYMFSPM--------------------AIIDLIAIIPSFIGVRTELKIL----------------------------------------------------------------------------------------------------------------------RIIRLLTILKVGRSE-RFKQSM--------------------LHFNYALRS--------KSQELQISTVYT------LLLLLISSTFMYLAESS--------------------------------------------------------------------------IQPD------------------------------------------------------------------------LLGSIPRCLWWSINAVSSVGHGDSVPISAVGKIIA-SVTSLMGIGAIAIPTGILAAGFSESIAV--------------------------------
25147245             --------------RIRLKVYNFLEKP-----------------LNAASAPYHFFIFGLVIANIILGAATNDNDSTVSK--------------------------------------------------------------------------------------------------------------------------------------------------------------------------------------------------------------------------------------------------------------IHFFLEIFMIVFFILEFAVRLWSV-------------RADAKYRTKYGRLYYLFHT---------------VTLIDILIIPATILLLVFKGHDVDGSTL--------------------------------------------------------------------------------------------------------------DTL---RFIQILRLFHVD-RQMATW--------------------KLLRKMIILG--------KWQLMATYYIT------LVVGLSLATIVYSTEALAQG----------------------------------------------------------------------IQDNGYGLI-------------------------------------------------------------VPEGTNATFPTMAHSWWFTAVTVMTVGYGDIYPVGALTKFLV-CVLGFIAFCTFQAANTQISVGLTLMMEE--------------------------------
117926954            --------------PIKTLFFHLLEDP-----------SSRAR------NAFSLLMMLVVFASLIAMTLETDPDLTLEEQ-------------------------------------------------------------------------------------------------------------------------------------------------------------------------------------------------------------------L-----------------------------------------FFDFLDHAFNIIFLMEYMLRWWVCSDFLVDFEESFQRQRRMSHRRKHHQALFKALRAAFRPKLLWMRKP--LSIIDLLAILPIFRAF--------------------------------------------------------------------------------------------------------------------------RLLRILRVLRILKLFRYSKRLTFF--------------------SSVIAER-----------SFELTSLFTLA--------AVVLGMVTLAFFMAER-------------------------------------------------------------------------GHNPD-------------------------------------------------------------------------ITNLWDALYWTMITITTVGYGDISPATDGGRMVA-ITGTIVGIWVTVLMTSIIVSALTERIFELKEQRMERQIEKLRDHFIICG-----------
15606902_1_237       ----------------RKLLYELL--------------EDDRS---FVFYIYNTFSFSVILISVILTLYDEFYGFHSKLHP------------------------------------------------------------------------------------------------------------------------------------------------------------------------------------------------------------------------------------------------------------VVLEIEWVASGIIAFELIGRYILA-----------------ENKLKYLTNSL--------------------TILDFIALIPTFQIFRAVR-------------------------------------------------------------------------------------------------------------------------LIVLIARVLRLTYRY-RF------FF----------------GFFANIIKEF-------AFELIFVFALI---------LVVIFSILLIVFSVEHGGG------------------------------------------------------------------------------------------------------------------------------------------------NEKINTFFDALYYVIITATTVGYGDITPTTPLGKALA-MVLGVLGLFLFSLITATVSTAFFHYVNM--------------------------------
113478270            -------------TILKIPIFAP------------------------DSKFIRLWEILIVFITVYNAFLIPFRISFEKR-------------------------------------------------------------------------------------------------------------------------------------------------------------------------------------------------------------------FYG----------------------------------------IWILLDLIGDVILIADIFLRFHLG-----YFEHGEYVEDKKKIAQRYFYKL---------------------LRRHLIASFPGDLIARILLPNSLF---------------------------------------------------------------------------------------------------------------IIGLCRCPRLLRLPQFYRIFNR--------------------------ETNINI-----EPTLIRMCKLIIFIALITH---------WVACGWFLIGSWESN----------------------------------------------------------------------FGESWLINKS--------------------------------------------------------------LKSVGTRTQYINCLYWAITTLTTVGYGDITPTTEIEIIFT-LMVMFLGISMYAYTIGNVSSLISNLDAAQARYREKLHQIKTYMRENK-------------
15828892_36_301      -----------NNVAINEKSKEKD-------------QPKVNLIVDVLRKIWFIVVLSFIILSFLLLIIDKKFIASNKSFFL--------------------------------------------------------------------------------------------------------------------------------------------------------------------------------------------------------------------------------------------------------------VIELTIFFVLLVDFFLWTFTYPYRKN------------GKLISMIIFPF-----------------TFTGILLVSSFVPSIWILEAFIENKDINVESQ----------------------------------------------------------------------------------------------------NNKVPKTFEISQVLSFLRSLVFLRVG-RVL---MLLQVFE-----------PFKILYRAFKN--------EKKILISLFVFI------IILILIFSLIIYSVETIGE-----------------------------------------------------------------------NANPN-------------------------------------------------------------------------IKNYWDAIYFTTISMTTIGYGDISPVTTNGKIII-VIISIIGIALFAIPSGVIAGAFLAEIQK--------------------------------
```
